# Large Language Models for Oncology Guideline Maintenance: Prospective Case Study

**DOI:** 10.2196/93239

**Published:** 2026-08-20

**Authors:** Manuel Knauer, Julian Greß, Jakob Nikolas Kather, Peter May

**Affiliations:** 1 Department of Medicine III, TUM School of Medicine and Health, Technical University of Munich, Ismaninger Str. 22, Munich, Bavaria, 81675, Germany, +49 8941408753; 2 Else Kroener Fresenius Center for Digital Health, Technische Universität Dresden, Dresden, Saxony, Germany; 3 Department of Medicine I, Faculty of Medicine, Technische Universität Dresden, Dresden, Saxony, Germany; 4 Medical Oncology, National Center for Tumor Diseases, Heidelberg, Baden-Wurttemberg, Germany

**Keywords:** large language model, artificial intelligence, AI, clinical practice guidelines, oncology, quality control

## Abstract

**Background:**

Maintenance of oncology clinical practice guidelines (CPGs) is increasingly challenged by the rapid growth of trial data and therapeutic complexity. While large language models (LLMs) have shown promise in information retrieval, their utility in the rigorous, end-to-end workflow of guideline maintenance remains underexplored.

**Objective:**

This case study aimed to systematically evaluate the performance of frontier LLMs in supporting oncology guideline maintenance. We sought to determine their reliability in predicting necessary guideline updates based on new evidence, their accuracy in extracting data from clinical trials, and their effectiveness as automated auditors for detecting errors in established guidelines.

**Methods:**

Using the Onkopedia peripheral T-cell lymphoma (PTCL) guideline as a prospective case study, we tasked frontier models with deep-research modes and autonomous web-search capabilities (Gemini 2.5 Pro and GPT o4-mini-high) to predict a guideline update in August 2025 based on the 2021 version. Predictions were validated against the official 2025 revision published in October 2025. Next, we benchmarked evidence extraction accuracy across 80 pivotal trials using models of varying scale (27B-671B parameters vs frontier). Finally, we deployed a stacked LLM workflow to audit 28 recently updated Onkopedia guidelines for linguistic and content-related errors.

**Results:**

In the predictive task, models captured 36.7% to 40% of substantive updates, often identifying landmark approvals, but frequently overstating evidence. An independent, model-blinded rescoring yielded substantial agreement (weighted Cohen κ=0.75) and confirmed predictive accuracies of 35% to 38.3%. While frontier models demonstrated high accuracy (up to 99.2%) in extracting data from individual studies, substantially outperforming smaller open-source models, this precision declined during multisource synthesis. We observed a position-dependent performance drop in long-form generation, with GPT’s endpoint accuracy dropping from 84.6% in the first half of the drafted guideline to 38.5% in the second half. As automated auditors of existing CPGs, the models successfully identified a median of 16.5 (IQR 13.8-20.3) formal errors per document and detected several clinically relevant inconsistencies (eg, invalid scoring formulas and incorrect staging definitions).

**Conclusions:**

LLMs currently lack the reasoning stability for autonomous guideline authoring due to deficits in complex synthesis. However, they are effective tools for high-fidelity evidence extraction and automated quality assurance, supporting a human-led, AI-augmented workflow for efficient guideline maintenance.

## Introduction

Large language models (LLMs) are increasingly evaluated for use in oncology, with potential use cases ranging from patient education and documentation to complex clinical decision support or evidence synthesis [1-3]. Recent reviews underscore that LLMs can achieve high concordance with guideline-based recommendations in selected scenarios and streamline literature processing, yet emphasize persistent challenges regarding hallucinations, explainability, and safety-critical deployment [4-7]. In parallel, professional societies have begun to formalize governance, with the European Society for Medical Oncology recently publishing the first structured guidance on the safe use of LLMs in oncology practice [8].

Clinical practice guidelines (CPGs) are potentially well suited to LLM-based augmentation. CPGs translate high-quality evidence into actionable recommendations to optimize patient care. They are typically preceded by a technical process of systematically searching for, selecting, and appraising evidence [9,10]. Collectively, these methodological steps make CPG development resource-intensive and time-consuming [11,12]. Especially in the field of oncology, guideline programs face accelerating trial output, increasing therapeutic complexity, rapid label and reimbursement changes, and expanding biomarker-driven indications, which together increase the frequency and difficulty of guideline maintenance [13,14]. Living guideline models, which provide continuous surveillance with trigger-based updates, have emerged as a preferred approach, yet they require significant operational capacity [15-17].

Previous studies demonstrate that general-purpose and domain-adapted LLMs can retrieve and integrate trial evidence, suggest reasonable treatment options, and support tumor boards, with performance varying across tasks, tumor types, and model families [1,18-21]. However, existing peer-reviewed evidence remains concentrated on cross-sectional “guideline concordance” tasks and guideline-grounded decision support, rather than on guideline authoring and maintenance as an end-to-end workflow. Multiple studies have evaluated whether LLMs can answer vignette-style questions in alignment with a current guideline version or whether retrieval-augmented generation or graph representations can constrain outputs to guideline text and reduce hallucinations [22-25]. In contrast, only one study has evaluated the role of LLMs in CPG creation, finding that in the case of appendicitis, the models were unable to independently perform a systematic literature search or reliably perform screening, data extraction, or risk-of-bias assessment at the time of testing [26].

We aimed to address these gaps by systematically evaluating contemporary LLMs as autonomous agents for oncology guideline maintenance across 3 complementary functions. Specifically, we sought to determine whether these models could independently search for, retrieve, and synthesize practice-changing data without predefined literature inputs. We tested this across three complementary functions: (1) anticipation of guideline updates using peripheral T-cell lymphoma (PTCL) as a prospective case study with a subsequent real-world guideline revision serving as the reference standard, (2) accuracy of clinical trial evidence extraction across models, and (3) LLM-based quality assurance for detecting clinically relevant inconsistencies and content errors in recently updated guidelines. We further characterized qualitative error types and citation behavior in generative tasks, analyzed false-positive predictions, and evaluated the clinical utility of LLMs as automated auditors for detecting errors in established guidelines.

## Methods

### Guideline Update Prediction

To assess whether LLMs can autonomously support guideline maintenance, we conducted a prospective evaluation using the Onkopedia PTCL guideline as a test case. Onkopedia is the official guideline portal of the hematology and medical oncology societies of German-speaking countries. To ensure a true predictive task rather than parametric memorization, we used a strict prospective timeline (Figure S1 in Multimedia Appendix 1). While the human update process began in February 2024, all drafts remained strictly confidential on internal servers. In August 2025, 2 frontier models with deep-research modes and enabled live web-search capabilities, Google’s Gemini 2.5 Pro (“Gemini”) and OpenAI’s GPT o4-mini-high (“GPT”), generated update proposals from the 2021 version (word count: 5167). Predictions were evaluated against the official Onkopedia PTCL revision published later in October 2025. These proprietary frontier models were exclusively selected for this task because the nature of predicting a multiyear update required autonomous web-browsing capabilities and large context windows, which smaller or offline open-source models lack.

We used a 2-stage prompting strategy. A broad trigger prompt was followed by a structured, persona-based instruction defining the LLM as a “specialized clinical research assistant.” The models were instructed to search for practice-changing data, specifically randomized trials, regulatory approvals, and international guidelines published between June 1, 2021, and August 1, 2025. Outputs were structured by clinical section, requiring explicit citations of study details, estimated evidence levels, and specific implications for the guideline text (refer to Multimedia Appendix 2 for the full prompts). The tasks were performed in German.

Two hematology and oncology specialists (MK and PM, with 4.5 and 7.5 years of clinical experience, respectively) identified 30 substantive changes in the official October 2025 PTCL guideline revision (Table S1 in Multimedia Appendix 3). A change was defined as “substantive” if it altered clinical recommendations (eg, new treatment options, updated diagnostic criteria, and changes in evidence grading) or fundamental classifications, whereas purely linguistic, structural, or stylistic edits were excluded. The LLM-generated predictions were then compared against this list and scored for concordance (1=fully predicted, 0.5=partially predicted, and 0=missed). To mitigate potential confirmation bias from this unblinded primary scoring, a third clinical author (JG) independently rescored the outputs while blinded to model identity. Agreement was quantified using a linear weighted Cohen κ.

Additionally, content generated by the LLMs that did not align with the official revision were separately listed. The outputs were qualitatively analyzed for errors, categorized as “overstatement of evidence,” “oversimplification,” “regulatory status errors,” “citation errors,” “hallucinations,” or “wrong data.” A “hallucination” was strictly defined as a completely fabricated study for which the trial name, clinical identifier, or described patient cohort did not exist in the literature, clearly distinguishing it from “citation errors” (factual studies with incorrectly attributed authors or publication dates). Conflation (“chimera”) errors, in which the statistical outcomes of one real trial were attributed to a different, equally real trial, were coded under “wrong data.” Furthermore, to evaluate end point accuracy, denominators represented the absolute total of specific statistical end points (eg, hazard ratios and median survival times) actively generated by each model; an end point was only scored as correct if both the numerical value and its specific clinical context perfectly matched the primary source. Explicitly cited end points, such as progression-free survival, were verified against primary sources, and bibliometric analysis was performed to determine the distribution of cited sources by type and impact factor (2024 score for all journals according to Clarivate Journal Citation Reports).

### Evidence Extraction Accuracy

To isolate the data-retrieval task, we compiled a dataset of 80 landmark studies spanning 4 oncologic and 4 hematologic entities (10 studies per entity), selected by a senior oncologist (PM). On August 30, 2025, we used this dataset to benchmark standard frontier and open-source models. The models evaluated were Gemma 3 27B (Google; gemma-3-27b-it), GPT OSS 20B (OpenAI; gpt-oss-20b), GPT OSS 120B (OpenAI; gpt-oss-120b), Qwen3 235B (Alibaba Cloud; Qwen3-235B-A22B), DeepSeek 3.1 (DeepSeek; DeepSeek-V3.1), Gemini 2.5 Pro (Google; tested with and without internet access), and ChatGPT o4-mini-high (OpenAI; with internet access). All models were accessed via their corresponding user interfaces or through Hugging Face, with temperature and top-p set to 0 (except for GPT o4-mini-high, which ran at locked platform defaults).

Models were provided only with the study name and prompted in English to extract 10 specific variables into a structured format: first author, journal, publication year, disease scope, participant count, main inclusion criteria, primary end points with statistical values, main results, and 1-sentence summary. As an antihallucination guardrail, models were instructed to output “unknown” if the data could not be retrieved. We applied a standardized scoring system to the outputs:

- 1 point (correct): data fully matches the source (note: both ePub and print years were accepted).
- 0.5 points (partially correct): minor deviations, defined as imprecise journal names (eg, Lancet instead of Lancet Haematology), slight inaccuracies in disease definition (eg, first-line vs second-line), participant counts within a +5% to –5% margin, or effect sizes deviating by up to 10 percentage points.
- 0 points (incorrect): empty fields, unknown, or errors exceeding the partial credit margins.

Quantitative measurements of interrater reliability showed an almost perfect agreement between the 2 independent raters (Cohen κ=0.976 and overall percentage agreement 98.7%).

### Automated Quality Assurance

In December 2025, we evaluated the utility of LLMs as automated reviewers for existing CPGs. The test corpus consisted of 28 Onkopedia guidelines updated in 2025 (full list in Table S2 in Multimedia Appendix 3). The full text of each guideline was processed by Gemini and GPT in a stacked manner (ie, GPT seeing Gemini’s flags). Models were prompted with temperature and top-p set to 0 (except for GPT o4-mini-high, which ran at locked platform defaults) and a “medical editor” persona instructed to detect 2 categories of errors: linguistic and formal issues, and content-related errors (refer to Multimedia Appendix 2 for the prompts). This stacked design was deliberately chosen not to isolate individual model performance, but to simulate a rigorous, multiagent consensus workflow mimicking a human “four-eyes principle.” Regarding the initial prompt setup, Gemini processed the raw guideline text de novo to generate a primary list of suspected errors, which was subsequently provided as the “input list” to GPT for critical verification and filtering.

All LLM-flagged errors were manually verified by the authors and classified according to a predefined schema. Linguistic and formal errors were categorized as “grammar and punctuation,” “medical typos,” “general typos,” “inconsistencies,” or “others.” Content-related errors were stratified by clinical impact, ranging from irrelevant (factual errors with no consequence) and light (minor expected clinical relevance) to moderate (indirect impact on patient care) and severe (direct potential negative impact on patient care). The detailed rubric defining the objective clinical criteria for these severity thresholds is provided in Table S3 in Multimedia Appendix 3. All model outputs were evaluated independently by 2 raters, and discrepancies were resolved by consensus.

### Statistical Analysis

Descriptive statistics were used to summarize model performance scores, error rates, and bibliometric distributions. To evaluate performance differences, nonparametric inferential statistics were applied, using the Mann-Whitney *U* test for independent pooled group comparisons and the Wilcoxon signed-rank test for matched model-to-model comparisons (statistical significance defined as *P*<.05). All analyses were performed using GraphPad Prism (version 10.6.1; Dotmatics).

### Ethical Considerations

This study exclusively involved the analysis of LLMs processing publicly available CPGs and previously published clinical trial data. As the research did not involve human participants, animal subjects, or any identifiable patient data, an ethics review board assessment and informed consent were not required, in accordance with institutional guidelines for secondary research on publicly available literature.

## Results

### Prediction of Guideline Updates

Across the 30 substantive changes identified in the 2025 Onkopedia PTCL guideline update, the models showed moderate predictive capability, with GPT achieving a total predictive score of 40% (11/30, 36.7% fully predicted; 2/30, 6.7% partially predicted; and 17/30, 56.7% missed) and Gemini achieving 36.7% (10/30, 33.3% fully predicted; 2/30, 6.7% partially predicted; and 18/30, 60% missed). Importantly, an independent rescoring of the outputs by a third clinical author who was blinded to model identity yielded similar results (GPT: 38.3%; Gemini: 35%) and showed substantial agreement with the original consensus assessment (linear weighted Cohen κ=0.75).

Performance was domain dependent (Table 1 and Table S1 in Multimedia Appendix 3), peaking in the classification domain where GPT correctly predicted all 3 relevant changes, including the updated *5th edition of the World Health Organization Classification of Haematolymphoid Tumours* (*WHO-HAEM5*) and International Consensus Classification (ICC) taxonomies. Performance across first-line and relapsed therapeutic settings was moderate (43.8%‐66.7%), with both models correctly identifying key evidence updates, including the ECHELON-2 5-year survival data or results from VALENTINE-PTCL01. Performance was lower for updates regarding special entities (25%), diagnostics (10%), and radiotherapy (0%). For example, models failed to capture changes in the role of positron emission tomography/computed tomography (PET/CT) in staging or new evidence about radiation dose.

**Table 1.** Prediction accuracy for peripheral T-cell lymphoma guideline updates across clinical domains by model.

Clinical domains	GPT, n (%)^a^	Gemini, n (%)^a^
Classification and biology	3 (100)	3 (66.7)
Diagnostics and staging	5 (10)	5 (10)
First-line therapy	8 (43.8)	8 (56.3)
Relapse therapy and new substances	6 (66.7)	6 (50)
Special entities and situations	4 (25)	4 (25)
Radiotherapy	4 (0)	4 (0)
Overall	30 (40)	30 (36.7)

a Percentages are based on the concordance score (1=fully predicted, 0.5=partially predicted, 0=missed) divided by the total number of changes within each clinical domain.

Analysis of false-positive predictions (Table S4 in Multimedia Appendix 3) revealed a tendency toward premature clinical translation, where models anticipated that emerging molecular subtypes (eg, GATA3/TBX21 or DUSP22/TP63) would immediately dictate risk-adapted treatment algorithms; in contrast, the actual guideline acknowledged these markers solely for classification without deriving specific therapeutic mandates. Furthermore, the models displayed a distinct regulatory and geographic bias, frequently importing nonlocal standards into the German context. This included framing US-approved agents (belinostat and pralatrexate) or Japanese-approved drugs (darinaparsin) as the “current standard of care.” Additionally, outputs often failed to reflect clinical nuances, often predicting absolute paradigm shifts, such as brentuximab vedotin with cyclophosphamide, doxorubicin, and prednisone (BV-CHP) unequivocally replacing cyclophosphamide, doxorubicin, vincristine, prednisone (CHOP) or the immediate introduction of chimeric antigen receptor T-cell (CAR-T) and programmed death-ligand 1 (PD-L1) inhibitors, whereas the human committee maintained a more conservative framework of alternative options (“OR” decisions) and reserved experimental modalities for specific trial settings.

### Qualitative Error and Source Analysis

**Figure 1.**
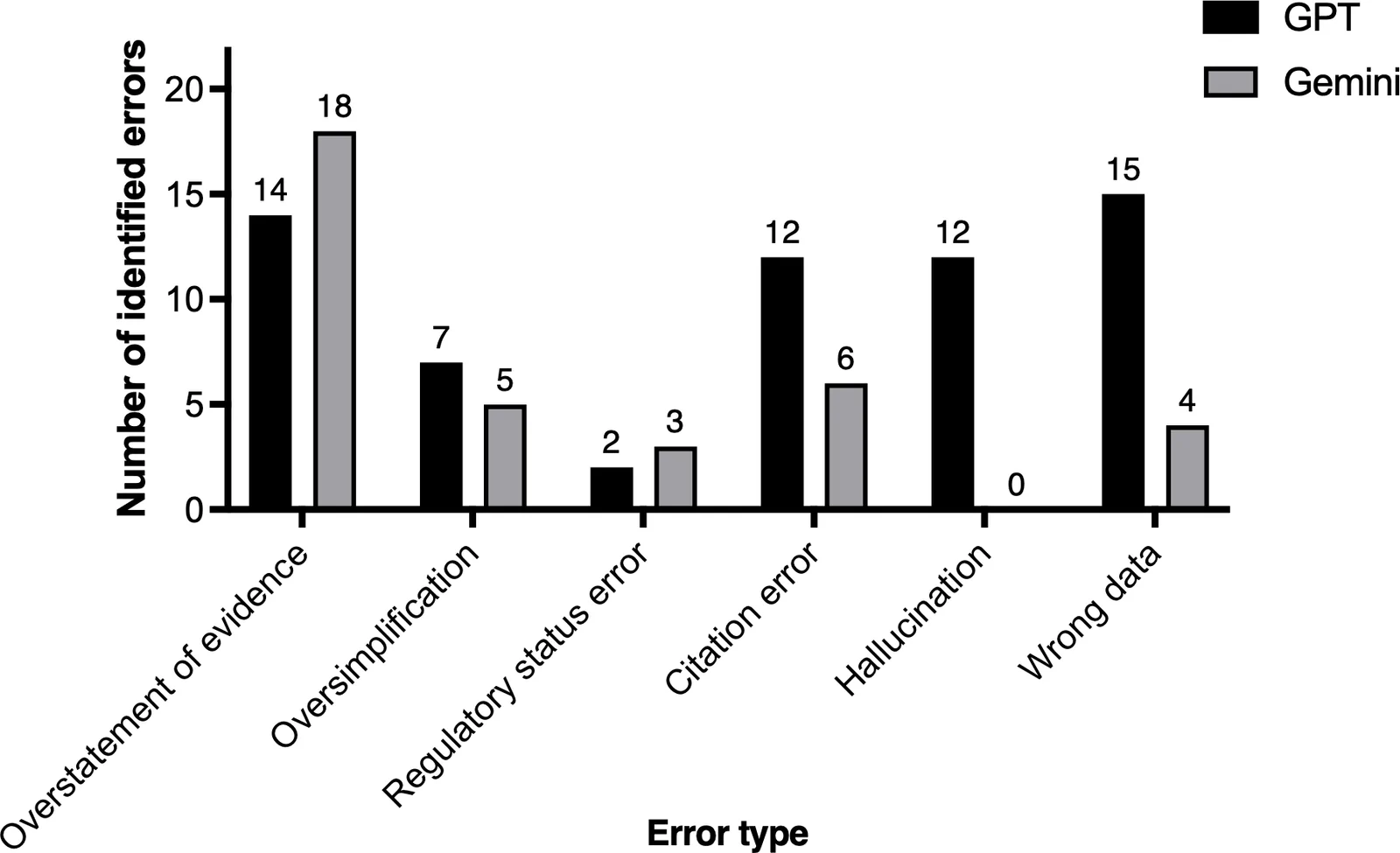
Quantification of error types in large language model–generated guideline drafts. Bar chart displaying the absolute frequency of specific error categories identified within the generated peripheral T-cell lymphoma guideline update proposals. Errors were classified through qualitative manual review into 6 distinct domains.

Moving from predictive accuracy to textual fidelity, we found that while the drafts appeared superficially plausible, they contained a substantial number of factual errors and inaccuracies (Figure 1 and Table S5 in Multimedia Appendix 3). Overall, GPT produced a higher frequency of inaccuracies than Gemini (62 vs 36 total errors). Both models shared a tendency toward “overstatement of evidence,” frequently characterizing modest therapeutic effects as “practice-changing.” Gemini was particularly prone to such premature conclusions, often elevating early-phase data to standard-of-care status without adequate evidence. In contrast, GPT exhibited a high rate of citation errors, consistently inverting first and last authors. More critically, GPT extracted the wrong study data in 15 instances. We also identified 12 completely fabricated studies (“hallucinations”) generated by GPT.

In terms of the overall accuracy of explicitly stated end points within the PTCL update drafts, Gemini demonstrated higher accuracy in reporting specific study end points (20/23, 87% correct) compared with GPT (16/26, 61.5% correct). The performance of GPT declined with document length: while accuracy was relatively high in the first half of the document (11/13, 84.6%), it decreased to 38.5% (5/13) in the second half.

Further bibliometric analysis showed that the models used distinct sourcing strategies (Table 2). While GPT prioritized high-impact primary sources (median impact factor 17.7 vs 5.5), Gemini generated a broader reference list (62 vs 26 unique sources) that relied heavily on secondary literature (23 reviews, editorials, or other guidelines) and official regulatory documentation. Approximately 20% of the citations generated by both models consisted of media news reports (GPT 5/26, 19.2%; Gemini 14/62, 22.6%). Citation concordance with the human committee was low: of the 26 new publications added to the 2025 guideline revision, Gemini identified only 3.8% (1/26) and GPT identified only 11.5% (3/26).

**Table 2.** Bibliometric analysis of sources cited by models in the proposed peripheral T-cell lymphoma Onkopedia updates.

Metrics	GPT	Gemini
Unique sources, n (%)^a^		
Primary (original research articles)	11 (42.3)	10 (16.1)
Secondary (reviews, editorials, and guidelines)	6 (23.1)	23 (37.1)
Conference abstracts	4 (15.4)	6 (9.7)
News reports	5 (19.2)	14 (22.6)
Official web pages^b^	0 (0)	9 (14.5)
Journal impact factor (2024), median (IQR)	17.7 (5.3-43.4)	5.5 (3.0-18.1)
Overlap with new guideline references (n=26), n (%)	3 (11.5)	1 (3.8)

a GPT: n=26; Gemini: n=62.

b Including those of ClinicalTrials.gov, European Medicines Agency, and the US Food and Drug Administration.

### Fidelity of Isolated Evidence Extraction

To determine whether the citation errors and hallucinations observed in the predictive workflow stemmed from an inability to accurately retrieve primary data, we isolated the extraction task and tested it across 80 pivotal studies. By benchmarking open-source and reasoning models of varying sizes (ranging from 20B to 671B parameters) alongside the frontier models, we further aimed to evaluate the impact of model scale and grounding with internet search on extraction fidelity.

**Figure 2.**
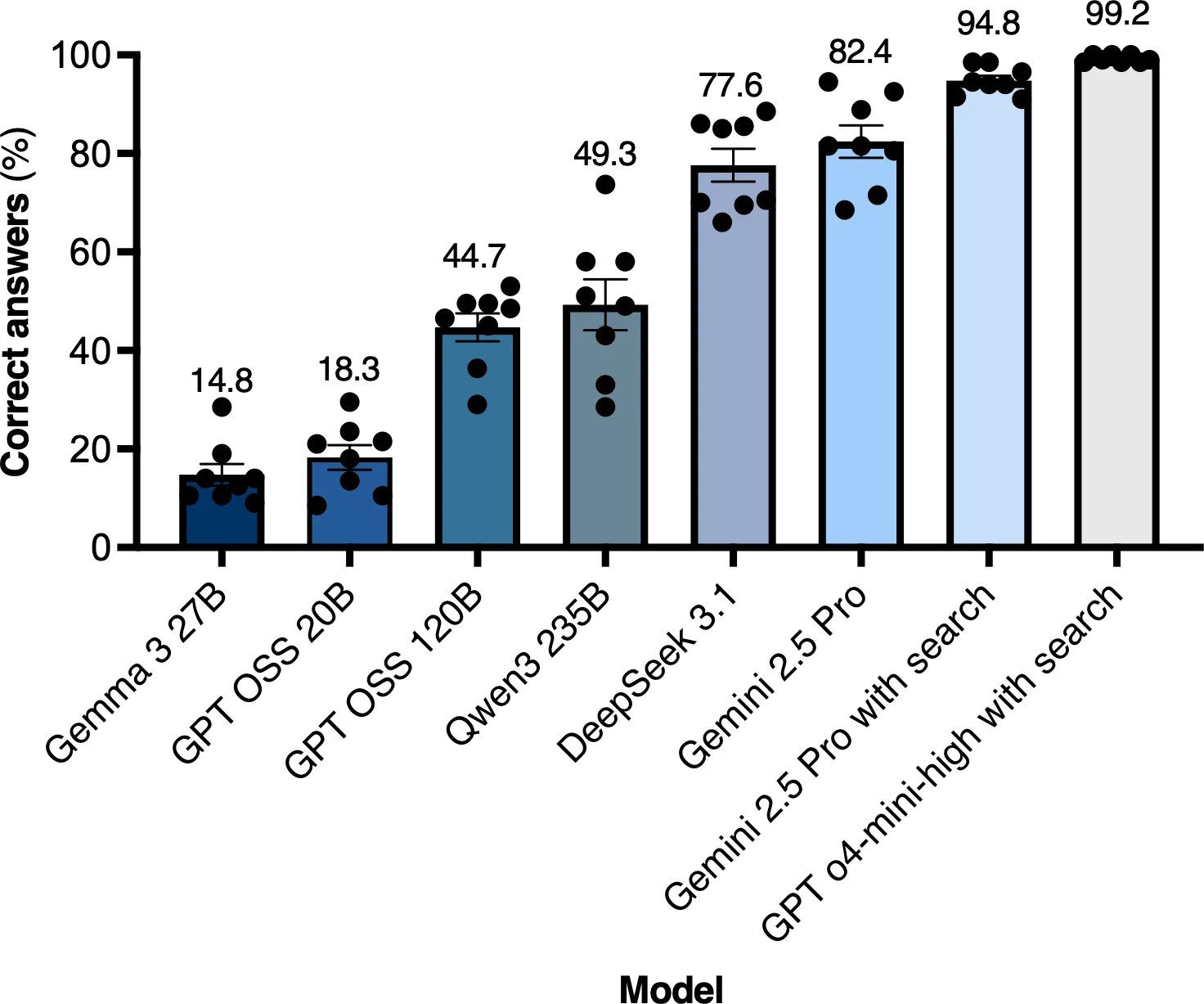
Benchmarking of clinical trial data extraction fidelity across large language models. Bars show the mean percentage of correctly extracted fields across 80 pivotal studies (8 tumor entities×10 studies per entity), using a 0/0.5/1 scoring rubric per extracted variable (10 variables per study; partial credit for prespecified minor deviations). “With search” means access to internet. Black dots denote entity-level mean accuracy (one dot per tumor entity). Error bars represent the SE of the mean across tumor entities.

Results revealed a performance hierarchy governed by model scale and connectivity (Figure 2). While smaller open-source models (Gemma 3 27B and GPT OSS 20B) performed poorly (<20%), accuracy improved substantially with parameter count (GPT OSS 120B and Qwen3 235B) and reached a moderately high plateau with reasoning models such as DeepSeek 3.1 using 671B parameters (77.6%). Although proprietary frontier models significantly outperformed open-source models overall (*P*<.001), this gap narrowed at the top tier: DeepSeek 3.1 was not significantly worse than the baseline proprietary model without search grounding, Gemini 2.5 Pro (*P*=.08). For frontier models, external grounding was associated with higher precision; enabling internet search capabilities for Gemini increased accuracy from 82.3% to 94.8% (*P*=.008). GPT achieved very high correct retrieval rates (99.2%), demonstrating 100% accuracy across several categories (Table S6 in Multimedia Appendix 3).

### LLMs as Automated Quality Assurance

**Figure 3.**
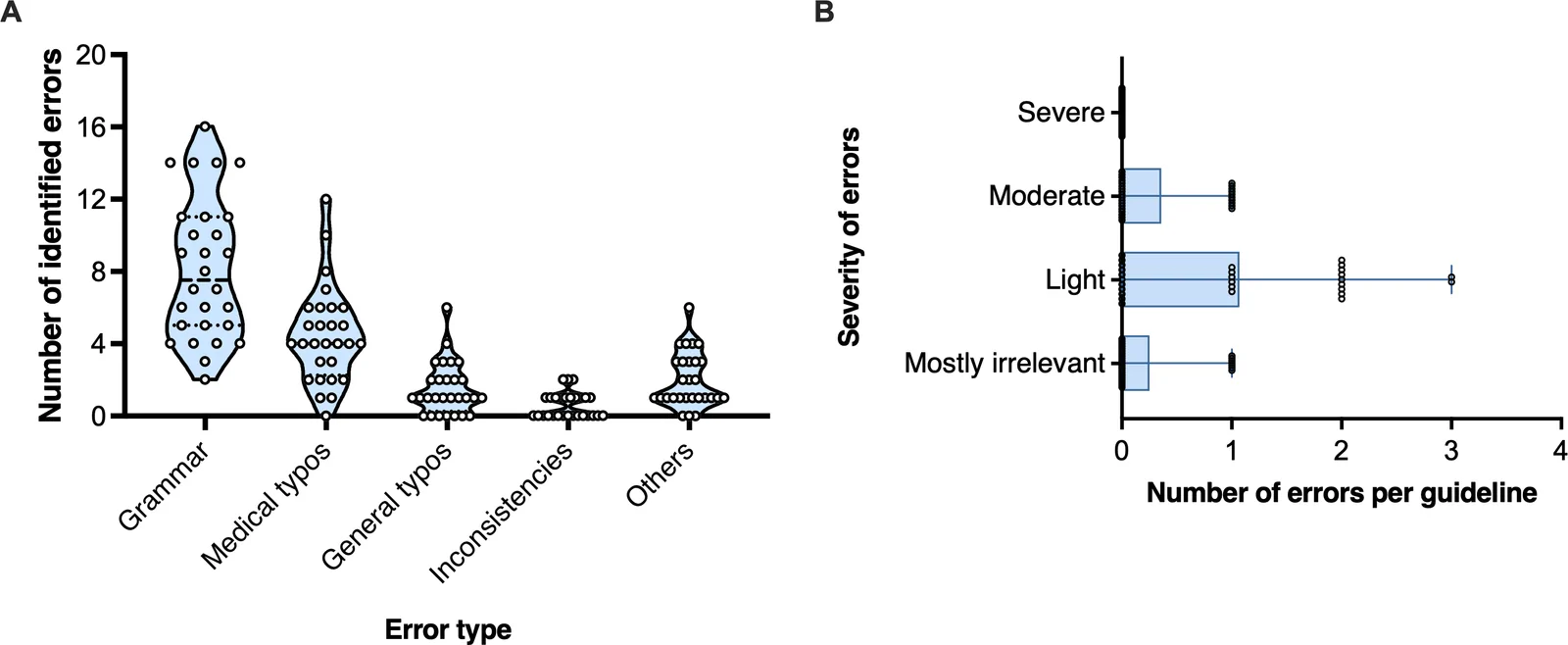
Density and severity of errors detected by the automated large language model quality assurance workflow. (A) Violin plot illustrating the distribution of linguistic and formal errors identified per guideline document (n=28). Dashed lines represent the median and quartiles. (B) Frequency of content-related errors per document, stratified by clinical severity. The bar represents the mean, and lines indicate the minimum and maximum values. Dots show individual values per guideline.

Having demonstrated that LLMs are currently incapable of independently performing guideline updates at sufficient quality, we sought to identify alternative use cases within the CPG workflow. Building on the hypothesis that LLMs currently function more effectively as critical reviewers than as primary authors [27], we deployed a stacked Gemini and GPT workflow to audit 28 recently updated Onkopedia guidelines. This automated review process revealed a persistent background of formal imperfections (Figure 3A), identifying a median of 16.5 linguistic errors per document. These were predominantly grammatical errors and, more relevantly, typos of medical words, such as the misspelling of specific chemotherapy protocols or diseases (refer to Table S6 in Multimedia Appendix 3 for the full list of errors).

More critically, the LLMs detected content-related inconsistencies in most guidelines (Figure 3B). While the models did not identify any severe errors (defined as those with immediate and direct danger to patient life), they flagged multiple moderate errors with significant potential to confuse clinical decision-making (Table S8 in Multimedia Appendix 3). Notable detections included mathematical errors in prognostic scoring formulas (eg, errors in the Sokal score for chronic myeloid leukemia that could potentially misclassify a high-risk patient as low-risk, altering first-line treatment), imprecise definitions of staging thresholds, and inconsistent definitions of resistance mechanisms. The models also identified conceptual conflations between distinct biomarker units and flagged outdated regulatory descriptions, such as correctly identifying a maintenance therapy described as “off-label” in the text despite a recent label expansion. Across the 28 guidelines (mean length 21,441 tokens), the computational cost for the dual-model workflow was less than US $0.50 per document. This workflow required only 10 to 15 minutes of manual verification of the generated flags, which represents a relevant reduction in workload when compared with the substantial time standard human-only peer review demands for reading and auditing a document of such length.

## Discussion

### Principal Findings

This study provides a comprehensive evaluation of using LLMs for clinical guidelines in oncology. To our knowledge, this analysis is among the first to evaluate multiple LLM roles across guideline update prediction, trial extraction, and quality control within an oncology guideline program. Across these tasks, we identified a dichotomy in current model capabilities: while models demonstrate high accuracy in bounded, verifiable microtasks such as data extraction and error detection, they remain unreliable as autonomous authors for complex, multisource guideline synthesis.

### The Case Study

A key strength of this study design was the use of the PTCL guideline update as a prospective experiment. Unlike standard retrospective evaluations, where models are tested on guidelines that may already exist within their training data, our approach tasked the models with predicting an update before its publication. This setup reduced the likelihood of training data overlap and forced the models to rely exclusively on de novo reasoning and information retrieval. The resulting moderate predictive accuracy (36.7%‐40%) therefore represents a pragmatic estimate under our prompting and tool conditions. Crucially, while the underlying clinical trial data were already publicly available and likely accessible via autonomous web-search capabilities, the models still struggled to accurately construct the final guideline. This underscores that the bottleneck lies not in retrieving existing data but in the complex, context-specific synthesis required for guideline authoring. Although we cannot rule out that models occasionally retrieved and misapplied recommendations from other international guidelines (contributing to the observed regulatory bias), the highly specific structural requirements of the PTCL task required independent reasoning that the models could not reliably provide.

The analysis showed that model outputs often captured prominent signals, identifying landmark trials and new approvals but did not reliably reproduce the nuanced, typically conservative reasoning applied in CPG maintenance. Specifically, we observed a recurring tendency toward premature clinical translation, where models anticipated that emerging molecular subtypes would immediately dictate risk-adapted treatment algorithms, whereas the actual guideline acknowledged these markers solely for classification. Similarly, models frequently predicted absolute paradigm shifts, such as a new agent unequivocally replacing an established standard, whereas human experts maintained a flexible framework of alternative options. This tendency to overstate evidence is directly linked to the models’ retrieval modes. For example, Gemini’s high reliance on non–peer-reviewed news reports (14/62, 22.6% of citations) demonstrates how general-purpose search algorithms fail to apply the rigorous biomedical filters required for reliable guideline maintenance. Furthermore, the models exhibited a regulatory and geographic bias, failing to localize global evidence to the specific health care context (eg, defaulting to US Food and Drug Administration [FDA] approvals rather than the European Medicines Agency [EMA]–centric framework of Onkopedia).

### The Problem of “Attention Dilution”

A relevant technical finding of our study was the difference between the high performance in isolated evidence extraction and the frequent errors and hallucinations observed during guideline drafting. In our extraction benchmark, frontier models such as GPT o4-mini-high achieved 99.2% accuracy when retrieving data from single studies. However, when the same models were tasked with synthesizing multiple studies into a cohesive guideline section, accuracy dropped, and hallucinations increased. We also observed a position-dependent accuracy decline, with incorrect study endpoints and hallucinations increasing toward the end of the document.

While the established “lost-in-the-middle” phenomenon describes how models fail to retrieve information from the center of long input contexts [28], our findings highlight a related but distinct degradation during output generation. We propose the informal descriptive label “attention dilution” here to characterize how models struggle to maintain focus over long generative tasks, although other technical limitations such as instruction drift, context window saturation, or output token constraints could equally contribute to this performance decay. Regardless of the exact mechanism, the complex task of synthesizing heterogeneous sources, which includes balancing primary trials against reviews, reconciling conflicting data, and maintaining narrative coherence, leads to a reduction in overall accuracy. Consequently, the models generated plausible but incorrect details when reconciling sources. These findings contextualize emerging evidence on LLM-assisted systematic reviews. While LLMs show promise for literature screening, data extraction accuracy has repeatedly been shown to be inadequate across complex fields [29-34].

### LLMs as Automated Quality Assurance

In contrast to their limitations as primary authors, the models demonstrated utility as automated quality assurance agents. The stacked Gemini and GPT workflow successfully identified a median of 16.5 formal errors per document and acted as a highly cost-effective safety mechanism by flagging content-related inconsistencies that had escaped human review. The detection of mathematically invalid scoring formulas, incorrect TNM definitions, and off-label discrepancies in recently published guidelines suggests that LLMs are currently more effective as critics than as creators in medical contexts. Instead of using LLMs to generate drafts requiring extensive human verification, domain experts should author the core recommendations and use LLMs as an audit layer to flag internal inconsistencies, reasoning errors, and minor inaccuracies with potential clinical relevance [27,35]. Of note, this error detection capability required explicit prompting. In the absence of a dedicated “auditor” instruction, such as during the update generation task, models tended to unquestioningly propagate antecedent errors, exemplified by the replication of an invalid clinical trial identifier (NCT0308190) directly from a primary source publication [36].

### Limitations

Several limitations frame the interpretation of these results. First, the predictive component focused on a single, highly complex entity (PTCL) within a specific national context. While PTCL provided a rigorous test case due to its nuanced treatment pathways, the findings cannot be generalized across oncology. For more common tumor types with large, rapidly evolving evidence bases (eg, breast or non–small cell lung cancer), the observed phenomenon of “attention dilution” would likely be exacerbated. In such information-dense environments, the challenge of correctly balancing conflicting primary trials against an even larger volume of secondary literature would probably lead to higher hallucination rates and an even stronger reliance on existing international guideline syntheses. Our analysis approach of using actively generated end points as the denominator could also theoretically penalize models attempting a more comprehensive, data-dense synthesis. In our study, the absolute difference in generated end points was negligible (GPT: 26; Gemini: 23); future evaluations, however, should interpret these percentages alongside absolute counts to account for potential denominator bias.

Second, the study relied on a specific snapshot of model capabilities (August and December 2025), meaning these performance benchmarks are transient given the rapid pace of LLM development. Additionally, the use of proprietary “deep-research” modes introduces a confounder because platform-level differences in hidden retrieval algorithms cannot be fully controlled. Third, we used optimized but static prompts; we did not use an advanced automated prompt optimization framework or fine-tuning, which could potentially enhance performance. Specifically, the imposition of a rigid 2-stage workflow may have constrained the autonomous agentic capabilities of the deep-research modes by inhibiting the recursive search loops required to uncover less accessible evidence. Fourth, the PTCL guideline underwent a substantial restructuring between 2021 and 2025, with large portions of the text being rewritten, thereby presenting a significantly more complex prediction challenge than a standard incremental update. Furthermore, an inherent structural bias was present in the primary evaluation: the human evaluators were not blinded to the specific model, and the 30-point ground-truth rubric was created by the same authors who subsequently scored the outputs. To mitigate the resulting risk of confirmation bias, an independent, model-blinded rescoring by a third author was conducted. Fifth, the use of the human-authored guideline as the reference standard carries inherent uncertainty, as it assumes the human committee captured every relevant update perfectly; it is theoretically possible that some “false-positive” LLM suggestions were valid evidence points overlooked by the human authors. Sixth, regarding the automated quality assurance task, we verified all errors flagged by the models but did not conduct an exhaustive human reaudit of the 28 guidelines to establish a ground-truth baseline. Consequently, the true false-negative rate (ie, errors missed by the LLMs) remains unknown, meaning our findings demonstrate high precision but cannot quantify recall. Finally, our stacked quality assurance workflow was designed as an exploratory ensemble approach to maximize error detection. However, this sequential design conflates individual model performance, prevents the causal attribution of error detection to either model independently, and does not account for potential anchoring bias.

### Future Directions

Future research should move beyond single-prompt interactions toward multiagent systems that simulate the structure of a guideline committee. Such an approach could assign distinct roles to different model instances, for example, a “screener” agent to retrieve literature, a “statistician” agent to extract data, a “skeptic” agent to challenge evidence strength, and a “synthesizer” agent to draft text. Systematically evaluating these role-based architectures could mitigate the attention dilution observed in single-model synthesis. Additionally, localization layers can be added to adapt global evidence syntheses to specific national regulatory and reimbursement landscapes. Furthermore, prospective prediction studies in common tumor guidelines (eg, breast or lung cancer) are required for external validation. So far, it remains indeterminate whether denser training data in these entities will improve performance or whether larger literature volumes will instead exacerbate “attention dilution.” In addition, the 2025 PTCL revision was a near-complete restructuring synthesizing 4 years of new evidence. Evaluating models on such a substantial overhaul may underestimate their utility for continuous “living guideline” maintenance. As living guidelines rely on frequent, narrowly scoped updates triggered by individual studies and frontier models performed well at bounded extraction, they may prove effective for these incremental amendments. Prospective evaluation in a living guideline setting is therefore an important next step.

Finally, prospective implementation studies should evaluate whether integrating LLM-based quality assurance loops into the actual content management systems of guideline bodies reduces the time to publication or the rate of postpublication errata [37]. Such efficiency gains are essential, given that the conventional update process, as evidenced by the PTCL timeline from expert nomination in February 2024 to final publication in October 2025, currently consumes up to 2 years of expert resources. On the basis of our findings, the most pragmatic near-term application is a human-led, AI-augmented workflow: human experts must retain responsibility for critical appraisal, recommendation strength, and local regulatory context, while LLMs can effectively support evidence structuring, ongoing surveillance, and prepublication auditing.

### Conclusions

This case study using German oncology guidelines suggests that LLMs currently lack the reasoning stability for autonomous guideline authoring, as multisource synthesis remains prone to factual errors and hallucinations over long contexts. Nevertheless, the evaluated models demonstrated high accuracy in bounded, verifiable tasks, successfully performing trial data extraction and systematic quality assurance capable of detecting formal errors and clinically relevant inconsistencies in published guidelines.

## Supplementary material

10.2196/93239 Multimedia Appendix 1 Timeline of guideline update prediction task.

10.2196/93239 Multimedia Appendix 2 Prompts and outputs.

10.2196/93239 Multimedia Appendix 3 Supplementary tables S1-S8.

## Abbreviations

BV-CHP: brentuximab vedotin with cyclophosphamide, doxorubicin, and prednisone
CAR-T: chimeric antigen receptor T-cell
CHOP: cyclophosphamide, doxorubicin, vincristine, prednisone
CPG: clinical practice guideline
EMA: European Medicines Agency
FDA: Food and Drug Administration
ICC: International Consensus Classification
LLM: large language model
PD-L1: programmed death-ligand 1
PET/CT: positron emission tomography/computed tomography
PTCL: peripheral T-cell lymphoma
*WHO-HAEM5*: *5th edition of World Health Organization Classification of Haematolymphoid Tumours*
