# Supplementary material for: Large Language Models for Oncology Guideline Maintenance: Prospective Case Study
Source: JMIR AI. 2026 Aug 20;5:e93239. doi: 10.2196/93239 (PMC13492301; doi:10.2196/93239)
Supplement: Supplementary file 1 [file ai-v5-e93239-s001.pdf]

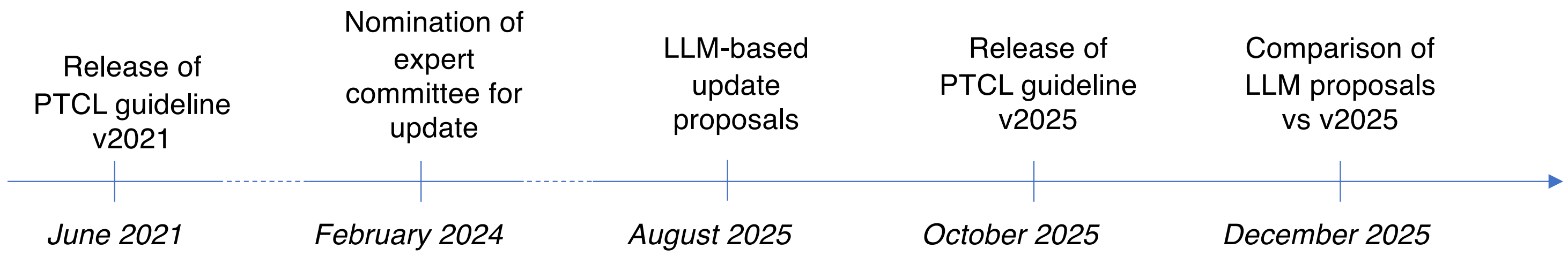
Release of PTCL guideline v2021
June 2021
Nomination of expert committee for update
February 2024
LLM-based update proposals
August 2025
Release of PTCL guideline v2025
October 2025
Comparison of LLM proposals vs v2025
December 2025
