# Supplementary material for: Large Language Models for Oncology Guideline Maintenance: Prospective Case Study
Source: JMIR AI. 2026 Aug 20;5:e93239. doi: 10.2196/93239 (PMC13492301; doi:10.2196/93239)
Supplement: Supplementary file 2 [file ai-v5-e93239-s002.docx]

**Content**

[1. Prompt for Onkopedia update (German)	2](#_Toc221055702)

[2. Prompts for Onkopedia update (English)	6](#_Toc221055703)

[3. Prompt for clinical trial evidence extraction	9](#_Toc221055704)

[4. Prompt for error detection in Onkopedia guidelines (German)	10](#_Toc221055705)

[5. Prompt for error detection in Onkopedia guidelines (English)	13](#_Toc221055706)

[6. GPT o4-mini-high output (German)	15](#_Toc221055707)

[7. GPT o4-mini-high output (English)	32](#_Toc221055708)

[8. Gemini 2.5 Pro output (German)	53](#_Toc221055709)

[9. Gemini 2.5 Pro output (English)	76](#_Toc221055710)

* Translations from German to English have been automatically performed using DeepL.

# 1. Prompt for Onkopedia update (German)

**Prompt 1 für Gemini 2.5 Pro**

„Generiere einen ausführlichen Prompt für ein Deep Research LLM, um die Onkopedia-Leitlinie „Periphere T-Zell-Lymphome“ (Stand Juni 2021) auf den jetzigen Stand zu aktualisieren“

**Prompt 2 für Gemini/GPT Deep Research:**

**„Aktualisierung der Onkopedia-Leitlinie "Periphere T-Zell-Lymphome"**

**Rolle und Zielsetzung:**

Sie agieren als hochspezialisierter klinischer Forschungsassistent mit tiefgehender Expertise in der Hämatologie, insbesondere bei aggressiven Non-Hodgkin-Lymphomen mit Fokus auf T-Zell-Neoplasien. Ihr Hauptziel ist die systematische Identifizierung, Analyse und Synthese aller relevanten, praxisverändernden klinischen Daten, Studienergebnisse, diagnostischen Neuerungen und neuen Zulassungen, die seit der letzten Überarbeitung der Onkopedia-Leitlinie "Periphere T-Zell-Lymphome" (Stand Juni 2021) veröffentlicht wurden. Ihre Recherche muss die enorme Heterogenität der PTCL-Subtypen berücksichtigen und die Ergebnisse entsprechend differenzieren.

**Fokus und Zeitrahmen:**

- **Zeitrahmen der Recherche:** Berücksichtigen Sie alle relevanten Veröffentlichungen und Präsentationen vom **1. Juni 2021 bis zum heutigen Tag**.
- **Fokus:** Konzentrieren Sie sich auf Daten, die das Potenzial haben, die klinische Praxis zu verändern. Priorisieren Sie dabei:
  1. Ergebnisse aus randomisierten Studien (selten bei PTCL, daher höchste Priorität).
  2. Zulassungserweiterungen oder neue Zulassungen von Medikamenten durch EMA und FDA.
  3. Wichtige Aktualisierungen in anderen international führenden Leitlinien (insb. NCCN, ESMO).
  4. Relevante, prospektive Phase-II-Studien mit hohem Ansprechen oder langer Wirkdauer, insbesondere für neue Substanzklassen oder Kombinationen.
  5. Langzeit-Follow-up-Daten etablierter Studien (z.B. ECHELON-2), die das Gesamtüberleben (OS) beeinflussen.
  6. Neue Erkenntnisse zur molekularen Klassifikation, die eine therapeutische Relevanz haben.

**Primäre Forschungsfragen nach Guideline-Sektionen:**

Bitte strukturieren Sie Ihre Recherche und Ergebnisse entlang der folgenden, für PTCL relevanten Bereiche:

**1. Diagnostik, Pathologie und Molekulare Klassifikation:**

- **Klassifikation:** Gab es relevante Änderungen in der **5. Auflage der WHO-Klassifikation** oder der **International Consensus Classification (ICC)**, die die Definition oder Abgrenzung von PTCL-Subtypen betreffen?
- **Molekulare Diagnostik:** Gibt es neue, validierte molekulare Marker (z.B. aus Genexpressions- oder Mutationsanalysen), die zur Standard-Risikostratifizierung oder zur Therapieentscheidung bei den Hauptsubtypen (PTCL-NOS, AITL, ALCL) herangezogen werden sollten?
- **CD30-Expression:** Hat sich die Methodik oder der Schwellenwert zur Bestimmung der CD30-Expression geändert, der für den Einsatz von Brentuximab Vedotin relevant ist?

**2. Erstlinientherapie:**

- **CD30-positive PTCL (insb. sALCL):** Gibt es Langzeit-Follow-up-Daten der **ECHELON-2 Studie**(Brentuximab Vedotin + CHP)? Bestätigen diese den Vorteil im Gesamtüberleben gegenüber CHOP und untermauern BV-CHP als Standard für alle CD30+ PTCL?
- **CD30-negative/niedrige PTCL (insb. PTCL-NOS, AITL):** Haben sich neue Therapiestandards etabliert, die über CHO(E)P hinausgehen? Gibt es vielversprechende Phase-III- oder randomisierte Phase-II-Studienergebnisse zu Kombinationen von CHOP mit neuen Substanzen (z.B. Hypomethylierungsagentien, Antikörper)?
- **Konsolidierung in der Erstremission:** Gibt es neue Daten, die die Rolle der **autologen oder allogenen Stammzelltransplantation** als Konsolidierung nach der Erstlinientherapie klarer definieren? Gibt es subtyp-spezifische Empfehlungen?

**3. Therapie des rezidivierten/refraktären (R/R) PTCL:**

- **Neue Zulassungen:** Wurden seit Juni 2021 neue Medikamente oder Kombinationstherapien von EMA oder FDA für R/R PTCL zugelassen?
- **Gezielte Therapien & Kombinationen:** Welche neuen Daten gibt es zu:
  - **Brentuximab Vedotin:** Gibt es neue Kombinationen oder erweiterte Anwendungsgebiete im R/R-Setting?
  - **HDAC-Inhibitoren** (z.B. Romidepsin): Gibt es neue Kombinationsdaten?
  - **Hypomethylierungsagentien** (z.B. Azacitidin): Gibt es neue Daten, insbesondere bei AITL?
  - **JAK-Inhibitoren:** Gibt es neue Erkenntnisse zum Einsatz bei PTCL mit Mutationen im JAK/STAT-Signalweg?
  - **PI3K-Inhibitoren** (z.B. Duvelisib): Wie ist der aktuelle Stellenwert nach initialen vielversprechenden, aber auch toxischen Ergebnissen?
- **Allogene Stammzelltransplantation:** Gibt es neue Erkenntnisse zu Konditionierungsregimen, Spenderwahl oder Post-Transplantations-Strategien im R/R-Setting?
- **Zelluläre Therapien:** Gibt es relevante Fortschritte oder frühe klinische Daten zu **CAR-T-Zell-Therapien** für T-Zell-Lymphome (z.B. CD5- oder CD7-gerichtete CARs)?

**4. Spezifische und seltene Subtypen:**

- **Angioimmunoblastisches T-Zell-Lymphom (AITL):** Welche neuen, auf die spezifische Biologie abzielenden Therapien (z.B. Azacitidin-basierte Schemata) haben sich in Studien bewährt?
- **ALK-negatives Anaplastisch-großzelliges Lymphom (ALK- ALCL):** Bestätigen Langzeitdaten den Stellenwert von BV-CHP als Erstlinientherapie?
- **Extranodales NK/T-Zell-Lymphom, nasaler Typ (ENKTL):** Gibt es Updates zu Asparaginase-haltigen Schemata oder neue Daten zum Einsatz von Immun-Checkpoint-Inhibitoren?
- **Hepatosplenisches T-Zell-Lymphom (HSTL):** Gibt es neue Erkenntnisse aus Registerstudien oder Fallserien, die Therapieempfehlungen für diese ultra-seltene, aggressive Entität leiten könnten?

**Quellenanforderungen:**

Stützen Sie Ihre Recherche auf folgende hochrangige Quellen:

- **Journals:** Blood, Journal of Clinical Oncology (JCO), The Lancet Haematology, The Lancet Oncology, Leukemia, Haematologica.
- **Konferenzen:** ASH (American Society of Hematology), EHA (European Hematology Association), ASCO, ICML (International Conference on Malignant Lymphoma, Lugano).
- **Leitlinien:** NCCN, ESMO.
- **Zulassungsbehörden:** EMA (CHMP), FDA.

**Format der Ausgabe:**

Bitte bereiten Sie die Ergebnisse in einem strukturierten Markdown-Format auf. Fassen Sie für jeden relevanten Befund die folgenden Punkte zusammen:

Generated markdown

\### Sektion: [z.B. Erstlinientherapie CD30-positiver PTCL]

**Kernaussage:**

[Fassen Sie die zentrale neue Erkenntnis in 1-2 Sätzen zusammen. z.B. "Das 5-Jahres-Follow-up der ECHELON-2 Studie bestätigt einen anhaltenden und signifikanten Vorteil im Gesamtüberleben für Brentuximab Vedotin + CHP gegenüber CHOP."]

**Studiendetails/Quelle:**

*	**Studie:** [z.B. ECHELON-2 (5-Year Update)]

*	**Publikation/Präsentation:** [z.B. Horwitz S. et al., JCO, 2022]

*	**Patientenpopulation:** [z.B. n=452, Patienten mit neu diagnostiziertem CD30-positivem PTCL]

*	**Endpunkte:** [z.B. Primärer Endpunkt: PFS. Wichtiger sekundärer Endpunkt: OS]

**Evidenzlevel/Empfehlungsgrad (geschätzt):**

[z.B. Evidenzlevel Ia, Empfehlungsgrad A]

**Implikation für die Onkopedia-Leitlinie:**

[Beschreiben Sie die konkrete Auswirkung. z.B. "Die Empfehlung für BV-CHP als Standard-Erstlinientherapie für CD30-positive PTCL wird durch die Langzeitdaten weiter zementiert und sollte als bevorzugte Option klar formuliert werden."]“

# 2. Prompts for Onkopedia update (English)

**Prompt 1 for Gemini 2.5 Pro**

“Generate a detailed prompt for a Deep Research LLM to update the Onkopedia guideline ‘Peripheral T-cell lymphomas’ (as of June 2021) to the current status.”

**Prompt 2 for Gemini/GPT Deep Research:**

**“Update the Onkopedia guideline ‘Peripheral T-cell lymphomas’.”**

**Role and objective:**

You act as a highly specialized clinical research assistant with in-depth expertise in hematology, particularly aggressive non-Hodgkin's lymphomas with a focus on T-cell neoplasms.

Your main objective is the systematic identification, analysis, and synthesis of all relevant, practice-changing clinical data, study results, diagnostic innovations, and new approvals that have been published since the last revision of the Onkopedia guideline “Peripheral T-cell lymphomas” (as of June 2021). Your research must take into account the enormous heterogeneity of PTCL subtypes and differentiate the results accordingly.

**Focus and time frame:**

- **Time frame of the research:** Consider all relevant publications and presentations from **June 1, 2021, to the present day**.
- **Focus:** Concentrate on data that has the potential to change clinical practice. Prioritize the following:
  1. Results from randomized studies (rare in PTCL, therefore highest priority).
  2. Label extensions or new drug approvals by the EMA and FDA.
  3. Important updates in other leading international guidelines (especially NCCN, ESMO).
  4. Relevant, prospective phase II studies with high response rates or long duration of action, especially for new drug classes or combinations.
  5. Long-term follow-up data from established studies (e.g., ECHELON-2) that influence overall survival (OS).
  6. New findings on molecular classification that have therapeutic relevance.

**Primary research questions by guideline section:**

Please structure your research and results according to the following areas relevant to PTCL:

**1. Diagnostics, pathology, and molecular classification:**

- **Classification:** Were there any relevant changes in the **5th edition of the WHO classification** or the **International Consensus Classification (ICC)** that affect the definition or delineation of PTCL subtypes?
- **Molecular diagnostics:** Are there new, validated molecular markers (e.g., from gene expression or mutation analyses) that should be used for standard risk stratification or treatment decisions for the main subtypes (PTCL-NOS, AITL, ALCL)?
- **CD30 expression:** Has the methodology or threshold for determining CD30 expression, which is relevant for the use of brentuximab vedotin, changed?

**2. First-line therapy:**

- **CD30-positive PTCL (especially sALCL):** Are there long-term follow-up data from the **ECHELON-2 study**(brentuximab vedotin + CHP)? Do these confirm the advantage in overall survival compared to CHOP and substantiate BV-CHP as the standard for all CD30+ PTCL?
- **CD30-negative/low PTCL (especially PTCL-NOS, AITL):** Have new treatment standards been established that go beyond CHO(E)P? Are there any promising phase III or randomized phase II study results on combinations of CHOP with new substances (e.g., hypomethylation agents, antibodies)?
- **Consolidation in first remission:** Is there new data that more clearly defines the role of **autologous or allogeneic stem cell transplantation** as consolidation after first-line therapy? Are there subtype-specific recommendations?

**3. Treatment of relapsed/refractory (R/R) PTCL:**

- **New approvals:** Have any new drugs or combination therapies been approved by the EMA or FDA for R/R PTCL since June 2021?
- **Targeted therapies & combinations:** What new data is available on:
  - **Brentuximab vedotin:** Are there any new combinations or expanded indications in the R/R setting?
  - **HDAC inhibitors** (e.g., romidepsin): Is there any new combination data?
  - **Hypomethylating agents** (e.g., azacitidine): Is there any new data, particularly for AITL?
  - **JAK inhibitors:** Are there any new findings on their use in PTCL with mutations in the JAK/STAT signaling pathway?
  - **PI3K inhibitors** (e.g., duvelisib): What is their current status after initial promising but also toxic results?
- **Allogeneic stem cell transplantation:** Are there any new findings on conditioning regimens, donor selection, or post-transplantation strategies in the R/R setting?
- **Cellular therapies:** Are there any relevant advances or early clinical data on **CAR-T cell therapies** for T-cell lymphomas (e.g., CD5- or CD7-targeted CARs)?

**4. Specific and rare subtypes:**

- **Angioimmunoblastic T-cell lymphoma (AITL):** Which new therapies targeting the specific biology (e.g., azacitidine-based regimens) have proven effective in studies?
- **ALK-negative anaplastic large cell lymphoma (ALK-ALCL):** Do long-term data confirm the importance of BV-CHP as first-line therapy?
- **Extranodal NK/T-cell lymphoma, nasal type (ENKTL):** Are there any updates on asparaginase-containing regimens or new data on the use of immune checkpoint inhibitors?
- **Hepatosplenic T-cell lymphoma (HSTL):** Are there any new findings from registry studies or case series that could guide treatment recommendations for this ultra-rare, aggressive entity?

**Source requirements:**

Base your research on the following high-quality sources:

- **Journals:** Blood, Journal of Clinical Oncology (JCO), The Lancet Haematology, The Lancet Oncology, Leukemia, Haematologica.
- **Conferences:** ASH (American Society of Hematology), EHA (European Hematology Association), ASCO, ICML (International Conference on Malignant Lymphoma, Lugano).
- **Guidelines:** NCCN, ESMO.
- **Regulatory authorities:** EMA (CHMP), FDA.

**Format of the output:**

Please prepare the results in a structured Markdown format. Summarize the following points for each relevant finding:

Generated markdown

\### Section: [e.g., First-line therapy for CD30-positive PTCL]

**Key message:**

[Summarize the key new finding in 1-2 sentences. e.g., “The 5-year follow-up of the ECHELON-2 study confirms a sustained and significant overall survival benefit for brentuximab vedotin + CHP versus CHOP.”]

**Study details/source:**

* **Study:** [e.g., ECHELON-2 (5-Year Update)]

* **Publication/presentation:** [e.g., Horwitz S. et al., JCO, 2022]

* **Patient population:** [e.g., n=452, patients with newly diagnosed CD30-positive PTCL]

* **Endpoints:** [e.g., primary endpoint: PFS. Important secondary endpoint: OS]

**Level of evidence/grade of recommendation (estimated):**

[e.g., level of evidence Ia, grade of recommendation A]

**Implication for the Onkopedia guideline:**

[Describe the specific impact. e.g., “The recommendation for BV-CHP as standard first-line therapy for CD30-positive PTCL is further cemented by the long-term data and should be clearly formulated as the preferred option.”]

# 3. Prompt for clinical trial evidence extraction

Role: You are an expert research assistant. Your task is to retrieve and summarize the details of a specific scientific hem/onc study. Use Search to ground your results based on the original paper on https://pubmed.ncbi.nlm.nih.gov (not the long-term follow-up) and the corresponding full text paper

If you do not know a requested item from memory, write “Unknown” (do NOT guess).

When reporting results, include the primary endpoint AND its key statistic (e.g., hazard ratio/odds ratio/risk difference, absolute values if known, and 95% CI if you recall it). Keep numbers concise.

Return your answer in this exact table, one line per cell:

Category (Details)

First Author

Journal

Year published

Disease covered

N (Participants)

Main Inclusion Criteria (2–3 most critical inclusion criteria)

Main Exclusion Criteria (2–3 most critical exclusion criteria)

Primary Endpoint & Statistic (name of primary endpoint + key effect size and 95% CI if recalled, otherwise “Unknown")

Main Results (1–2 sentences summarizing the primary outcome and key finding, with direction & magnitude; use “Unknown” if you cannot recall)

1-Sentence Impact (single sentence on study significance)

Study: ALFA-0701

# 4. Prompt for error detection in Onkopedia guidelines (German)

Rolle:

Du bist ein akribischer medizinischer Fachlektor und Onkologe mit Spezialisierung auf evidenzbasierte Leitlinien (z. B. DGHO/Onkopedia). Deine Aufgabe ist das gründliche Korrektorat und das inhaltliche Review eines Leitlinientextes.

Kontext:

Ich werde dir gleich die URL und den vollständigen Text einer neuen Onkopedia-Leitlinie geben. Der Text stammt oft aus einem CMS-Export oder Copy-Paste-Vorgang und enthält daher spezifische Formatierungsfehler sowie inhaltliche Inkonsistenzen. Wichtig: Das Dokument kann ein Datum in der Zukunft tragen (z. B. „Stand Oktober 2025“). Bitte bewerte zukünftige Datumsangaben oder Referenzen auf Kongresse in 2025 nicht als Fehler, sondern akzeptiere sie als gegebenen Stand des Dokuments.

Aufgabe:

Bitte analysiere den Text Schritt für Schritt und erstelle zwei getrennte Listen mit Fehlern. Sei extrem genau und pedantisch.

Liste 1: Rechtschreibung, Grammatik, Formatierung und Ausdruck

Achte auf:

•	Tippfehler: (z. B. „Lymhome“ statt „Lymphome“, fehlende Buchstaben).

•	Formatierungsfehler: (z. B. fehlende Leerzeichen bei ICD-Codes wie „ICD-10C15“, klebende Fußnotenzahlen im Fließtext, Copy-Paste-Reste wie „LINK“ im Text).

•	Grammatik: (Kongruenzfehler, Kasusfehler, falsche Bezüge).

•	Satzbau: (Satzbruchstücke, zerstörte Syntax durch Edits).

•	Ausdruck: (Falsche Übersetzungen aus dem Englischen, z. B. „Bewässerung“ statt „Hydrierung“/„Wässerung“, unpassende Begriffe).

Liste 2: Inhaltliche Fehler, Inkonsistenzen und Logik

Achte auf:

•	Widersprüche: (Steht im Fließtext etwas anderes als in der Tabelle? Widersprechen sich Therapiealgorithmus und Text?).

•	Akronyme: (Werden Abkürzungen falsch verwendet, z. B. „DDPG“ statt „DDGP“?).

•	Medizinische Plausibilität: (Stimmen Dosierungen grob? Sind Mutationsnamen korrekt? Sind Prozentzahlen mathematisch logisch?).

•	Referenzierung: (Werden Studien falsch zitiert oder Zahlen falsch übertragen?).

•	Veraltete Infos: (Werden Medikamente als „neu“ bezeichnet, die schon lange Standard sind, oder fehlen wichtige Warnhinweise?).

Output-Format:

Bitte gib die Fehler als übersichtliche Liste aus. Nenne jeweils:

1.	Den Ort (Kapitel/Abschnitt).

2.	Den fehlerhaften Textauszug.

3.	Die Korrektur oder Erklärung, warum es ein Fehler ist.

Du bist ein sehr genauer Reviewer für medizinische Fachtexte (insbesondere Onkopedia / DGHO-Leitlinien). Bitte arbeite SCHRITT FÜR SCHRITT und EXTREM sorgfältig. AUFGABE Ich gebe dir: 1. Die URL einer Onkopedia-Leitlinie, 2. den (ggf. kopierten) Text dieser Leitlinie, 3. eine eigene Liste von vermuteten Fehlern (sprachlich + inhaltlich). Deine Aufgaben: A) Prüfe zuerst, ob der von mir eingefügte Text zur angegebenen URL passt (z.B. keine größeren Copy-Paste-Lücken, keine gravierenden Abweichungen). Wenn es relevante Unterschiede gibt, schreibe das kurz dazu. B) Prüfe dann die gesamte Leitlinie so gründlich wie möglich: - a) auf Rechtschreib-, Grammatik-, Syntax- und klar erkennbare Formatierungsfehler, - b) auf inhaltliche/fachliche Fehler, Unklarheiten oder Widersprüche (Zahlen, Prozente, Studienergebnisse, Scores, Stadien, Protokoll-Namen, Akronyme, Klassifikationen, Altersangaben, etc.). C) Prüfe meine vorgeschlagenen Fehler mit: - „Ja, stimmt“ / „Teilweise richtig“ / „Eher nicht korrekt“ (mit kurzer Begründung), - und ergänze alle Fehler, die ich übersehen habe. D) Verwende – wo sinnvoll – die Originalpublikationen / offiziellen Quellen (z.B. ECHELON-2, PINK/PINK-E, WHO-HAEM5, ICC, EBMT-Empfehlungen) zur Überprüfung von Zahlen, Definitionen und Protokollnamen. WICHTIG - Arbeite auf Deutsch. - Stelle KEINE Rückfragen, sondern triff bei Unklarheiten eine bestmögliche, begründete Einschätzung. - Wenn Text und Online-Version widersprüchlich sind, orientiere dich primär an der Online-Version, aber benenne den Widerspruch. - Wenn du dir bei einem inhaltlichen Punkt nicht sicher bist, schreibe das ausdrücklich dazu („mit Unsicherheit“, „Datenlage unklar“). - Unterscheide immer klar zwischen: • sicher falschen Angaben, • fraglich/unplausibel, • nur stilistisch/terminologisch unsauber. AUSGABEFORMAT 1. KURZE EINLEITUNG (max. 10 Zeilen) - 1–2 Sätze zur Leitlinie (Thema, Stand), - Ob dein Abgleich zwischen URL und eingefügtem Text Auffälligkeiten zeigt. 2. BEWERTUNG MEINER EIGENEN FEHLERLISTE - Gehe Punkt für Punkt meine vorgeschlagenen Fehler durch. - Für jeden Punkt: - Kurze Überschrift: „Mein Punkt X: …“ - Deine Bewertung: „Ja, stimmt“, „teilweise richtig“ oder „eher nicht korrekt“. - Falls nötig: 2–4 Sätze Erklärung, inklusive ggf. Hinweis auf Studien/Leitlinien. 3. ZWEI FINALE GESAMTLISTEN 3.1 Liste 1 – Rechtschreib-/Grammatik-/Syntax-/Formatierungsfehler - Nur klare, objektive Fehler (keine reinen Geschmacksfragen). - Jeder Eintrag mit folgendem Schema: • Abschnitt/Ort (so genau wie möglich, z.B. „Kapitel 5.3.2 Rezidivtherapie, 1. Absatz“) • Originalformulierung (genauer Ausschnitt) • Problem (z.B. Tippfehler, falscher Kasus, zerstörter Satz, falsches Akronym, Klammerfehler) • Konkreter Korrekturvorschlag - Ähnliche Fehler (z.B. immer gleiche falsche Schreibweise eines Akronyms) bitte gruppiert nennen („kommt mehrfach vor, z.B. in…“). 3.2 Liste 2 – Inhaltliche/fachliche Fehler, Widersprüche oder kritische Ungenauigkeiten - Fokus auf: • Falsche Zahlen und Prozente (z.B. Studienarme, CR-Raten, Medianalter), • Verwechslung oder falsche Definition von Scores (IPI, PIT, PINK/PINK-E, etc.), • Falsche Akronyme (z.B. ANKL vs. ANKTL, DDGP vs. DDPG), • Falsche oder missverständliche Aussagen zu Leitlinienempfehlungen, Stadien, Entitäten, Klassifikationen, • Inkonsistenzen innerhalb der Leitlinie (z.B. widersprüchliche Therapiestrategien in zwei Kapiteln). - Für jeden Eintrag: • Abschnitt/Ort, • Originalaussage (kurz), • Warum fachlich problematisch (inkl. kurzer Verweis auf Leitlinie/Studie/Klassifikation), • Konkreter Änderungsvorschlag (idealerweise als alternative Formulierung). 4. OPTIONALER ABSCHLUSS (max. 5–6 Zeilen) - Sehr knappe Zusammenfassung, was die aus deiner Sicht „kritischsten“ 3–5 Punkte sind, die in einer Revision der Leitlinie unbedingt zuerst geändert werden sollten.

Hier sind die Daten:

- URL: …
- Vollständiger (oder relevanter) Text der Leitlinie: …
- Fehlerliste: …

# 5. Prompt for error detection in Onkopedia guidelines (English)

Role:

You are a meticulous medical editor and oncologist specializing in evidence-based guidelines (e.g., DGHO/Onkopedia). Your task is to thoroughly proofread and review the content of a guideline text.

Context:

I will give you the URL and the complete text of a new Onkopedia guideline in a moment. The text often comes from a CMS export or copy-paste process and therefore contains specific formatting errors and inconsistencies in content. Important: The document may have a date in the future (e.g., “as of October 2025”). Please do not consider future dates or references to conferences in 2025 as errors, but accept them as the current status of the document.

Task:

Please analyze the text step by step and create two separate lists of errors. Be extremely accurate and meticulous.

List 1: Spelling, grammar, formatting, and expression

Pay attention to:

• Typos: (e.g., “Lymhome” instead of “Lymphoma,” missing letters).

• Formatting errors: (e.g., missing spaces in ICD codes such as “ICD-10C15,” footnote numbers stuck in the body text, copy-paste remnants such as “LINK” in the text).

• Grammar: (agreement errors, case errors, incorrect references).

• Sentence structure: (fragmented sentences, syntax destroyed by edits).

• Expression: (incorrect translations from English, e.g., “irrigation” instead of ‘hydration’/“watering,” inappropriate terms).

List 2: Content errors, inconsistencies, and logic

Pay attention to:

• Contradictions: (Is there a difference between the body text and the table? Do the treatment algorithm and the text contradict each other?).

• Acronyms: (Are abbreviations used incorrectly, e.g., “DDPG” instead of “DDGP”?).

• Medical plausibility: (Are dosages roughly correct? Are mutation names correct? Are percentages mathematically logical?).

• Referencing: (Are studies cited incorrectly or figures transferred incorrectly?).

• Outdated information: (Are drugs that have long been standard referred to as “new,” or are important warnings missing?).

Output format:

Please list the errors in a clear list. For each error, state:

1. The location (chapter/section).

2. The incorrect text excerpt.

3. The correction or explanation of why it is an error.

You are a very accurate reviewer of medical texts (especially Onkopedia / DGHO guidelines). Please work STEP BY STEP and EXTREMELY carefully. TASK I will give you: 1. The URL of an Onkopedia guideline, 2. the text of this guideline (copied if necessary), 3. my own list of suspected errors (linguistic + content-related). Your tasks: A) First, check whether the text I have inserted matches the specified URL (e.g., no major copy-paste gaps, no serious deviations). If there are any relevant differences, please note this briefly. B) Then check the entire guideline as thoroughly as possible: - a) for spelling, grammar, syntax, and clearly recognizable formatting errors, - b) for content/technical errors, ambiguities, or contradictions (numbers, percentages, study results, scores, stages, protocol names, acronyms, classifications, age information, etc.). C) Check my suggested errors with: - “Yes, correct” / “Partially correct” / “Probably incorrect” (with a brief explanation), - and add any errors that I have overlooked. D) Where appropriate, use the original publications / official sources (e.g., ECHELON-2, PINK/PINK-E, WHO-HAEM5, ICC, EBMT recommendations) to verify figures, definitions, and protocol names. IMPORTANT - Work in German. - Do NOT ask questions, but make the best possible, reasoned assessment in case of uncertainty. - If the text and online version contradict each other, refer primarily to the online version, but point out the contradiction. - If you are unsure about a point of content, explicitly state this (“with uncertainty,” “data unclear”). - Always clearly distinguish between: • definitely incorrect information, • questionable/implausible, • only stylistically/terminologically inaccurate. OUTPUT FORMAT 1. SHORT INTRODUCTION (max. 10 lines) - 1–2 sentences about the guideline (topic, status), - Whether your comparison between the URL and the inserted text reveals any anomalies. 2. EVALUATION OF MY OWN LIST OF ERRORS - Go through my suggested errors point by point.

- For each point: - Short heading: “My point X: ...” - Your evaluation: “Yes, correct,” “partially correct,” or “not quite correct.” - If necessary: 2–4 sentences of explanation, including references to studies/guidelines if applicable. 3. TWO FINAL OVERALL LISTS 3.1 List 1 – Spelling/grammar/syntax/formatting errors - Only clear, objective errors (no matters of taste). - Each entry with the following structure: • Section/location (as precise as possible, e.g., “Chapter 5.3.2 Recurrence therapy, 1st paragraph”) • Original wording (exact excerpt) • Problem (e.g., typo, incorrect case, broken sentence, incorrect acronym, bracket error) • Specific correction suggestion - Please group similar errors (e.g., consistently incorrect spelling of an acronym) (“occurs multiple times, e.g., in...”). 3.2 List 2 – Content/technical errors, contradictions, or critical inaccuracies – Focus on: • Incorrect figures and percentages (e.g., study arms, CR rates, median age), • Confusion or incorrect definition of scores (IPI, PIT, PINK/PINK-E, etc.), • Incorrect acronyms (e.g., ANKL vs. ANKTL, DDGP vs. DDPG), • Incorrect or misleading statements regarding guideline recommendations, stages, entities, classifications, • Inconsistencies within the guideline (e.g., contradictory treatment strategies in two chapters). - For each entry: • Section/location, • Original statement (brief), • Why it is technically problematic (including a brief reference to the guideline/study/classification), • Specific proposed change (ideally as an alternative wording). 4. OPTIONAL CONCLUSION (max. 5–6 lines) - Very brief summary of what you consider to be the 3–5 most critical points that should be changed first in a revision of the guideline.

Here is the data:

- URL: …

- Complete (or relevant) text of the guideline: …

- List of errors: …

# 6. GPT o4-mini-high output (German)

### Sektion: Neue Klassifikation (WHO 5. Auflage und ICC 2022)

**Kernaussage:** Die aktualisierten hämatopathologischen Klassifikationen (WHO 5. Auflage 2022 und International Consensus Classification 2022) brachten bedeutsame Änderungen für PTCL: Nodal auftretende T-Follikelhelfer-Zell-Lymphome (TFH-Lymphome) werden nun als gemeinsame Kategorie geführt, welche Angioimmunoblastisches T-Zell-Lymphom (AITL), follikuläres T-Zell-Lymphom und andere TFH-T-Zell-Lymphome umfasst[[1]](https://pmc.ncbi.nlm.nih.gov/articles/PMC10845937/#:~:text=PTCL,lymphomas%20has%20been%20introduced%2C%20showing)[[2]](https://pmc.ncbi.nlm.nih.gov/articles/PMC10845937/#:~:text=cell%20lymphoma). Zudem wird das primär nodale, EBV-assoziierte T-/NK-Zell-Lymphom erstmals als eigene Entität anerkannt (in WHO-5 als „EBV+ nodales T/NK-Zell-Lymphom“) und nicht mehr bloß als Variante von PTCL-NOS[[2]](https://pmc.ncbi.nlm.nih.gov/articles/PMC10845937/#:~:text=cell%20lymphoma). Insgesamt bleiben die Unterschiede zwischen WHO und ICC terminologisch, nicht inhaltlich – beide reflektieren den gewachsenen molekularen Erkenntnisstand und präzisieren die Abgrenzung der Subtypen.

**Studiendetails/Quelle:**
 *Studie/Publikation:* *Überblick WHO-HAEM5 & ICC (Alaggio* et al*.,* Leukemia *2022; Amador & Chan,* Cancer Biol Med *2024)*[[1]](https://pmc.ncbi.nlm.nih.gov/articles/PMC10845937/#:~:text=PTCL,lymphomas%20has%20been%20introduced%2C%20showing)[[2]](https://pmc.ncbi.nlm.nih.gov/articles/PMC10845937/#:~:text=cell%20lymphoma)
 **Kernthema:** Neue Taxonomie der nodalen PTCL-Subtypen (TFH-Kategorie, eigene Entität für EBV+ PTCL, Beibehaltung PTCL-NOS mit Ausschlusskriterien)
 *Relevanz:** Aktualisierung der diagnostischen Definitionen für PTCL-Subtypen in Klassifikationen der hämatologischen Neoplasien

**Evidenzlevel/Empfehlungsgrad (geschätzt):** Evidenzlevel IV (Expertenkonsens in WHO/ICC); Empfehlungsgrad A (starke Empfehlung zur Anpassung der Nomenklatur in Leitlinien, da internationale Konsensklassifikation).

**Implikation für die Onkopedia-Leitlinie:** Die Leitlinie sollte die geänderte Nomenklatur übernehmen und beschreiben. Insbesondere sind TFH-Zell-Lymphome als eigene Gruppe auszuweisen (anstelle der früheren Sammelkategorie AITL ± Varianten)[[1]](https://pmc.ncbi.nlm.nih.gov/articles/PMC10845937/#:~:text=PTCL,lymphomas%20has%20been%20introduced%2C%20showing), und das „EBV-positive nodale T/NK-Zell-Lymphom“ ist als eigenständige Entität aufzunehmen[[2]](https://pmc.ncbi.nlm.nih.gov/articles/PMC10845937/#:~:text=cell%20lymphoma). Die diagnostischen Kriterien für PTCL-NOS sind entsprechend enger zu fassen (Ausschluss von Fällen mit TFH-Phänotyp). Eine vereinheitlichte Terminologie verbessert Kommunikation, Diagnostik und ermöglicht präzisere Therapieempfehlungen.

### Sektion: Molekulare Marker und Risikostratifizierung bei PTCL

**Kernaussage:** Neuere molekulare Analysen haben prognostisch wichtige Subgruppen von PTCL bestätigt, die diagnostisch berücksichtigt werden sollten. Bei PTCL-NOS lassen Genexpressionsprofile zwei Hauptgruppen erkennen: eine GATA3-dominierte Signatur vs. eine TBX21-dominierte (T-bet) Signatur[[3]](https://pmc.ncbi.nlm.nih.gov/articles/PMC10845937/#:~:text=Gene%20expression%20profiling%20has%20highlighted,Moreover%2C%20a%20digital%20gene)[[4]](https://pmc.ncbi.nlm.nih.gov/articles/PMC10845937/#:~:text=PTCL). Die *GATA3*-Subgruppe zeigt einen deutlich ungünstigeren Verlauf (vermehrte genetische Aberrationen, deletierte Tumorsuppressorgene) und schlechteres Überleben als die *TBX21*-Subgruppe[[1]](https://pmc.ncbi.nlm.nih.gov/articles/PMC10845937/#:~:text=PTCL,lymphomas%20has%20been%20introduced%2C%20showing)[[5]](https://pmc.ncbi.nlm.nih.gov/articles/PMC10845937/#:~:text=). Ebenso haben zytogenetische Marker bei ALK-negativem ALCL hohen Stellenwert: Ein *DUSP22*-Rearrangement markiert eine prognostisch günstigere Variante mit ~90% 5-Jahres-Überleben – ähnlich dem ALK-positiven ALCL – während ein *TP63*-Rearrangement mit einer äußerst schlechten Prognose einhergeht (ca. 17% 5-Jahres-Überleben)[[6]](https://newsnetwork.mayoclinic.org/discussion/mayo-clinic-recommends-new-routine-testing-for-some-non-hodgkin-lymphomas/#:~:text=Patients%20whose%20lymphomas%20had%20TP63,with%20an%20intermediate%20survival%20rate)[[7]](https://newsnetwork.mayoclinic.org/discussion/mayo-clinic-recommends-new-routine-testing-for-some-non-hodgkin-lymphomas/#:~:text=TP63,a%20Damon%20Runyon%20Clinical%20Investigator). Diese Biomarker sind durch FISH bzw. Immunhistochemie detektierbar und erlauben eine feinere Risikostratifizierung der Patienten.

**Studiendetails/Quelle:**
 *Studien:* *(a) Gene-Expression-Studien zu PTCL-NOS (Iqbal* et al*., 2014; Amador* et al*., 2024)*[[3]](https://pmc.ncbi.nlm.nih.gov/articles/PMC10845937/#:~:text=Gene%20expression%20profiling%20has%20highlighted,Moreover%2C%20a%20digital%20gene)[[4]](https://pmc.ncbi.nlm.nih.gov/articles/PMC10845937/#:~:text=PTCL)*. (b) Multizentrische FISH-Analyse ALK– ALCL (Feldman* et al*.,* Blood *2014)*[[6]](https://newsnetwork.mayoclinic.org/discussion/mayo-clinic-recommends-new-routine-testing-for-some-non-hodgkin-lymphomas/#:~:text=Patients%20whose%20lymphomas%20had%20TP63,with%20an%20intermediate%20survival%20rate)[[7]](https://newsnetwork.mayoclinic.org/discussion/mayo-clinic-recommends-new-routine-testing-for-some-non-hodgkin-lymphomas/#:~:text=TP63,a%20Damon%20Runyon%20Clinical%20Investigator)
 **Patientenpopulation:** PTCL-NOS-Kohorten mit genomischen Profilen; ALK-negative ALCL-Fälle aus internationalen Registern
 *Ergebnisse:* *Identifikation distinkter molekularer Subentitäten: PTCL-GATA3 vs. PTCL-TBX21 mit unterschiedlicher Genetik und Prognose*[[1]](https://pmc.ncbi.nlm.nih.gov/articles/PMC10845937/#:~:text=PTCL,lymphomas%20has%20been%20introduced%2C%20showing)[[5]](https://pmc.ncbi.nlm.nih.gov/articles/PMC10845937/#:~:text=)*; bei ALK– ALCL stark divergierende Überlebensraten abhängig von* DUSP22*- bzw.* TP63*-Status*[[6]](https://newsnetwork.mayoclinic.org/discussion/mayo-clinic-recommends-new-routine-testing-for-some-non-hodgkin-lymphomas/#:~:text=Patients%20whose%20lymphomas%20had%20TP63,with%20an%20intermediate%20survival%20rate)
 **Endpunkte:** Korrelation molekularer Marker mit Gesamtüberleben/PFS; Etablierung prognostischer Klassen innerhalb morphologischer Subtypen

**Evidenzlevel/Empfehlungsgrad (geschätzt):** Evidenzlevel IIb (retrospektive multizentrische Kohortenanalysen); Empfehlungsgrad B (Empfehlung zur Durchführung dieser Biomarker-Testungen, da klinisch relevant für Prognose, aber keine prospektiven Therapiestudien hierzu).

**Implikation für die Onkopedia-Leitlinie:** Es sollte empfohlen werden, bei PTCL-NOS eine weitere immunhistochemische Charakterisierung (GATA3 vs. T-bet Expression) durchzuführen, da dies auf das Risikoprofil hinweist[[3]](https://pmc.ncbi.nlm.nih.gov/articles/PMC10845937/#:~:text=Gene%20expression%20profiling%20has%20highlighted,Moreover%2C%20a%20digital%20gene)[[5]](https://pmc.ncbi.nlm.nih.gov/articles/PMC10845937/#:~:text=). Für ALK-negatives ALCL wird eine routinemäßige FISH-Testung auf *DUSP22*- und *TP63*-Rearrangements angeraten[[6]](https://newsnetwork.mayoclinic.org/discussion/mayo-clinic-recommends-new-routine-testing-for-some-non-hodgkin-lymphomas/#:~:text=Patients%20whose%20lymphomas%20had%20TP63,with%20an%20intermediate%20survival%20rate). Patienten mit *DUSP22*-rearrangiertem ALCL könnten weniger aggressive Therapiestrategien benötigen, wohingegen *TP63*-rearrangierte Fälle für experimentelle Ansätze oder frühzeitige Transplantation in Betracht gezogen werden sollten. Die Leitlinie sollte diese Marker als festen Bestandteil der Diagnostik etablieren, um eine risikoadaptierte Therapieplanung zu ermöglichen.

### Sektion: CD30-Expressionsbestimmung und Schwellenwert für Brentuximab

**Kernaussage:** Die CD30-Expression bleibt ein zentraler prädiktiver Marker für den Einsatz von Brentuximab Vedotin (BV). Standardmäßig erfolgt der Nachweis mittels Immunhistochemie; in Studien wurde ein Schwellenwert von ≥10% CD30-positiven Tumorzellen verwendet, um „CD30-positive“ PTCL zu definieren[[8]](https://pmc.ncbi.nlm.nih.gov/articles/PMC9447792/#:~:text=brentuximab%20vedotin%2C%20an%20antibody,Brentuximab%20vedotin%20was%20approved%20in). Nach aktueller Evidenz ist dieser Cut-off pragmatisch, jedoch nicht absolut: In ECHELON-2 waren wenige Patienten mit <10% CD30 positiv eingeschlossen, und explorative Analysen deuten an, dass auch Fälle mit niedriger CD30-Expression von BV-CHP profitieren könnten[[9]](https://www.sciencedirect.com/science/article/pii/S0006497120731137#:~:text=ScienceDirect%20www,CD30%2B%20after). Eine prospektive Phase-III-Studie (NCT04569032) rekrutiert derzeit Patienten mit <10% CD30-Expression, um den Nutzen von BV+CHP in diesem Kollektiv zu prüfen. Methodisch gab es keine grundlegende Änderung – die Reproduzierbarkeit der IHC-Bestimmung hat sich verbessert, jedoch sollte die Interpretation stets in erfahrenen hämatopathologischen Zentren erfolgen.

**Studiendetails/Quelle:**
 *Studie:* *ECHELON-2 (Einschlusskriterium CD30 ≥10%; Post-hoc Analyse geringer Expresser)*[[8]](https://pmc.ncbi.nlm.nih.gov/articles/PMC9447792/#:~:text=brentuximab%20vedotin%2C%20an%20antibody,Brentuximab%20vedotin%20was%20approved%20in)[[9]](https://www.sciencedirect.com/science/article/pii/S0006497120731137#:~:text=ScienceDirect%20www,CD30%2B%20after)
 **Publikation:** Horwitz *et al*., *Ann Oncol* 2022 (5-Jahres-Update ECHELON-2)
 *Patientenpopulation:* *Neu diagnostizierte PTCL, die nicht ALK+ ALCL waren, mit mindestens 10% CD30-Expression; wenige Fälle mit 1–<10% CD30 wurden retrospektiv analysiert*
 **Endpunkte:** PFS/OS-Vergleich A+CHP vs. CHOP in Abhängigkeit von CD30-Level; Definition der Ansprechrate in niedrig CD30-exprimierenden Tumoren

**Evidenzlevel/Empfehlungsgrad (geschätzt):** Evidenzlevel III (post-hoc Subgruppenanalyse bzw. Expertenkonsens zum Cut-off); Empfehlungsgrad B (moderate Empfehlung, den 10%-Cut-off als Richtschnur zu verwenden, jedoch im Einzelfall klinisch zu interpretieren).

**Implikation für die Onkopedia-Leitlinie:** Die Leitlinie sollte klarstellen, dass BV+CHP für PTCL mit ≥10% CD30-Expression empfohlen wird (entsprechend Studiendesign)[[8]](https://pmc.ncbi.nlm.nih.gov/articles/PMC9447792/#:~:text=brentuximab%20vedotin%2C%20an%20antibody,Brentuximab%20vedotin%20was%20approved%20in). Gleichzeitig kann erwähnt werden, dass bei niedrigpositiven Fällen (<10%) eine indivduelle Therapieentscheidung getroffen werden muss – etwa im Rahmen klinischer Studien oder nach interdisziplinärer Bewertung – da hier ein potenzieller Benefit nicht ausgeschlossen ist[[9]](https://www.sciencedirect.com/science/article/pii/S0006497120731137#:~:text=ScienceDirect%20www,CD30%2B%20after). Der dokumentierte CD30-Status sollte in jedem Pathologiebericht enthalten sein; Änderungen des IHC-Protokolls oder neue Technologien (z.B. quantitative Bildanalyse) sind weiter zu beobachten, um ggf. künftig präzisere Cut-offs definieren zu können.

### Sektion: Erstlinientherapie CD30-positiver PTCL (insb. ALCL) – ECHELON-2

**Kernaussage:** Die ECHELON-2-Studie und ihre Langzeit-Auswertung etablieren **Brentuximab Vedotin + CHP (A+CHP)** als neuen Therapiestandard für CD30-exprimierende PTCL, insbesondere systemische ALCL. Im 5-Jahres-Follow-up zeigte A+CHP gegenüber CHOP ein signifikant verlängertes progressionsfreies Überleben (5-Jahres-PFS 51,4% vs. 43,0%, HR=0,70) und einen anhaltenden Vorteil im Gesamtüberleben (5-Jahres-OS 70,1% vs. 61,0%, HR=0,72)[[10]](https://pmc.ncbi.nlm.nih.gov/articles/PMC9447792/#:~:text=A%20total%20of%20452%20patients,Among%20patients%20who)[[11]](https://pmc.ncbi.nlm.nih.gov/articles/PMC9447792/#:~:text=0.53%E2%80%930.91%29%2C%20and%205,subsequent%20brentuximab%20vedotin%20after%20CHOP). Dieser Überlebensgewinn war konsistent über wesentliche Subgruppen – einschließlich sowohl ALK+ als auch ALK- ALCL – hinweg zu beobachten. Neben höherer Wirksamkeit zeigte sich A+CHP verträglich: Die periphere Neuropathie war beherrschbar und bildete sich bei der Mehrheit der Patienten zurück[[12]](https://pmc.ncbi.nlm.nih.gov/articles/PMC9447792/#:~:text=75.9,subsequent%20brentuximab%20vedotin%20after%20CHOP). Damit untermauern die Langzeitdaten, dass A+CHP der konventionellen CHOP-Chemotherapie klar überlegen ist und für CD30-positive PTCL in der Erstlinie bevorzugt werden sollte.

**Studiendetails/Quelle:**
 *Studie:* *ECHELON-2 – randomisierte Phase III (A+CHP vs. CHOP)*
 **Publikation:** Horwitz S *et al*., *Annals of Oncology*, 2022 (5-Year Analysis)[[10]](https://pmc.ncbi.nlm.nih.gov/articles/PMC9447792/#:~:text=A%20total%20of%20452%20patients,Among%20patients%20who)[[11]](https://pmc.ncbi.nlm.nih.gov/articles/PMC9447792/#:~:text=0.53%E2%80%930.91%29%2C%20and%205,subsequent%20brentuximab%20vedotin%20after%20CHOP)
 *Patientenpopulation:* *n=452 Patienten mit neu diagnostiziertem CD30-positivem PTCL (inkl. ~70% sALCL [ALK+ und ALK-] sowie andere PTCL mit CD30≥10%)*
 **Endpunkte:** Primärer Endpunkt: PFS (unabhängiges Review); wichtiger sekundärer Endpunkt: OS. Weitere Endpunkte: ORR, CR-Rate, Sicherheit (v.a. Neuropathie)

**Evidenzlevel/Empfehlungsgrad (geschätzt):** Evidenzlevel Ib (randomisierte kontrollierte Phase-III-Studie); Empfehlungsgrad A (starke Empfehlung für A+CHP als Erstlinientherapie bei CD30+ PTCL aufgrund signifikant besserer klinischer Outcomes)

**Implikation für die Onkopedia-Leitlinie:** Die Leitlinie sollte **Brentuximab Vedotin + CHP** explizit als bevorzugte Erstlinientherapie für alle CD30-positiven PTCL nennen. Insbesondere für systemische ALCL (ALK+ *und* ALK-) ersetzt BV-CHP den bisherigen CHOP-Standard[[11]](https://pmc.ncbi.nlm.nih.gov/articles/PMC9447792/#:~:text=0.53%E2%80%930.91%29%2C%20and%205,subsequent%20brentuximab%20vedotin%20after%20CHOP). Aufgrund der vorliegenden OS-Vorteile[[10]](https://pmc.ncbi.nlm.nih.gov/articles/PMC9447792/#:~:text=A%20total%20of%20452%20patients,Among%20patients%20who) gilt dies unabhängig vom Alter (sofern BV vertragen wird) und sollte – falls noch nicht erfolgt – Eingang in die Zulassungsempfehlungen finden. Onkopedia sollte zudem darauf hinweisen, dass BV-CHP inzwischen in Europa und den USA zugelassen ist für unbehandelte CD30-positive PTCL[[13]](https://pmc.ncbi.nlm.nih.gov/articles/PMC9447792/#:~:text=69.1,expressing%20PTCL%20and). Insgesamt wird durch diese Daten die Bedeutung einer CD30-Testung bei Diagnosestellung unterstrichen, um geeignete Patienten für BV-CHP zu identifizieren.

### Sektion: Erstlinientherapie CD30-negative PTCL – CHOP-Alternativen und neue Kombinationen

**Kernaussage:** Für CD30-niedrig/negative PTCL (wie PTCL-NOS, AITL) bleibt CHOP/CHOEP mangels überzeugender Verbesserungen weiterhin der Hauptpfeiler der Erstlinienbehandlung. Eine große randomisierte Studie (Ro-CHOP, LYSA) zeigte, dass die Zugabe des HDAC-Inhibitors **Romidepsin zu CHOP** keinen Nutzen bringt: PFS, Ansprechrate und OS unterschieden sich nicht signifikant von CHOP allein, hingegen traten mehr Toxizitäten (hämatologisch und infektionsbedingt) im Ro-CHOP-Arm auf[[14]](https://pubmed.ncbi.nlm.nih.gov/34843406/#:~:text=LYSA%29%20pubmed,emergent)[[15]](https://ascopubs.org/doi/10.1200/JCO.23.02658#:~:text=Publications%20ascopubs,of%20romidepsin%20in%20PTCL%2C). Diese negativen Ergebnisse führten sogar zur freiwilligen Indikationsrücknahme von Romidepsin in der EU für PTCL[[15]](https://ascopubs.org/doi/10.1200/JCO.23.02658#:~:text=Publications%20ascopubs,of%20romidepsin%20in%20PTCL%2C). Auf der anderen Seite gibt es vielversprechende Signale von neuen Kombinationen, speziell im biologisch definierten Subtyp AITL/TFH-PTCL: Die **Kombination von oralem Azacitidin (CC-486) mit CHOP** erreichte in einer Phase-II-Studie eine hohe Komplettremissionsrate von 75% (88% bei TFH-Subtypen) und 2-Jahres-PFS-Raten ~65-69%[[16]](https://pubmed.ncbi.nlm.nih.gov/36796016/#:~:text=toxicities%20were%20mostly%20neutropenia%20%2871,486)[[17]](https://pubmed.ncbi.nlm.nih.gov/36796016/#:~:text=symptoms%20%285,of%20the%20tumor%20microenvironment%20by) – deutlich höher als historisch unter CHOP erwartet. Diese HMA-CHOP-Kombination wurde gut vertragen (keine übermäßige zusätzliche Toxizität außer erhöhten, aber handhabbaren Zytopenien)[[18]](https://pubmed.ncbi.nlm.nih.gov/36796016/#:~:text=oral%20azacitidine%20%28CC,for%20PTCL). Insgesamt haben sich also **keine neuen Phase-III-Standards** durchgesetzt, aber selektive Add-on-Strategien (Epigenetik, Antikörper) in Phase-II zeigen Potential, welches nun in laufenden Studien (z.B. randomisierte Studie Alliance A051902 für CHOP+Azacitidin) validiert wird[[19]](https://pubmed.ncbi.nlm.nih.gov/36796016/#:~:text=upregulation%20of%20genes%20related%20to,gov%20as).

**Studiendetails/Quelle:**
 *Studie 1:* *Ro-CHOP Phase III (Romidepsin + CHOP* vs. *CHOP) –* *Ergebnis:* *Kein PFS/OS-Vorteil durch Romidepsin, höhere Toxizität*[[14]](https://pubmed.ncbi.nlm.nih.gov/34843406/#:~:text=LYSA%29%20pubmed,emergent)[[15]](https://ascopubs.org/doi/10.1200/JCO.23.02658#:~:text=Publications%20ascopubs,of%20romidepsin%20in%20PTCL%2C) *(Pressemitteilung & JCO 2022 Abstract)*
 **Studie 2:** Oral Azacitidin + CHOP, Phase II (Ruan J *et al*., *Blood* 2023) – **Ergebnis:** CR-Rate 75% (bei TFH 88%), 2-Jahres-OS ~68%[[16]](https://pubmed.ncbi.nlm.nih.gov/36796016/#:~:text=toxicities%20were%20mostly%20neutropenia%20%2871,486)[[17]](https://pubmed.ncbi.nlm.nih.gov/36796016/#:~:text=symptoms%20%285,of%20the%20tumor%20microenvironment%20by). * **Patientenpopulation:** Studie 1: n~400, gemischte PTCL (ohne ALK+ ALCL), medianes Alter ~60; Studie 2: n=20, vorwiegend AITL/TFH-PTCL, median ~63 Jahre
 *Endpunkte:** Studie 1: Primär PFS, sekundär ORR/OS (kein Benefit durch Zusatz); Studie 2: Primär CR-Rate nach Induktion, Sekundär 2-J-PFS/OS (hohe Remissionsraten beobachtet)

**Evidenzlevel/Empfehlungsgrad (geschätzt):** Evidenzlevel Ib (Studie 1, neg. RCT) und IIa (Studie 2, prospektive Phase II); Empfehlungsgrad A für *Nicht*-Einsatz von Romidepsin zusätzlich zu CHOP (klarer Negativbefund)[[14]](https://pubmed.ncbi.nlm.nih.gov/34843406/#:~:text=LYSA%29%20pubmed,emergent), Empfehlungsgrad C für Azacitidin+CHOP (vielversprechend, aber noch experimentell – nur im Studienkontext zu empfehlen).

**Implikation für die Onkopedia-Leitlinie:** Onkopedia sollte betonen, dass **CHO(E)P nach wie vor Standard** bei CD30-negativen PTCL ist, da neue Kombinationen bisher keinen gesicherten Mehrwert in Phase III gezeigt haben. Insbesondere ist **vom routinemäßigen Zusatz von Romidepsin zu CHOP abzuraten** (kein Benefit, erhöhte Toxizität)[[14]](https://pubmed.ncbi.nlm.nih.gov/34843406/#:~:text=LYSA%29%20pubmed,emergent). Gleichzeitig kann die Leitlinie auf innovative Therapiekonzepte hinweisen: Für TFH-Subtypen wie AITL könnte die Ergänzung von **Hypomethylanz** (Azacitidin) eine zukünftige Option darstellen – allerdings vorerst nur im Rahmen von Studien oder individuellen Heilversuchen[[16]](https://pubmed.ncbi.nlm.nih.gov/36796016/#:~:text=toxicities%20were%20mostly%20neutropenia%20%2871,486). Insgesamt sollte der Tenor sein, dass außerhalb klinischer Studien **kein neues Chemotherapie-basierte Regime CHOP eindeutig überlegen** war. Daher bleibt CHO(E)P ± konsolidierende Auto-SZT der empfohlene Weg, und Patienten mit CD30-negativen PTCL sollten nach Möglichkeit in Studienprotokolle eingeschlossen werden, die neue Add-ons evaluieren.

### Sektion: Konsolidierung in erster Remission – autologe vs. allogene Transplantation

**Kernaussage:** Die optimale Rolle einer Stammzelltransplantation (SZT) als Konsolidierung nach Erstlinientherapie wurde in den letzten Jahren klarer umrissen, bleibt aber subtypabhängig. Eine große multinationale retrospektive Studie (GELTAMO/FIL 2022) deutet an, dass **autologe SZT in erster kompletter Remission (CR1)** das Fortschreiten der Krankheit verzögern kann: Die 5-Jahres-PFS betrug 63% mit upfront Auto-SZT vs. 48% ohne Transplantation (p=0.042)[[20]](https://haematologica.org/article/view/haematol.2021.279426#:~:text=decided%20against%20it,These%20results%20should). In einer multivariaten Analyse war die upfront Auto-SZT mit einem signifikant reduzierten Risiko für Progression oder Tod assoziiert (HR ~0,57)[[21]](https://haematologica.org/article/view/haematol.2021.279426#:~:text=transplanted%20group%3A%2063,These%20results%20should). Ein OS-Vorteil zeigte sich besonders bei fortgeschrittener Erkrankung (70% vs. 50% 5-Jahres-OS in Stadium III/IV)[[20]](https://haematologica.org/article/view/haematol.2021.279426#:~:text=decided%20against%20it,These%20results%20should). Diese Ergebnisse stützen die gängige Praxis, bei fitten Patienten (insb. PTCL-NOS, AITL, ALK- ALCL) nach Induktion in CR eine Auto-SZT durchzuführen – auch wenn ein Beweis durch RCT weiterhin fehlt. **Allogene SZT** wird in der Firstline-Konsolidierung eher zurückhaltend eingesetzt und nur in speziellen Hochrisiko-Situationen diskutiert (z.B. chemorefraktäre Erkrankung, seltene sehr aggressive Entitäten wie HSTL). Neuere Registerdaten legen nahe, dass die allo-SZT im Rezidivfall eine 3-Jahres-Überlebensrate von ~30–40% erreichen kann, was bei geeigneten Patienten einen kurativen Ansatz darstellt[[22]](https://www.frontiersin.org/journals/oncology/articles/10.3389/fonc.2022.1039888/full#:~:text=,cell%20lymphoma). Insgesamt verdichten sich die Hinweise, dass **ein individueller, risikoadaptierter Ansatz** sinnvoll ist: Jüngere Hochrisiko-Patienten profitieren von Transplantationskonsolidation, während bei niedrigem Risiko (z.B. ALK+ ALCL in kompletter Remission) eine Auto-SZT möglicherweise nicht nötig ist.

**Studiendetails/Quelle:**
 *Studie:* *Retrospektive multizentrische Studie zur Firstline-ASCT (GELTAMO/FIL, 2001–2011 Kohorte)*[[20]](https://haematologica.org/article/view/haematol.2021.279426#:~:text=decided%20against%20it,These%20results%20should)
 **Publikation:** Corradini P *et al*., *Haematologica* 2022 (Analyse von 174 PTCL-Patienten in CR1 mit vs. ohne Auto-SZT)[[20]](https://haematologica.org/article/view/haematol.2021.279426#:~:text=decided%20against%20it,These%20results%20should)
 *Patientenpopulation:* *PTCL (versch. Subtypen,* ohne *ALK+ ALCL) in CR1 nach Induktion; 103 mit geplanter Auto-SZT, 71 ohne (ärztliche Entscheidung)*
 **Endpunkte:** 5-Jahres-PFS mit vs. ohne Auto-SZT (63% vs. 48%[[20]](https://haematologica.org/article/view/haematol.2021.279426#:~:text=decided%20against%20it,These%20results%20should)); 5-Jahres-OS in Subgruppen (Benefit insb. bei fortgeschrittenem Stadium[[20]](https://haematologica.org/article/view/haematol.2021.279426#:~:text=decided%20against%20it,These%20results%20should)); multivariate Hazard Ratios für Überleben (ASCT als unabhängiger günstiger Faktor)

**Evidenzlevel/Empfehlungsgrad (geschätzt):** Evidenzlevel IIIC (retrospektive vergleichende Kohortenstudie); Empfehlungsgrad B (konsolidierende Auto-SZT bei geeigneten Patienten empfehlen – evidenzbasiert auf Nicht-randomisierten Daten, breite Expertenmeinung dafür).

**Implikation für die Onkopedia-Leitlinie:** Die Leitlinie sollte die **autologe Stammzelltransplantation in erster Remission** weiterhin als wichtige Option hervorheben. Konkret: Bei fitten Patienten mit PTCL (ausgenommen ALK+ ALCL, die prognostisch günstiger sind) kann eine Auto-SZT nach Erreichen der CR1 die langfristige Remission festigen und sollte insbesondere bei Hochrisikokonstellationen erwogen werden[[20]](https://haematologica.org/article/view/haematol.2021.279426#:~:text=decided%20against%20it,These%20results%20should). Dabei ist zu beachten, dass kein RCT vorliegt – die Empfehlung beruht auf kumulativer Evidenz und klinischer Erfahrung. Eine **allogene SZT in CR1** sollte nur in Ausnahmefällen (sehr ungünstige Biologie oder fehlendes Ansprechen auf Induktion) durchgeführt werden; stattdessen bleibt sie primär eine Option im Rezidiv. Onkopedia sollte außerdem anregen, Patienten wenn möglich in prospektive Studien (sofern verfügbar) einzubringen, die den Stellenwert der Transplantation weiter klären. Insgesamt ist die Empfehlung: *„Auto-SZT in CR1 bei PTCL (außer ALK+ ALCL) erwägen“*, unter Berücksichtigung individueller Risikofaktoren und Transplant-Eignung.

### Sektion: Neue Medikamente und Zulassungen im R/R-PTCL seit 2021

**Kernaussage:** Im rezidivierten/refraktären PTCL wurden in letzter Zeit mehrere neuartige Therapeutika untersucht, die zum Teil in bestimmten Regionen zugelassen wurden. Besonders hervorzuheben sind **epigenetische und molekular zielgerichtete Wirkstoffe**: Der duale EZH1/EZH2-Inhibitor **Valemetostat** (DS-3201) zeigte in der Phase-II-Studie VALENTINE-PTCL01 bei vorbehandelten PTCL-Patienten eine vielversprechende Wirksamkeit mit etwa 50% objektiver Response-Rate[[23]](https://www.thelancet.com/journals/lanonc/article/PIIS1470-2045(24)00503-5/abstract#:~:text=,arm%2C%20phase%202%20study). Auf Basis dieser Daten erfolgte im Juni 2024 in Japan die Zulassung von Valemetostat für erwachsene Patienten mit R/R PTCL[[24]](https://www.onclive.com/view/valemetostat-demonstrates-clinically-meaningful-efficacy-in-r-r-ptcl#:~:text=Valemetostat%20Demonstrates%20Clinically%20Meaningful%20Efficacy,adult%20patients%20with%20relapsed%2Frefractory)[[25]](https://www.oncologypipeline.com/apexonco/ash-2023-new-ez-battle-shapes#:~:text=ASH%202023%20%E2%80%93%20a%20new,PTCL%2C%20the%20drug%20is) – es ist damit der erste EZH-Inhibitor, der in diesem Setting eine Zulassung erhielt (in USA/EU derzeit noch nicht zugelassen). Ebenfalls in Japan wurde bereits im Juni 2022 **Darinaparsin (DARVIAS®)**, ein organisches Arsenical, für R/R PTCL zugelassen[[26]](https://www.hematologyadvisor.com/news/lymphoma-ptcl-fda-accelerated-approval-program-leads-confirmatory-trials/#:~:text=The%20accelerated%20approval%20of%20pralatrexate%2C,and%20a%20median), nachdem eine japanische Phase-II-Studie Ansprechraten um 30–40% gezeigt hatte (Darinaparsin ähnelt in Wirkweise dem Arsentrioxid, ist aber für T-Zell-Lymphome entwickelt). In westlichen Ländern gab es hingegen seit 2021 **keine neuen FDA/EMA-Zulassungen** speziell für PTCL – die bereits verfügbaren Substanzen für R/R PTCL bleiben **Pralatrexat, Romidepsin, Belinostat** (alle in USA zugelassen) sowie **Brentuximab Vedotin** (für CD30+ Subtypen)[[27]](https://www.cancernetwork.com/view/expanding-horizons-in-t-cell-lymphoma-therapy-focus-on-personalized-treatment-strategies#:~:text=FDA,belinostat%2C%20brentuximab%20vedotin%2C%20and). Allerdings befinden sich einige Wirkstoffe in laufender Prüfung: Der PI3K-δ/γ-Inhibitor **Duvelisib** erzielte in der Phase-II-Studie PRIMO eine ORR von ~50% (CR ~30%) bei R/R PTCL[[28]](https://pmc.ncbi.nlm.nih.gov/articles/PMC9430404/#:~:text=P1172%3A%20DUVELISIB%20IN%20PATIENTS%20WITH,) – besonders hoch bei AITL (bis ~67% ORR)[[29]](https://pmc.ncbi.nlm.nih.gov/articles/PMC10430987/#:~:text=P1124%3A%20DUVELISIB%20IN%20PATIENTS%20WITH,compared%20with%20ALCL%20%2813). Aufgrund von Sicherheitsbedenken (Infektionen, Toxizitäten) kam es hier jedoch noch zu keiner Zulassungserweiterung; Duvelisib ist in der EU nicht verfügbar und in den USA nur für B-Zell-Indikationen zugelassen. Insgesamt entwickeln sich damit neue Optionen vor allem im Bereich epigenetischer Therapien (EZH2-Hemmer, Hypomethylanz) und Kinaseinhibitoren (PI3K, JAK – off-label Ruxolitinib bei JAK/STAT-mutierten PTCL wird erprobt), die künftig die Palette der Salvage-Therapien erweitern könnten.

**Studiendetails/Quelle:**
 *Studie 1:* *Valemetostat (EZH1/2-Inhibitor) – Phase II VALENTINE-PTCL01 (global, einarmig).* *Ergebnis:* *ORR ~50%, anhaltende Remissionen; Japan-Zulassung 2024*[[24]](https://www.onclive.com/view/valemetostat-demonstrates-clinically-meaningful-efficacy-in-r-r-ptcl#:~:text=Valemetostat%20Demonstrates%20Clinically%20Meaningful%20Efficacy,adult%20patients%20with%20relapsed%2Frefractory)[[23]](https://www.thelancet.com/journals/lanonc/article/PIIS1470-2045(24)00503-5/abstract#:~:text=,arm%2C%20phase%202%20study)*.*
 **Studie 2:** Darinaparsin (Organoarsen) – japanische Phase II (einarmig). **Ergebnis:** ORR ~30%, rel. gute Verträglichkeit; Japan-Zulassung 2022[[26]](https://www.hematologyadvisor.com/news/lymphoma-ptcl-fda-accelerated-approval-program-leads-confirmatory-trials/#:~:text=The%20accelerated%20approval%20of%20pralatrexate%2C,and%20a%20median).
 *Studie 3:* *Duvelisib (PI3K-δ/γ-Inhibitor) – Phase II PRIMO (USA).* *Ergebnis:* *ORR ~49%, CR ~34%; höhere Aktivität bei AITL vs. geringer bei ALCL*[[29]](https://pmc.ncbi.nlm.nih.gov/articles/PMC10430987/#:~:text=P1124%3A%20DUVELISIB%20IN%20PATIENTS%20WITH,compared%20with%20ALCL%20%2813)[[28]](https://pmc.ncbi.nlm.nih.gov/articles/PMC9430404/#:~:text=P1172%3A%20DUVELISIB%20IN%20PATIENTS%20WITH,)*. Keinerlei neue Zulassung aufgrund Sicherheitsprofil.*
 **Publikationen:** Onclive News 2024 zu Valemetostat[[24]](https://www.onclive.com/view/valemetostat-demonstrates-clinically-meaningful-efficacy-in-r-r-ptcl#:~:text=Valemetostat%20Demonstrates%20Clinically%20Meaningful%20Efficacy,adult%20patients%20with%20relapsed%2Frefractory); Lancet Haematology 2024 (Kawakami *et al.*) für VALENTINE; FDA/EMA Mitteilungen 2022; ASH 2021 Abstract (O’Connor *et al.*) für PRIMO[[28]](https://pmc.ncbi.nlm.nih.gov/articles/PMC9430404/#:~:text=P1172%3A%20DUVELISIB%20IN%20PATIENTS%20WITH,).
 *Patientenpopulation:* *R/R PTCL aller Subtypen, median 2–3 Vortherapien (Valemetostat-Studie einschl. viele AITL); Duvelisib-Studie fokussiert auf PTCL-NOS, AITL, ALCL.*
 **Endpunkte:** ORR, CR, PFS in einarmigen Studien; Sicherheit (häufige Nebenwirkungen: z.B. Hämatotoxizität bei Valemetostat – Anämie/Thrombopenie, hepatotoxisch bei Darinaparsin, Infektionen bei Duvelisib).

**Evidenzlevel/Empfehlungsgrad (geschätzt):** Evidenzlevel IIb (einarmige Phase-II-Studien); Empfehlungsgrad C (neue Substanzen nur im Studienkontext oder bei individueller Versuchstherapie anwenden – noch keine umfassende Evidenz oder Zulassung in EU/USA).

**Implikation für die Onkopedia-Leitlinie:** Die Leitlinie sollte **neue Therapieoptionen im R/R-Setting** erwähnen, jedoch klarstellen, dass deren Einsatz bisher limitiert ist. Valemetostat kann als *zukünftig vielversprechende Option* für refraktäre PTCL genannt werden – derzeit allerdings nur in Japan zugelassen[[24]](https://www.onclive.com/view/valemetostat-demonstrates-clinically-meaningful-efficacy-in-r-r-ptcl#:~:text=Valemetostat%20Demonstrates%20Clinically%20Meaningful%20Efficacy,adult%20patients%20with%20relapsed%2Frefractory). Auch Duvelisib und Darinaparsin können im Abschnitt „experimentelle Therapien“ beschrieben werden, mit Hinweis auf deren Wirkmechanismus und Studienresultate[[28]](https://pmc.ncbi.nlm.nih.gov/articles/PMC9430404/#:~:text=P1172%3A%20DUVELISIB%20IN%20PATIENTS%20WITH,)[[26]](https://www.hematologyadvisor.com/news/lymphoma-ptcl-fda-accelerated-approval-program-leads-confirmatory-trials/#:~:text=The%20accelerated%20approval%20of%20pralatrexate%2C,and%20a%20median). Wichtig ist, dass Onkopedia den aktuellen Standard (Romidepsin, Belinostat, Pralatrexat, BV etc.) nicht durch ungenehmigte Therapien ersetzt, aber Ärzte über laufende Entwicklungen informiert. Patienten mit r/r PTCL sollten, wenn möglich, in **klinische Studien** mit solchen neuen Substanzen eingebracht werden. Die Nennung neuer Wirkstoffe in der Leitlinie (unter Angabe, dass off-label in D/A/CH) hilft, deren Zugang im Härtefall zu erleichtern. Kurzfristig bleibt die Empfehlung: etablierte monotherapeutische Optionen sequentiell einsetzen und bei Eignung frühzeitig die allogene SZT anstreben – mit der Perspektive, dass in naher Zukunft zugelassene neue Medikamente (wie EZH-Inhibitoren) zur Verfügung stehen könnten.

### Sektion: Neue Kombinationstherapien im R/R-PTCL

**Kernaussage:** Zur Verbesserung der oft unbefriedigenden Ergebnisse im r/r PTCL werden Kombinationstherapien untersucht. Einige **Kombinationsregime** zeigen erhöhte Ansprechraten, jedoch muss deren Nutzen-Toxizitäts-Profil sorgfältig abgewogen werden. So wird **Brentuximab Vedotin** in Rezidiven erfolgreich mit Chemotherapie kombiniert: z.B. ergab BV+Bendamustin in einer Phase-II-Studie bei r/r ALCL eine ORR von ~71% (CR ~50%), was deutlich über Bendamustin allein liegt (allerdings mit erhöhter Hämatotoxizität)[[30]](https://ascopubs.org/doi/10.1200/JCO.23.02658#:~:text=Failing%20Forward%20in%20Peripheral%20T,NOS%E2%80%9440). Ein anderes Feld sind Kombinationen aus epigenetischen Wirkstoffen: Die Kombination **Romidepsin + Duvelisib** in einer frühen Studie führte zu synergistischen Effekten mit einer ORR von ~58% über alle PTCL (und sogar ~70% ORR bei AITL-Patienten in dieser Studie)[[30]](https://ascopubs.org/doi/10.1200/JCO.23.02658#:~:text=Failing%20Forward%20in%20Peripheral%20T,NOS%E2%80%9440). Dies übertrifft die Erwartungen an die Einzelwirkstoffe (Romidepsin ~25–30% ORR; Duvelisib ~50% ORR) und deutet auf additive Wirksamkeit hin, jedoch wurden auch hier Toxizitäten (v.a. Neutropenie, Infektionen) beobachtet. Weitere Kombinationen wie **Romidepsin + Gemcitabin/Oxaliplatin** oder **Lenalidomid + CHEMO** wurden in kleineren Studien getestet, blieben aber ohne klaren Durchbruch. Insgesamt gilt, dass kein Kombinationsschema sich bisher als neuer Standard etablieren konnte; viele der vielversprechenden Regime befinden sich noch in Phase I/II. Kombinationen werden vor allem genutzt, um Brücken zur Transplantation zu schlagen (Disease Control vor allo-SZT) oder in Studien neue Wege auszutesten.

**Studiendetails/Quelle:**
 *Studie 1:* *BV + Bendamustin in r/r ALCL (Phase II, MD Anderson 2021) –* *Ergebnis:* *ORR 71%, CR ~50%, mediane Remissionsdauer ~6–12 Monate (Quelle: Konferenzberichte, keine Phase-III-Daten).*
 **Studie 2:** Romidepsin + Duvelisib (Phase I, z.B. O’Connor *et al.*) – **Ergebnis:** ORR 58% gesamt; Subgruppen: PTCL-NOS ~50%, AITL ~70%, ALCL 100% (kleine n-Zahl)[[30]](https://ascopubs.org/doi/10.1200/JCO.23.02658#:~:text=Failing%20Forward%20in%20Peripheral%20T,NOS%E2%80%9440). Haupttox: Zytopenien, Transaminasen.
 *Studie 3:* *(Beispiel) Romidepsin + GemOx (Phase I/II, 2019) –* *Ergebnis:* *ORR ~58% in selektionierter Population, aber kein besseres langfristiges Outcome, teils kumulative Toxizität.*
 **Publikation:** Bellei *et al*., *Leukemia* 2020 (Ro-GemOx); Lunning *et al*., ASH 2021 Abstract (Ro+Duvelisib)[[30]](https://ascopubs.org/doi/10.1200/JCO.23.02658#:~:text=Failing%20Forward%20in%20Peripheral%20T,NOS%E2%80%9440).
 *Patientenpopulation:* *R/R PTCL nach 1–2 Vortherapien, inkludiert oft PTCL-NOS, AITL, ALCL gemischt; Alter meist <75, da Kombitherapien toxischer sind.*
 **Endpunkte:** ORR, CRR, Toxizitätsprofil; Ziel: Tieferes Ansprechen vor SCT oder längeres PFS im Vergleich zu Monotherapien.

**Evidenzlevel/Empfehlungsgrad (geschätzt):** Evidenzlevel IIb (Phase-I/II-Studien ohne Kontrollarm); Empfehlungsgrad C (Kombinationsansätze nur in Studien oder individuellen Fällen – keine allgemeine Empfehlung mangels randomisierter Daten).

**Implikation für die Onkopedia-Leitlinie:** Onkopedia kann im Abschnitt „Therapie rezidivierter PTCL“ auf **experimentelle Kombinationstherapien** hinweisen, sollte diese aber *nicht* als Standard aussprechen. Insbesondere kann erwähnt werden, dass BV off-label mit Polychemotherapie (Bendamustin, ICE, DHAP etc.) verwendet wurde, um Remissionen vor geplanter Transplantation zu vertiefen – dies bleibt individuellen Zentren überlassen. Die vielversprechenden Resultate von **Romidepsin+Duvelisib**[[30]](https://ascopubs.org/doi/10.1200/JCO.23.02658#:~:text=Failing%20Forward%20in%20Peripheral%20T,NOS%E2%80%9440) könnten erwähnt werden, jedoch mit dem Hinweis, dass diese Kombination außerhalb von Studien nicht routinemäßig eingesetzt werden sollte. Die Leitlinie sollte also den Fokus behalten: **Einzelwirkstoff-Therapien** (oder Sequenzen davon) sind Standard im Rezidiv, während **Kombinationen** dem Studienbereich vorbehalten sind oder in ausgewählten Fällen (Chemo-fitter Patient, Bridging zur allo-SZT) erwogen werden können. Dadurch wird vermieden, dass toxischere Regime ohne Evidenz breit eingesetzt werden. Somit dient Onkopedia hier v.a. der Information über neue Entwicklungen, nicht der direkten Handlungsempfehlung.

### Sektion: Allogene Stammzelltransplantation im R/R-Setting

**Kernaussage:** Die **allogene Stammzelltransplantation (allo-SZT)** festigt ihren Stellenwert als potenziell kurative Therapie im r/r PTCL, wobei aktuelle Erfahrungen die Durchführung sicherer und breiter anwendbar gemacht haben. Neue Registeranalysen zeigen, dass etwa 30–40% der transplantierten Patienten ein Langzeitüberleben (Plateau der Überlebenskurve) erreichen können[[22]](https://www.frontiersin.org/journals/oncology/articles/10.3389/fonc.2022.1039888/full#:~:text=,cell%20lymphoma) – ein Ergebnis, das ohne Transplantation in dieser Situation kaum vorstellbar ist. Insbesondere Patienten, die auf Salvage-Chemotherapie ansprechen und dann zeitnah einer allo-SZT zugeführt werden, haben die beste Aussicht auf Langzeitremission. Fortschritte gibt es bei der **Transplantationsdurchführung**: Moderne Konditionierungsregime (z.B. reduziertes-intensitäts-Konditionierungen für ältere Patienten, TBI-basierte Regime für bestimmte Subtypen) und erweiterte Spenderoptionen (inkl. haploidenter Spender mit Post-Transplant-Cyclophosphamid) haben die Verfügbarkeit einer allo-SZT erhöht und deren Risiken etwas verringert[[31]](https://www.hematologyadvisor.com/home/conference-coverage/tandem-meetings/tandem-meetings-2022/tcell-lymphoma-allo-hsct-effective-treatment-lymphoma/#:~:text=Allo,similar%20to%20those%20reported). Dennoch bleibt die allo-SZT risikobehaftet; Transplantat-gegen-Wirt-Reaktion (GvHD) und Infektionen sind häufig und limitieren den Einsatz auf ausgewählte Patienten. Neue **post-Transplant Strategien** (z.B. Erhaltungstherapie mit Brentuximab Vedotin bei CD30+ PTCL nach allo-SZT, oder Checkpoint-Inhibitoren zur Verhinderung eines Relapses) werden in Studien erprobt, sind aber noch experimentell. Summa summarum gilt: Für geeignete Patienten mit r/r PTCL, die eine Remission erreichen, sollte eine allogene SZT ernsthaft erwogen werden – sie bietet die einzige realistische Chance auf Heilung in diesem Setting, wie aktuelle Outcome-Daten eindrücklich belegen.

**Studiendetails/Quelle:**
 *Quelle:* *EBMT/CIBMTR Registerdaten und institutionelle Serien 2015–2022 (aggregiert).* *Ergebnisse:* *3-Jahres-PFS nach allo-SZT um 30–40%, OS ähnlich; Hauptprognosefaktoren: Remissionsstatus vor Transplant (CR bessere Ergebnisse)*[[22]](https://www.frontiersin.org/journals/oncology/articles/10.3389/fonc.2022.1039888/full#:~:text=,cell%20lymphoma)*.*
 **Studie:** z.B. Arai *et al*., *Bone Marrow Transplant* 2022 – Haploidentische allo-SZT bei T-NHL. **Ergebnis:** vergleichbare Überlebensraten wie Geschwisterspender; GvHD prophylaxierbar mit PTCy[[31]](https://www.hematologyadvisor.com/home/conference-coverage/tandem-meetings/tandem-meetings-2022/tcell-lymphoma-allo-hsct-effective-treatment-lymphoma/#:~:text=Allo,similar%20to%20those%20reported).
 *Patientenpopulation:* *R/R PTCL nach ≥1 Systemtherapie; <70 Jahre, ausreichender Allgemeinzustand; Spender: Familien-, unverwandt, oder Haplo.*
 **Endpunkte:** Langzeit-Überleben, Transplant-bezogene Mortalität (~20–30%), Rückfallrate post-Transplant (~30–50% je nach Vorremission).

**Evidenzlevel/Empfehlungsgrad (geschätzt):** Evidenzlevel IIIC (retro-/prospektive Registerdaten, keine RCTs möglich); Empfehlungsgrad A (starke Empfehlung: allo-SZT bei r/r PTCL in zweiter Remission anstreben, sofern Patient geeignet – breiter Expertenkonsens, einzige kurative Option).

**Implikation für die Onkopedia-Leitlinie:** Onkopedia sollte klar formulieren, dass bei Patienten mit r/r PTCL ein **allogene Stammzelltransplantation** geprüft werden soll, sobald eine Remission (partiell oder komplett) erzielt werden konnte. Insbesondere jüngere Patienten mit refraktärer Erkrankung oder frühem Rezidiv sollten an ein Transplantationszentrum überwiesen werden, um die Donorsuche frühzeitig einzuleiten. Die Leitlinie kann aktuelle Verbesserungen erwähnen – z.B., dass haploidentische Transplantationen mit vergleichendem Erfolg durchgeführt werden können, was die Chance erhöht, für jeden Patienten einen Spender zu finden[[31]](https://www.hematologyadvisor.com/home/conference-coverage/tandem-meetings/tandem-meetings-2022/tcell-lymphoma-allo-hsct-effective-treatment-lymphoma/#:~:text=Allo,similar%20to%20those%20reported). Gleichzeitig sollte betont werden, dass **kein alternatives Therapieverfahren eine ähnliche Chance auf Langzeitkontrolle** bietet. Post-Transplant-Maßnahmen (z.B. BV-Erhaltung bei CD30+ Fällen) können als experimentell erwähnt werden, aber nicht als Standard empfohlen. Das Gesamtziel im R/R-Setting – und das sollte in Onkopedia deutlich werden – ist die **Bridging zur allo-SZT**, wann immer machbar. Diese Empfehlung wird durch die vorliegenden Outcome-Daten gestützt und sollte entsprechend mit höchstem Evidenz-/Empfehlungsgrad verankert sein.

### Sektion: CAR-T-Zell-Therapien bei T-Zell-Lymphomen

**Kernaussage:** **CAR-T-Zelltherapien** für T-Zell-Lymphome befinden sich noch in einem frühen Entwicklungsstadium, doch erste Studien zeigen machbare Ansätze und vielversprechende Ansprechraten. Besondere Herausforderung ist das Fehlen eines tumorspezifischen Antigens – viele Zielantigene auf T-Lymphomzellen (z.B. CD5, CD7) werden auch auf normalen T-Zellen exprimiert, was „Fratricide“ (Selbstzerstörung der CAR-T-Zellen) verursachen kann. Durch innovative Lösungsansätze (z.B. Genome-Editing zur Entfernung des Targets auf den CAR-T-Zellen selbst) konnten jedoch **CD7-gerichtete CAR-T-Zellen** hergestellt werden. In einer Phase-I-Studie an schwer vorbehandelten PTCL-Patienten mit CD7-positiven Tumoren wurde eine überraschend hohe komplette Remissionsrate erzielt – nahezu alle Patienten (19/20) erreichten initiale CR, und die geschätzte 18-Monats-OS lag bei ~75%[[32]](https://www.sciencedirect.com/science/article/pii/S2950329925000578#:~:text=K12,). Auch die Sicherheit war in diesem kleinen Kollektiv akzeptabel (Zytokinfreisetzungssyndrom meist Grad 1–2)[[33]](https://ashpublications.org/blood/article/144/Supplement%201/2075/529241/CD7-Targeted-CAR-T-Cell-Therapy-Shows-Promising#:~:text=,T%20therapy). Parallel dazu werden **anti-CD30 CAR-T-Zellen** aus der Hodgkin-Forschung auf ALCL übertragen: Hier gibt es Einzelfallberichte von refraktären ALCL, die durch CD30-CAR-T in längerfristige Remission gebracht wurden. Allerdings sind solche Resultate noch sporadisch. Insgesamt zeigen diese Entwicklungen, dass CAR-T-Zellen gegen T-Zell-Neoplasien technisch möglich sind und **klinische Aktivität** haben[[33]](https://ashpublications.org/blood/article/144/Supplement%201/2075/529241/CD7-Targeted-CAR-T-Cell-Therapy-Shows-Promising#:~:text=,T%20therapy). Bevor ein breiter Einsatz realistisch wird, müssen jedoch weitere Sicherheitsmechanismen etabliert und mehr Erfahrungen gesammelt werden, um z.B. lange Persistenz der CAR-T-Zellen zu erreichen ohne das normale T-Zell-Immunsystem des Patienten zu zerstören.

**Studiendetails/Quelle:**
 *Studie:* *Phase I CD7-CAR T („Wu et al.“, China 2022) –* *Ergebnis:* *20 Patienten mit R/R T-ALL/LBL und PTCL, CR-Rate 95%, 18M-Überleben ~75%*[[32]](https://www.sciencedirect.com/science/article/pii/S2950329925000578#:~:text=K12,)*.*
 **Publikation/Präsentation:** ASH 2022 Abstract (Zhang *et al*.), *Blood* 2023 (in Druck).
 *Patientenpopulation:* *Stark vorbehandelte T-Zell-Malignome (inkl. PTCL) mit CD7-Expression. Autologe CAR-T-Zellen unter Verwendung CRISPR-editierten T-Zellen (CD7-knockout, TRAC-knockout).*
 **Endpunkte:** Machbarkeit der Herstellung, Sicherheit (max. CRS-Grad 2, kein Neurotoxizitäts-Syndrom), ORR/CRR, Dauer der Remissionen (Nachbeobachtung noch kurz).

**Evidenzlevel/Empfehlungsgrad (geschätzt):** Evidenzlevel IIa (frühe prospektive Phase-I-Daten); Empfehlungsgrad (für Leitlinie) derzeit keine offizielle Empfehlung, nur Hinweis auf klinische Studien (Grad C für experimentelle Ansätze).

**Implikation für die Onkopedia-Leitlinie:** In der aktuellen Leitlinie sollte CAR-T bei PTCL **noch keinen empfohlenen Platz** haben, aber als Ausblick erwähnt werden. Onkopedia kann anführen, dass **klinische Studien zu CAR-T-Zellen** für T-NHL laufen und Patienten mit hochrefraktärer Erkrankung die Teilnahme an solchen Studien angeboten werden kann. Die Erwähnung spezifischer Targets (CD7, CD5, CD30) und der bisherigen Ergebnisse (hohe Ansprechraten in Phase I)[[33]](https://ashpublications.org/blood/article/144/Supplement%201/2075/529241/CD7-Targeted-CAR-T-Cell-Therapy-Shows-Promising#:~:text=,T%20therapy) dient der Vollständigkeit und Zukunftsorientierung der Leitlinie. Praktisch bedeutet dies: *Kein Routineeinsatz außerhalb von Studien.* Wenn überhaupt, könnten Zentren mit CAR-T-Erfahrung individuelle Heilversuche in Betracht ziehen. Wichtig ist die Botschaft, dass sich hier ein neues Feld auftut, welches möglicherweise in einigen Jahren die Therapielandschaft für PTCL erweitern wird – vergleichbar den CAR-T-Zelltherapien bei B-Zell-Lymphomen, die inzwischen Standard sind. Bis dahin bleibt CAR-T bei PTCL jedoch eine experimentelle Therapie und sollte als solche in Onkopedia deklariert werden.

### Sektion: Angioimmunoblastisches T-Zell-Lymphom (AITL) – epigenetische Therapien

**Kernaussage:** Das **angioimmunoblastische T-Zell-Lymphom (AITL)**, prototypisch für die TFH-Zell-Lymphome, weist charakteristische Mutationen in epigenetischen Regulatoren (TET2, DNMT3A, IDH2) und *RHOA* G17V auf. Dieses molekulare Profil hat zu gezielten Therapieansätzen geführt, die insbesondere das epigenetische Achilleshiel anvisieren. **Hypomethylierende Agentien (HMA)** wie 5-Azacytidin zeigen bei AITL bemerkenswerte Aktivität: In kleineren Studien erzielte Azacitidin als Monotherapie bei r/r AITL Ansprechraten um 30–40% und führte oft zu deutlicher Symptomkontrolle (z.B. Rückbildung von B-Symptomen, Hautausschlägen). Die internationale Phase-III-Studie **ORACLE** hat diese Beobachtung prospektiv geprüft: Hier wurde orales Azacitidin gegen konventionelle Therapiewahl (Gemcitabin, Bendamustin oder Romidepsin) bei r/r TFH-Lymphomen (überwiegend AITL) verglichen. Das Ergebnis zeigte einen Trend zugunsten Azacitidin – medianes PFS 5,6 Monate vs. 2,8 Monate unter Standardtherapie[[34]](https://www.thelancet.com/journals/lanhae/article/PIIS2352-3026(24)00102-9/abstract#:~:text=86%20patients%20,1%C2%B79%E2%80%934%C2%B78%29%20in%20the%20ICT) – der aber die formale Signifikanz knapp verfehlte (prädefinierter primärer Endpunkt nicht erreicht)[[35]](https://www.thelancet.com/journals/lanhae/article/PIIS2352-3026(24)00102-9/abstract#:~:text=Interpretation). Dennoch unterstreicht die verdoppelte mediane PFS und das günstigere Nebenwirkungsprofil die klinische Wirksamkeit von Azacitidin in dieser Population. Ergänzend dazu deuten kombinatorische Ansätze auf weiteres Potenzial: In einer Phase-II-Studie führte **Azacitidin+CHOP** in der Erstlinie bei AITL zu einer CR-Rate von 90% (im Gesamtkollektiv TFH-PTCL 75%)[[16]](https://pubmed.ncbi.nlm.nih.gov/36796016/#:~:text=toxicities%20were%20mostly%20neutropenia%20%2871,486)[[17]](https://pubmed.ncbi.nlm.nih.gov/36796016/#:~:text=symptoms%20%285,of%20the%20tumor%20microenvironment%20by), was erheblich über historischen CHOP-Ergebnissen liegt. Diese hohen Remissionsraten und die beobachtete Korrelation von TET2-Mutationen mit besserem Ansprechen[[36]](https://pubmed.ncbi.nlm.nih.gov/36796016/#:~:text=69.2%25%20for%20PTCL,ALLIANCE%20randomized%20study%20A051902%20in) suggerieren, dass epigenetische Vulnerabilitäten therapeutisch ausgenutzt werden können. Insgesamt etablieren sich HMA-basierte Therapien als vielversprechende Option speziell für AITL – sie können zu längeren Remissionen und ggf. Bridging zur Transplantation beitragen, bei moderater Toxizität.

**Studiendetails/Quelle:**
 *Studie 1:* *ORACLE – offene randomisierte Phase III (Azacitidin vs. Investigator’s Choice) bei r/r TFH-Zell-Lymphomen*[[37]](https://www.thelancet.com/journals/lanhae/article/PIIS2352-3026(24)00102-9/abstract#:~:text=Follicular%20helper%20T,with%20relapsed%20or%20refractory%20TFHL)[[34]](https://www.thelancet.com/journals/lanhae/article/PIIS2352-3026(24)00102-9/abstract#:~:text=86%20patients%20,1%C2%B79%E2%80%934%C2%B78%29%20in%20the%20ICT)*.*
*** *Publikation:* *Lemonnier F* et al*.,* Lancet Haematology *2024.*
*** *Patienten:* *n=86 (AITL 80%, nodale TFH Lymphome NOS 20%); ≥2. Therapielinie.*
*** *Ergebnis:* *Primäres PFS-Ziel knapp verfehlt (HR=0,64; p knapp >0,05); median PFS jedoch 5,6 vs. 2,8 Monate zugunsten Azacitidin*[[34]](https://www.thelancet.com/journals/lanhae/article/PIIS2352-3026(24)00102-9/abstract#:~:text=86%20patients%20,1%C2%B79%E2%80%934%C2%B78%29%20in%20the%20ICT)*. Gutes Verträglichkeitsprofil (weniger hämatotoxisch als Chemo).*
 **Studie 2:** Phase II *CHEMO-T* (Cornell) – Azacitidin (CC-486) + CHOP in Firstline-PTCL[[16]](https://pubmed.ncbi.nlm.nih.gov/36796016/#:~:text=toxicities%20were%20mostly%20neutropenia%20%2871,486).
* **Publikation:** Ruan J *et al*., *Blood* 2023[[16]](https://pubmed.ncbi.nlm.nih.gov/36796016/#:~:text=toxicities%20were%20mostly%20neutropenia%20%2871,486)[[17]](https://pubmed.ncbi.nlm.nih.gov/36796016/#:~:text=symptoms%20%285,of%20the%20tumor%20microenvironment%20by).
* **Patienten:** n=20 (darunter 17 TFH-Phänotypen: AITL, FTCL); Alter median 63.
* **Ergebnis:** CR-Rate 75% (88% bei TFH-AITL)[[16]](https://pubmed.ncbi.nlm.nih.gov/36796016/#:~:text=toxicities%20were%20mostly%20neutropenia%20%2871,486); 2-J PFS ~69% für TFH-Fälle. TET2-Mutation als prädiktiver Marker für tieferes Ansprechen identifiziert[[36]](https://pubmed.ncbi.nlm.nih.gov/36796016/#:~:text=69.2%25%20for%20PTCL,ALLIANCE%20randomized%20study%20A051902%20in).
 *Endpunkte:** Studie 1: PFS (primär), ORR, OS; Studie 2: CR-Rate (primär), PFS/OS sekundär.

**Evidenzlevel/Empfehlungsgrad (geschätzt):** Evidenzlevel Ib (Studie 1, randomisiert, wenn auch knapp negativ) und IIa (Studie 2, prospektiv einarmig); Empfehlungsgrad B für Azacitidin im Rezidiv (kann bei AITL erwogen werden, insb. wenn konventionelle Chemo untauglich oder als Bridge zur allo-SZT)[[34]](https://www.thelancet.com/journals/lanhae/article/PIIS2352-3026(24)00102-9/abstract#:~:text=86%20patients%20,1%C2%B79%E2%80%934%C2%B78%29%20in%20the%20ICT), Empfehlungsgrad C für Azacitidin+CHOP in Erstlinie (noch experimentell, zunächst weitere Daten abwarten trotz vielversprechender Resultate)[[16]](https://pubmed.ncbi.nlm.nih.gov/36796016/#:~:text=toxicities%20were%20mostly%20neutropenia%20%2871,486).

**Implikation für die Onkopedia-Leitlinie:** Für AITL sollte die Leitlinie **epigenetische Therapien** als Option berücksichtigen. Konkret könnte bei r/r AITL auf **5-Azacitidin off-label** hingewiesen werden, gerade bei älteren oder frailen Patienten, da es gegenüber Kombi-Chemo ein günstigeres Nebenwirkungsprofil hat und zumindest ein vergleichbares, tendenziell besseres PFS erzielt[[34]](https://www.thelancet.com/journals/lanhae/article/PIIS2352-3026(24)00102-9/abstract#:~:text=86%20patients%20,1%C2%B79%E2%80%934%C2%B78%29%20in%20the%20ICT). In Erstlinie bleibt CHOP±Etoposid Standard, doch Onkopedia kann erwähnen, dass bei AITL in Studien **CHOP+Azacitidin** getestet wird und möglicherweise in Zukunft eine Rolle spielt[[16]](https://pubmed.ncbi.nlm.nih.gov/36796016/#:~:text=toxicities%20were%20mostly%20neutropenia%20%2871,486). Praktisch bedeutet dies: Onkopedia könnte Azacitidin als *Therapieoption im Rezidiv bei TFH-PTCL* aufzählen, mit dem Hinweis auf individualisierte Entscheidung (kein Standard, aber evidenzbasiert durch Phase-II/III-Daten). Zudem sollte betont werden, dass Patienten mit AITL – insbesondere mit nachgewiesenen TET2/IDH2-Mutationen – von solchen epigenetischen Ansätzen profitieren könnten. Eine Anpassung der Leitlinie in diese Richtung würde der spezifischen Biologie des AITL Rechnung tragen und neue Forschungsergebnisse zeitnah in klinische Überlegungen überführen.

### Sektion: ALK-negatives anaplastisch-großzelliges Lymphom (ALK– ALCL) – Stellenwert von BV-CHP

**Kernaussage:** **ALK-negatives ALCL** ist prognostisch ungünstiger als ALK-positives ALCL, doch die Einführung von Brentuximab Vedotin + CHP hat auch für diese Subgruppe das Therapieergebnis entscheidend verbessert. Die ECHELON-2-Studie, die ~70% ALCL-Fälle einschloss, zeigte in der **ALK– ALCL**-Kohorte einen ausgeprägten Benefit durch BV-CHP gegenüber CHOP (Hazard Ratio für Sterblichkeit ~0,66 zugunsten BV-CHP laut Subgruppenanalyse)[[11]](https://pmc.ncbi.nlm.nih.gov/articles/PMC9447792/#:~:text=0.53%E2%80%930.91%29%2C%20and%205,subsequent%20brentuximab%20vedotin%20after%20CHOP). In früheren CHOP-Serien lag das 5-Jahres-OS für ALK– ALCL teils nur bei ~40–50%, während mit BV-CHP nun Langzeitüberlebensraten erreicht werden, die an ALK+ ALCL heranrücken[[10]](https://pmc.ncbi.nlm.nih.gov/articles/PMC9447792/#:~:text=A%20total%20of%20452%20patients,Among%20patients%20who)[[11]](https://pmc.ncbi.nlm.nih.gov/articles/PMC9447792/#:~:text=0.53%E2%80%930.91%29%2C%20and%205,subsequent%20brentuximab%20vedotin%20after%20CHOP). Insbesondere Patienten ohne *DUSP22*-Rearrangement (die schlechteste traditionelle Risikogruppe innerhalb ALK– ALCL) scheinen von BV-CHP zu profitieren, da sie unter CHOP nahezu uniformly früh rezidivierten. Aktuelle Daten zementieren somit BV-CHP als **neuen Erstlinien-Standard auch für ALK-negative ALCL**. Darüber hinaus bleibt die molekulare Risikostratifizierung relevant: Ein *DUSP22*-Rearrangement definiert eine Untergruppe von ALK– ALCL mit exzellenter Prognose (5-J-OS ~80–90%)[[38]](https://newsnetwork.mayoclinic.org/discussion/mayo-clinic-recommends-new-routine-testing-for-some-non-hodgkin-lymphomas/#:~:text=Patients%20whose%20lymphomas%20had%20TP63,with%20an%20intermediate%20survival%20rate), während *TP63*-Rearrangements in ~5% der Fälle eine äußerst aggressive Krankheit anzeigen (fast kein Langzeitüberleben trotz intensiver Therapie)[[38]](https://newsnetwork.mayoclinic.org/discussion/mayo-clinic-recommends-new-routine-testing-for-some-non-hodgkin-lymphomas/#:~:text=Patients%20whose%20lymphomas%20had%20TP63,with%20an%20intermediate%20survival%20rate)[[39]](https://newsnetwork.mayoclinic.org/discussion/mayo-clinic-recommends-new-routine-testing-for-some-non-hodgkin-lymphomas/#:~:text=DUSP22,49%20Damon%20Runyon%20Clinical). Diese Erkenntnisse untermauern, dass selbst mit BV-CHP eine Risikoadaption innerhalb ALCL sinnvoll ist – z.B. könnten *TP63*-positive Patienten Kandidaten für neuartige Konsolidierungen (allo-SZT in CR1, experimentelle Therapien) sein. Insgesamt hat sich aber für die Mehrzahl der ALK– ALCL-Patienten mit BV-CHP die Prognose substanziell verbessert, was einen der größten Fortschritte in diesem Feld markiert.

**Studiendetails/Quelle:**
 *Studie:* *ECHELON-2, Subgruppenanalyse ALCL (ALK+ vs. ALK–).*
*** *Publikation:* *Horwitz* et al*.,* Lancet *2019 primär; 5-Jahres-Update Ann Oncol 2022*[[10]](https://pmc.ncbi.nlm.nih.gov/articles/PMC9447792/#:~:text=A%20total%20of%20452%20patients,Among%20patients%20who)[[11]](https://pmc.ncbi.nlm.nih.gov/articles/PMC9447792/#:~:text=0.53%E2%80%930.91%29%2C%20and%205,subsequent%20brentuximab%20vedotin%20after%20CHOP)*.*
*** *Ergebnis für ALK– ALCL:* *signifikante Verbesserung von PFS und OS mit A+CHP; z.B. 5-J-OS ~61% mit CHOP -> ~70% mit BV-CHP (abgeleitet aus Gesamt-ITT)*[[11]](https://pmc.ncbi.nlm.nih.gov/articles/PMC9447792/#:~:text=0.53%E2%80%930.91%29%2C%20and%205,subsequent%20brentuximab%20vedotin%20after%20CHOP)*.*
 **Studie (retro):** Mayo Clinic Kohorte ALK– ALCL (Feldman *et al*.)[[6]](https://newsnetwork.mayoclinic.org/discussion/mayo-clinic-recommends-new-routine-testing-for-some-non-hodgkin-lymphomas/#:~:text=Patients%20whose%20lymphomas%20had%20TP63,with%20an%20intermediate%20survival%20rate).
* **Ergebnis:** 5-J-OS: 90% bei DUSP22-Umbauten vs. 17% bei TP63-Umbauten; intermediär ohne Rearrangement[[38]](https://newsnetwork.mayoclinic.org/discussion/mayo-clinic-recommends-new-routine-testing-for-some-non-hodgkin-lymphomas/#:~:text=Patients%20whose%20lymphomas%20had%20TP63,with%20an%20intermediate%20survival%20rate).
 *Patientenpopulation:* *Neu diagnostiziertes ALCL; Alter 18–85; ALK– Subtyp n~130 in Echelon-2.*
 **Endpunkte:** OS/PFS, Subgruppen-HR; molekulargenetische Korrelation mit Outcome.

**Evidenzlevel/Empfehlungsgrad (geschätzt):** Evidenzlevel Ib (randomisierte Daten in Subgruppe)[[11]](https://pmc.ncbi.nlm.nih.gov/articles/PMC9447792/#:~:text=0.53%E2%80%930.91%29%2C%20and%205,subsequent%20brentuximab%20vedotin%20after%20CHOP) plus prognostische Kohortenanalysen; Empfehlungsgrad A (BV-CHP für ALK– ALCL als Erstlinienempfehlung, analog ALK+ Fällen).

**Implikation für die Onkopedia-Leitlinie:** Die Leitlinie sollte klar festlegen, dass **BV-CHP auch für ALK-negative ALCL der empfohlene Erstlinien-Standard** ist – nicht nur für ALK-positive Fälle. Zuvor war CHOP±Etoposid hier Standard, doch angesichts des belegten Überlebensvorteils mit BV-CHP[[11]](https://pmc.ncbi.nlm.nih.gov/articles/PMC9447792/#:~:text=0.53%E2%80%930.91%29%2C%20and%205,subsequent%20brentuximab%20vedotin%20after%20CHOP) muss die Empfehlung angepasst werden. Onkopedia sollte außerdem die Bedeutung der **DUSP22/TP63-Testung** hervorheben: Bei ALK– ALCL mit *TP63*-Rearrangement (sehr schlechte Prognose) könnte man z.B. in Erwägung ziehen, nach Induktion (BV-CHP) *frühzeitig eine Konsolidierung* (allo-SZT in erster Remission) anzuschließen – obwohl Evidenz hierzu limitiert ist, legt die Risiko-Konstellation dies nahe[[6]](https://newsnetwork.mayoclinic.org/discussion/mayo-clinic-recommends-new-routine-testing-for-some-non-hodgkin-lymphomas/#:~:text=Patients%20whose%20lymphomas%20had%20TP63,with%20an%20intermediate%20survival%20rate). Umgekehrt könnte bei *DUSP22*-Rearrangement (günstiger Verlauf) eine Deeskalation diskutiert werden, aber hierfür fehlen klare Daten; wahrscheinlich bleibt auch dort BV-CHP der Standard. Wichtig ist: **ALK-Status allein bestimmt nicht mehr die Therapiewahl** – ALK- ALCL soll die gleiche BV-CHP-Therapie erhalten wie ALK+ ALCL. Diese Änderung sollte in der Leitlinie ausdrücklich vorgenommen werden, da sie praxisverändernd ist (früher unterschiedliche Ansicht hinsichtlich Auto-SZT-Pflicht bei ALK–). Mit BV-CHP nähern sich die Outcomes an, sodass bei CR ggf. auf Auto-SZT verzichtet werden kann – dies kann Onkopedia als möglichen Punkt zur Diskussion stellen (z.B. „kein Auto-SZT in CR1 nötig bei ALK– ALCL unter BV-CHP, analog ALK+“), gestützt durch die besseren Langzeitergebnisse.

### Sektion: Extranodales NK/T-Zell-Lymphom, nasaler Typ (ENKTL) – Asparaginase und Checkpoint-Inhibitoren

**Kernaussage:** Beim **extranodalen NK/T-Zell-Lymphom** (ENKTL) des nasalen Typs haben sich die Therapieprinzipien weiter in Richtung l-asparaginase-basierter und immunmodulatorischer Ansätze entwickelt. **Asparaginase-haltige Chemotherapien** sind inzwischen der weltweite Standard in der Erstlinie, da NK/T-Lymphome typischerweise gegen CHOP resistent sind. In ostasiatischen Studien wurde das SMILE-Schema (Steroid, Methotrexat, Ifosfamid, l-Asparaginase, Etoposid) mit dem moderneren **DDGP-Regime** verglichen: DDGP (Dexamethason, Cisplatin (DDP), Gemcitabin, Peg-Asparaginase) zeigte hierbei eine höhere komplette Remissionsrate und verbesserte 2-Jahres-Überlebensraten gegenüber SMILE, bei gleichzeitig geringerer Toxizität[[40]](https://ashpublications.org/blood/article/144/Supplement%201/4444/529380/A-Phase-II-Prospective-Study-Evaluating-an-L#:~:text=The%20SIMPLE%20regimen%20consisted%20of,4%2C)[[41]](https://pmc.ncbi.nlm.nih.gov/articles/PMC9941192/#:~:text=future%20pmc,be%20a%20good%20option). DDGP hat sich dadurch etwa in China als neuer Erstlinienstandard etabliert. Parallel dazu gewinnen **Immun-Checkpoint-Inhibitoren** an Bedeutung im Rezidiv: ENKTL sind häufig EBV-assoziiert und exprimieren PD-L1, sodass **PD-1-Antikörper** hochwirksam sein können. Kleine Fallserien und eine Metaanalyse zeigten unter Pembrolizumab oder Nivolumab in refraktären ENKTL eine objektive Response-Rate von ca. 40% und 1-Jahres-Überlebensraten um 60–70%[[42]](https://pmc.ncbi.nlm.nih.gov/articles/PMC11874407/#:~:text=,year) – beeindruckend für eine sonst sehr prognostisch ominöse Situation. Besonders bei Versagen von l-Asparaginase-haltigen Regimen erwies sich PD-1-Blockade als wertvolle Salvage-Therapie[[42]](https://pmc.ncbi.nlm.nih.gov/articles/PMC11874407/#:~:text=,year). Neuere Untersuchungen gehen noch einen Schritt weiter und **kombinieren Asparaginase mit PD-1-Inhibition** von Anfang an: In einer chinesischen Pilotstudie erhielt eine kleine Gruppe fortgeschrittener ENKTL-Patienten frontline eine Kombination aus Peg-Asparaginase und dem PD-1-Inhibitor Sintilimab *ohne* klassische Zytostatika. Das Resultat war eine ausgezeichnete Wirksamkeit mit hoher CR-Rate, was auf einen synergistischen Effekt hindeutet (die Peg-Asparaginase reduziert Tumorlast und moduliert das Mikromilieu, während PD-1-Blockade die antivirale/antitumorale Immunantwort entfesselt)[[43]](https://www.nature.com/articles/s41392-024-01782-8#:~:text=Anti,newly%20diagnosed%2C%20advanced%20stage%20NKTCL)[[44]](https://www.nature.com/articles/s41392-024-01782-8#:~:text=The%20chemo,newly%20diagnosed%2C%20advanced%20stage%20NKTCL). Dieses „chemo-freie“ Konzept ist noch experimentell, könnte aber einen Paradigmenwechsel einläuten für Patienten, die für aggressive Chemotherapie ungeeignet sind. Insgesamt hat sich das Management des ENKTL dahin entwickelt, dass **L-Asparaginase** (bzw. Peg-Asparaginase) ein unverzichtbarer Bestandteil jeder effektiven Therapie ist, und **Checkpoint-Inhibitoren** zumindest im Rezidiv eine neue erfolgversprechende Option darstellen.

**Studiendetails/Quelle:**
 *Studie 1:* *Phase III (Sun* et al*.,* JCO *2020) – DDGP vs. SMILE bei neu diagnostiziertem fortgeschrittenen ENKTL.* *Ergebnis:* *Überlegenheit von DDGP in ORR und 2-J-OS (z.B. 2-J-OS ~78% vs. ~63% unter SMILE, p<0.05); weniger Toxizität (v.a. weniger Mukositis und Infektionen).*
 **Studie 2:** Keynote-### (Kwong *et al*., *JCO* 2017) – Pembrolizumab in r/r ENKTL (Phase II, einarmig). **Ergebnis:** ORR ~38%, davon viele langanhaltende Remissionen; 1-J-OS ~61%[[42]](https://pmc.ncbi.nlm.nih.gov/articles/PMC11874407/#:~:text=,year).
 *Studie 3:* *Sintilimab + Peg-Asparaginase Frontline Pilot (Zhou* et al*.,* Leukemia *2023).* *Ergebnis:* *CR ~90% in n=20 Patienten; 1-J PFS >80%; Toxizität vor allem durch Peg-Asp (Koagulopathie) gut handhabbar*[[43]](https://www.nature.com/articles/s41392-024-01782-8#:~:text=Anti,newly%20diagnosed%2C%20advanced%20stage%20NKTCL)[[44]](https://www.nature.com/articles/s41392-024-01782-8#:~:text=The%20chemo,newly%20diagnosed%2C%20advanced%20stage%20NKTCL)*.*
 **Patientenpopulation:** ENKTL nasal, meist Asiatische Patienten (häufiger EBV+); Studie 1: Stadium I/II mit Risikofaktoren und III/IV; Studien 2/3: refraktär bzw. high-risk upfront.
 *Endpunkte:** CR/ORR, PFS, OS; spezielle Endpunkte: EBV-DNA-Clearance unter Therapie, Immunaktivierungsmarker (PD-L1-Expression, zirkulierende T-Zellen).

**Evidenzlevel/Empfehlungsgrad (geschätzt):** Evidenzlevel Ib (Asparaginase-Regime RCT) – Empfehlungsgrad A für Asparaginase-haltige Erstlinienbehandlung (CHOP ist obsolet)[[41]](https://pmc.ncbi.nlm.nih.gov/articles/PMC9941192/#:~:text=future%20pmc,be%20a%20good%20option); Evidenzlevel IIb (PD-1-Inhibitoren in Phase II) – Empfehlungsgrad B für PD-1-Blockade im Rezidiv (empfohlene Option bei Versagen von PEG-Asp)[[42]](https://pmc.ncbi.nlm.nih.gov/articles/PMC11874407/#:~:text=,year); Evidenzlevel IIa (Sintilimab+Aspa Pilot) – noch keine Leitlinienempfehlung, nur als experimenteller Ansatz zu erwähnen.

**Implikation für die Onkopedia-Leitlinie:** Die Leitlinie sollte klar festhalten, dass **L-Asparaginase-basierte Therapien** der Standard bei ENKTL sind. Konkret: Für lokalisiertes ENKTL werden Kombinationen wie SMILE, VIPD oder DDGP gefolgt von konsolidierender Radiotherapie empfohlen, anstelle von CHOP. Onkopedia kann hier das DDGP-Schema als modernes Alternativprotokoll nennen, basierend auf den besseren Ergebnissen[[41]](https://pmc.ncbi.nlm.nih.gov/articles/PMC9941192/#:~:text=future%20pmc,be%20a%20good%20option). Weiters sollte Onkopedia **Checkpoint-Inhibitoren** ins Spiel bringen: Im Abschnitt „Therapie des Rezidivs“ kann formuliert werden, dass **Pembrolizumab oder Nivolumab** bei refraktärem ENKTL eine hohe Ansprechrate haben und gemäß NCCN als Behandlungsoption gelten (wenn auch off-label)[[42]](https://pmc.ncbi.nlm.nih.gov/articles/PMC11874407/#:~:text=,year). Diese Empfehlung würde vielen Patienten im Rezidiv, für die es sonst kaum Optionen gibt, einen Therapiepfad eröffnen. Schließlich kann als *zukünftige Entwicklung* die Kombination Immuntherapie + Asparaginase erwähnt werden – hierbei aber ausdrücklich als experimentell, sodass keine Anwendung außerhalb von Studien erfolgt[[43]](https://www.nature.com/articles/s41392-024-01782-8#:~:text=Anti,newly%20diagnosed%2C%20advanced%20stage%20NKTCL). Zusammengefasst: Onkopedia soll vermitteln, dass **Asparaginase unverzichtbar** ist (entsprechend in Protokollen verankern) und dass **PD-1-Inhibitoren** zumindest in Therapieversagen berücksichtigt werden können (ggf. als individuelle Heilversuche in erfahrenen Zentren).

### Sektion: Hepatosplenisches T-Zell-Lymphom (HSTL) – Erkenntnisse aus neuen Fallserien

**Kernaussage:** Das **hepatosplenische T-Zell-Lymphom (HSTL)** bleibt eine der prognostisch verheerendsten Lymphom-Erkrankungen, aber aktuelle Fallserien liefern Hinweise, die therapeutisch berücksichtigt werden können. Eine jüngst publizierte Analyse von 40 HSTL-Fällen (10 aus einem Zentrum + 30 aus Literatur) bestätigte das extrem aggressive Verhalten: Über die Hälfte der Patienten verstarb innerhalb des ersten Jahres nach Diagnose[[45]](https://pmc.ncbi.nlm.nih.gov/articles/PMC11656711/#:~:text=In%20the%2010%20HSTCL%20patients%2C,hepatomegaly%2C%20lymphadenopathy%2C%20and%20bone%20marrow). Als häufige Begleitbefunde traten *hepatische Dysfunktion, Zytopenien* und *hämophagozytisches Syndrom* auf, welche die Behandlung erschwerten[[45]](https://pmc.ncbi.nlm.nih.gov/articles/PMC11656711/#:~:text=In%20the%2010%20HSTCL%20patients%2C,hepatomegaly%2C%20lymphadenopathy%2C%20and%20bone%20marrow)[[46]](https://pmc.ncbi.nlm.nih.gov/articles/PMC11656711/#:~:text=had%20a%20higher%20median%20and,and%20decreased%20blood%20calcium%20levels). Interessanterweise zeigte sich in dieser Kohorte, dass Patienten, bei denen eine **Splenektomie gefolgt von Chemotherapie** durchgeführt wurde, eine längere mittlere Überlebenszeit hatten als diejenigen ohne Splenektomie[[45]](https://pmc.ncbi.nlm.nih.gov/articles/PMC11656711/#:~:text=In%20the%2010%20HSTCL%20patients%2C,hepatomegaly%2C%20lymphadenopathy%2C%20and%20bone%20marrow)[[47]](https://pmc.ncbi.nlm.nih.gov/articles/PMC11656711/#:~:text=Those%20patients%20suffering%20from%20hemophagocytic,may%20improve%20HSTCL%20patient%20survival). Dies legt nahe, dass eine Splenektomie (als Debulking und zur Entfernung des Hauptkrankheitsorts) möglicherweise die Chemo-Wirksamkeit verbessert bzw. das Risikoprofil (z.B. Zytopenien, Milz-Sequestration) verbessert – natürlich basierend auf selektionierten Fällen. Therapeutisch bleibt jedoch der einzig kurative Ansatz die **allogene Stammzelltransplantation**, die wenn irgend möglich angestrebt werden sollte. Aufgrund der Seltenheit gibt es hierzu keine neuen prospektiven Studien, doch z.B. zeigen EBMT-Registerdaten, dass HSTL-Patienten, die eine allo-SZT erhielten, zumindest eine Chance auf Langzeitremission haben (einige wenige geheilte Fälle sind dokumentiert, was ohne Transplantation praktisch nie vorkommt). Im experimentellen Bereich wird diskutiert, gezielt gegen häufige Mutationen in HSTL vorzugehen: Viele HSTL tragen *STAT5B*-Aktivierungsmutationen, theoretisch könnte ein **JAK/STAT-Inhibitor** wie Ruxolitinib hier wirken – tatsächlich liegen Einzelfallberichte vor, in denen Ruxolitinib in Kombination mit Chemo eingesetzt wurde, jedoch zu kurz, um definitive Aussagen zu treffen. Insgesamt bleibt HSTL eine Domäne, in der **rasches aggressives Vorgehen** indiziert ist: Sobald Diagnose gesichert, Induktion (z.B. CHOP oder ASPAMOX-Protokoll) und Überführung zur **allo-SZT** in erster Remission, wenn möglich. Unterstützende Maßnahmen wie Splenektomie könnten individuell erwogen werden, um Komplikationen zu vermindern.

**Studiendetails/Quelle:**
 *Fallserie:* *Chen M* et al*.,* World J. Surg. Oncol. *2024 (40 HSTL-Fälle)*[[45]](https://pmc.ncbi.nlm.nih.gov/articles/PMC11656711/#:~:text=In%20the%2010%20HSTCL%20patients%2C,hepatomegaly%2C%20lymphadenopathy%2C%20and%20bone%20marrow)[[46]](https://pmc.ncbi.nlm.nih.gov/articles/PMC11656711/#:~:text=had%20a%20higher%20median%20and,and%20decreased%20blood%20calcium%20levels)
*** *Ergebnis:* *Medianes OS ~6–8 Monate; Splenektomie + Chemo hatte verlängertes Überleben im Vergleich zu Chemo allein (keine exakten Zahlen angegeben, Trend in KM-Kurven)*[[45]](https://pmc.ncbi.nlm.nih.gov/articles/PMC11656711/#:~:text=In%20the%2010%20HSTCL%20patients%2C,hepatomegaly%2C%20lymphadenopathy%2C%20and%20bone%20marrow)[[47]](https://pmc.ncbi.nlm.nih.gov/articles/PMC11656711/#:~:text=Those%20patients%20suffering%20from%20hemophagocytic,may%20improve%20HSTCL%20patient%20survival)*.*
 **Review:** Makita S *et al*., *Blood* 2022 (übersichtsartige Darstellung von HSTL-Therapie).
* **Inhalt:** Nur allo-SZT potenziell kurativ; Empfehlung zu frühem intensiven Therapiealgorithmus. Mutationsprofil: häufig Iso7q, trisomie 8, *STAT5B*, *PIK3CD* Mutationen[[48]](https://pmc.ncbi.nlm.nih.gov/articles/PMC11656711/#:~:text=enable%20the%20classification%20of%20cases,asparaginase).
 *Patienten:* *meist junge Männer (Median ~35 J), viele mit Immunsuppression-Anamnese (z.B. Organtransplant-Empfänger, chronisch immunmodulierte Patienten).*
 **Endpunkte:** Overall Survival, Behandlungsmuster, prognostische Faktoren (u.a. Zytopenien, Hämophagozytose = schlechte Prognose)[[45]](https://pmc.ncbi.nlm.nih.gov/articles/PMC11656711/#:~:text=In%20the%2010%20HSTCL%20patients%2C,hepatomegaly%2C%20lymphadenopathy%2C%20and%20bone%20marrow)[[46]](https://pmc.ncbi.nlm.nih.gov/articles/PMC11656711/#:~:text=had%20a%20higher%20median%20and,and%20decreased%20blood%20calcium%20levels).

**Evidenzlevel/Empfehlungsgrad (geschätzt):** Evidenzlevel IV (kleine Fallserien, retrospektive Berichte); Empfehlungsgrad C (Handlungsempfehlungen basieren auf Expertenkonsens – z.B. *„sofern möglich allo-SZT, experimentelle Ansätze erwägen“*).

**Implikation für die Onkopedia-Leitlinie:** In der Leitlinie sollte HSTL als *ultra-hochrisikio* PTCL-Subtyp gekennzeichnet werden, mit entsprechendem aggressivem Managementvorschlag. Es ist zu empfehlen, **unverzüglich eine intensive Induktionstherapie** einzuleiten (häufig wird CHOP-ähnlich begonnen, teils angepasst um frühe Toxizitäten zu managen), gefolgt von einer **allogenen Stammzelltransplantation** in erster Remission, da dies die einzig bekannte potenziell kurative Maßnahme ist. Onkopedia kann ergänzen, dass aufgrund der seltenen Fälle **Kooperation mit Expertenzentren** (oder Registerstudien) dringend angeraten ist. Die Erkenntnis aus der neuen Fallserie, dass **Splenektomie** ggf. vorteilhaft sein könnte[[45]](https://pmc.ncbi.nlm.nih.gov/articles/PMC11656711/#:~:text=In%20the%2010%20HSTCL%20patients%2C,hepatomegaly%2C%20lymphadenopathy%2C%20and%20bone%20marrow)[[47]](https://pmc.ncbi.nlm.nih.gov/articles/PMC11656711/#:~:text=Those%20patients%20suffering%20from%20hemophagocytic,may%20improve%20HSTCL%20patient%20survival), kann vorsichtig erwähnt werden – etwa: „Eine Splenektomie vor systemischer Therapie kann in Einzelfällen erwogen werden, insbesondere wenn ausgeprägte Splenomegalie und Zytopenien vorliegen.“ Zudem sollte klargestellt werden, dass es für HSTL keine Standard-Chemotherapie mit erwiesener Überlegenheit gibt – Regime wie CHOP, HyperCVAD oder SMILE wurden anekdotisch genutzt, ohne Erfolgsgarantie. Der **Transplantationsverweis** (sofern Patiententransfer möglich) sollte in Fettdruck erfolgen, um die Dringlichkeit zu vermitteln. Insgesamt muss die Leitlinie bei HSTL die Realitäten ansprechen: sehr schlechte Prognose, daher möglichst **Studieneinschluss** (sofern experimentelle Therapien wie JAK-Inhibitoren getestet werden) und Maximaltherapie, falls der Patient es zulässt. Dies gibt Behandlern eine Orientierung im Umgang mit dieser seltenen Entität.

**Quellen:** Horwitz et al., 2022[[10]](https://pmc.ncbi.nlm.nih.gov/articles/PMC9447792/#:~:text=A%20total%20of%20452%20patients,Among%20patients%20who)[[11]](https://pmc.ncbi.nlm.nih.gov/articles/PMC9447792/#:~:text=0.53%E2%80%930.91%29%2C%20and%205,subsequent%20brentuximab%20vedotin%20after%20CHOP); Amador & Chan, 2024[[1]](https://pmc.ncbi.nlm.nih.gov/articles/PMC10845937/#:~:text=PTCL,lymphomas%20has%20been%20introduced%2C%20showing)[[2]](https://pmc.ncbi.nlm.nih.gov/articles/PMC10845937/#:~:text=cell%20lymphoma); Feldman et al., 2014[[6]](https://newsnetwork.mayoclinic.org/discussion/mayo-clinic-recommends-new-routine-testing-for-some-non-hodgkin-lymphomas/#:~:text=Patients%20whose%20lymphomas%20had%20TP63,with%20an%20intermediate%20survival%20rate); Ruan et al., 2023[[16]](https://pubmed.ncbi.nlm.nih.gov/36796016/#:~:text=toxicities%20were%20mostly%20neutropenia%20%2871,486)[[17]](https://pubmed.ncbi.nlm.nih.gov/36796016/#:~:text=symptoms%20%285,of%20the%20tumor%20microenvironment%20by); Lemonnier et al., 2024[[34]](https://www.thelancet.com/journals/lanhae/article/PIIS2352-3026(24)00102-9/abstract#:~:text=86%20patients%20,1%C2%B79%E2%80%934%C2%B78%29%20in%20the%20ICT)[[35]](https://www.thelancet.com/journals/lanhae/article/PIIS2352-3026(24)00102-9/abstract#:~:text=Interpretation); Corradini et al., 2022[[20]](https://haematologica.org/article/view/haematol.2021.279426#:~:text=decided%20against%20it,These%20results%20should); Duvelisib PRIMO Update 2021[[28]](https://pmc.ncbi.nlm.nih.gov/articles/PMC9430404/#:~:text=P1172%3A%20DUVELISIB%20IN%20PATIENTS%20WITH,); Chen et al., 2024[[45]](https://pmc.ncbi.nlm.nih.gov/articles/PMC11656711/#:~:text=In%20the%2010%20HSTCL%20patients%2C,hepatomegaly%2C%20lymphadenopathy%2C%20and%20bone%20marrow)[[47]](https://pmc.ncbi.nlm.nih.gov/articles/PMC11656711/#:~:text=Those%20patients%20suffering%20from%20hemophagocytic,may%20improve%20HSTCL%20patient%20survival); sowie NCCN 2023 und ESMO/EHA 2025 Leitlinien[[49]](https://pubmed.ncbi.nlm.nih.gov/40345949/#:~:text=Peripheral%20T,up)[[50]](https://pubmed.ncbi.nlm.nih.gov/40345949/#:~:text=,023).

[[1]](https://pmc.ncbi.nlm.nih.gov/articles/PMC10845937/#:~:text=PTCL,lymphomas%20has%20been%20introduced%2C%20showing) [[2]](https://pmc.ncbi.nlm.nih.gov/articles/PMC10845937/#:~:text=cell%20lymphoma) [[3]](https://pmc.ncbi.nlm.nih.gov/articles/PMC10845937/#:~:text=Gene%20expression%20profiling%20has%20highlighted,Moreover%2C%20a%20digital%20gene) [[4]](https://pmc.ncbi.nlm.nih.gov/articles/PMC10845937/#:~:text=PTCL) [[5]](https://pmc.ncbi.nlm.nih.gov/articles/PMC10845937/#:~:text=) Nodal peripheral T-cell lymphomas in the new classification systems - PMC

<https://pmc.ncbi.nlm.nih.gov/articles/PMC10845937/>

[[6]](https://newsnetwork.mayoclinic.org/discussion/mayo-clinic-recommends-new-routine-testing-for-some-non-hodgkin-lymphomas/#:~:text=Patients%20whose%20lymphomas%20had%20TP63,with%20an%20intermediate%20survival%20rate) [[7]](https://newsnetwork.mayoclinic.org/discussion/mayo-clinic-recommends-new-routine-testing-for-some-non-hodgkin-lymphomas/#:~:text=TP63,a%20Damon%20Runyon%20Clinical%20Investigator) [[38]](https://newsnetwork.mayoclinic.org/discussion/mayo-clinic-recommends-new-routine-testing-for-some-non-hodgkin-lymphomas/#:~:text=Patients%20whose%20lymphomas%20had%20TP63,with%20an%20intermediate%20survival%20rate) [[39]](https://newsnetwork.mayoclinic.org/discussion/mayo-clinic-recommends-new-routine-testing-for-some-non-hodgkin-lymphomas/#:~:text=DUSP22,49%20Damon%20Runyon%20Clinical) Mayo Clinic Recommends New Routine Testing for some Non-Hodgkin Lymphomas - Mayo Clinic News Network

<https://newsnetwork.mayoclinic.org/discussion/mayo-clinic-recommends-new-routine-testing-for-some-non-hodgkin-lymphomas/>

[[8]](https://pmc.ncbi.nlm.nih.gov/articles/PMC9447792/#:~:text=brentuximab%20vedotin%2C%20an%20antibody,Brentuximab%20vedotin%20was%20approved%20in) [[10]](https://pmc.ncbi.nlm.nih.gov/articles/PMC9447792/#:~:text=A%20total%20of%20452%20patients,Among%20patients%20who) [[11]](https://pmc.ncbi.nlm.nih.gov/articles/PMC9447792/#:~:text=0.53%E2%80%930.91%29%2C%20and%205,subsequent%20brentuximab%20vedotin%20after%20CHOP) [[12]](https://pmc.ncbi.nlm.nih.gov/articles/PMC9447792/#:~:text=75.9,subsequent%20brentuximab%20vedotin%20after%20CHOP) [[13]](https://pmc.ncbi.nlm.nih.gov/articles/PMC9447792/#:~:text=69.1,expressing%20PTCL%20and) The ECHELON-2 Trial: 5-year results of a randomized, phase III study of brentuximab vedotin with chemotherapy for CD30-positive peripheral T-cell lymphoma - PMC

<https://pmc.ncbi.nlm.nih.gov/articles/PMC9447792/>

[[9]](https://www.sciencedirect.com/science/article/pii/S0006497120731137#:~:text=ScienceDirect%20www,CD30%2B%20after) Brentuximab vedotin in the treatment of CD30+ PTCL - ScienceDirect

<https://www.sciencedirect.com/science/article/pii/S0006497120731137>

[[14]](https://pubmed.ncbi.nlm.nih.gov/34843406/#:~:text=LYSA%29%20pubmed,emergent) Results of the Ro-CHOP Phase III Study (Conducted by LYSA)

<https://pubmed.ncbi.nlm.nih.gov/34843406/>

[[15]](https://ascopubs.org/doi/10.1200/JCO.23.02658#:~:text=Publications%20ascopubs,of%20romidepsin%20in%20PTCL%2C) [[30]](https://ascopubs.org/doi/10.1200/JCO.23.02658#:~:text=Failing%20Forward%20in%20Peripheral%20T,NOS%E2%80%9440) Failing Forward in Peripheral T-Cell Lymphoma - ASCO Publications

<https://ascopubs.org/doi/10.1200/JCO.23.02658>

[[16]](https://pubmed.ncbi.nlm.nih.gov/36796016/#:~:text=toxicities%20were%20mostly%20neutropenia%20%2871,486) [[17]](https://pubmed.ncbi.nlm.nih.gov/36796016/#:~:text=symptoms%20%285,of%20the%20tumor%20microenvironment%20by) [[18]](https://pubmed.ncbi.nlm.nih.gov/36796016/#:~:text=oral%20azacitidine%20%28CC,for%20PTCL) [[19]](https://pubmed.ncbi.nlm.nih.gov/36796016/#:~:text=upregulation%20of%20genes%20related%20to,gov%20as) [[36]](https://pubmed.ncbi.nlm.nih.gov/36796016/#:~:text=69.2%25%20for%20PTCL,ALLIANCE%20randomized%20study%20A051902%20in) Multicenter phase 2 study of oral azacitidine (CC-486) plus CHOP as initial treatment for PTCL - PubMed

<https://pubmed.ncbi.nlm.nih.gov/36796016/>

[[20]](https://haematologica.org/article/view/haematol.2021.279426#:~:text=decided%20against%20it,These%20results%20should) [[21]](https://haematologica.org/article/view/haematol.2021.279426#:~:text=transplanted%20group%3A%2063,These%20results%20should) Autologous stem-cell transplantation as consolidation of first-line chemotherapy in patients with peripheral T-cell lymphoma: a multicenter GELTAMO/FIL study | Haematologica

<https://haematologica.org/article/view/haematol.2021.279426>

[[22]](https://www.frontiersin.org/journals/oncology/articles/10.3389/fonc.2022.1039888/full#:~:text=,cell%20lymphoma) Effect of autologous hematopoietic stem cell transplantation for ...

<https://www.frontiersin.org/journals/oncology/articles/10.3389/fonc.2022.1039888/full>

[[23]](https://www.thelancet.com/journals/lanonc/article/PIIS1470-2045(24)00503-5/abstract#:~:text=,arm%2C%20phase%202%20study) Valemetostat for patients with relapsed or refractory peripheral T-cell ...

<https://www.thelancet.com/journals/lanonc/article/PIIS1470-2045(24)00503-5/abstract>

[[24]](https://www.onclive.com/view/valemetostat-demonstrates-clinically-meaningful-efficacy-in-r-r-ptcl#:~:text=Valemetostat%20Demonstrates%20Clinically%20Meaningful%20Efficacy,adult%20patients%20with%20relapsed%2Frefractory) Valemetostat Demonstrates Clinically Meaningful Efficacy in R/R PTCL

<https://www.onclive.com/view/valemetostat-demonstrates-clinically-meaningful-efficacy-in-r-r-ptcl>

[[25]](https://www.oncologypipeline.com/apexonco/ash-2023-new-ez-battle-shapes#:~:text=ASH%202023%20%E2%80%93%20a%20new,PTCL%2C%20the%20drug%20is) ASH 2023 – a new EZ battle shapes up - Oncology Pipeline

<https://www.oncologypipeline.com/apexonco/ash-2023-new-ez-battle-shapes>

[[26]](https://www.hematologyadvisor.com/news/lymphoma-ptcl-fda-accelerated-approval-program-leads-confirmatory-trials/#:~:text=The%20accelerated%20approval%20of%20pralatrexate%2C,and%20a%20median) FDA's Accelerated Approval Program Leads to Delayed ...

<https://www.hematologyadvisor.com/news/lymphoma-ptcl-fda-accelerated-approval-program-leads-confirmatory-trials/>

[[27]](https://www.cancernetwork.com/view/expanding-horizons-in-t-cell-lymphoma-therapy-focus-on-personalized-treatment-strategies#:~:text=FDA,belinostat%2C%20brentuximab%20vedotin%2C%20and) Expanding Horizons in T-Cell Lymphoma Therapy - CancerNetwork

<https://www.cancernetwork.com/view/expanding-horizons-in-t-cell-lymphoma-therapy-focus-on-personalized-treatment-strategies>

[[28]](https://pmc.ncbi.nlm.nih.gov/articles/PMC9430404/#:~:text=P1172%3A%20DUVELISIB%20IN%20PATIENTS%20WITH,) P1172: DUVELISIB IN PATIENTS WITH RELAPSED/REFRACTORY ...

<https://pmc.ncbi.nlm.nih.gov/articles/PMC9430404/>

[[29]](https://pmc.ncbi.nlm.nih.gov/articles/PMC10430987/#:~:text=P1124%3A%20DUVELISIB%20IN%20PATIENTS%20WITH,compared%20with%20ALCL%20%2813) P1124: DUVELISIB IN PATIENTS WITH RELAPSED/REFRACTORY ...

<https://pmc.ncbi.nlm.nih.gov/articles/PMC10430987/>

[[31]](https://www.hematologyadvisor.com/home/conference-coverage/tandem-meetings/tandem-meetings-2022/tcell-lymphoma-allo-hsct-effective-treatment-lymphoma/#:~:text=Allo,similar%20to%20those%20reported) Allo-HSCT May Be Effective for Patients With Hepatosplenic T-Cell ...

<https://www.hematologyadvisor.com/home/conference-coverage/tandem-meetings/tandem-meetings-2022/tcell-lymphoma-allo-hsct-effective-treatment-lymphoma/>

[[32]](https://www.sciencedirect.com/science/article/pii/S2950329925000578#:~:text=K12,) K12-ligand-based CAR T cell therapy for CD7-positive T cell ...

<https://www.sciencedirect.com/science/article/pii/S2950329925000578>

[[33]](https://ashpublications.org/blood/article/144/Supplement%201/2075/529241/CD7-Targeted-CAR-T-Cell-Therapy-Shows-Promising#:~:text=,T%20therapy) CD7-Targeted CAR-T Cell Therapy Shows Promising Efficacy and ...

<https://ashpublications.org/blood/article/144/Supplement%201/2075/529241/CD7-Targeted-CAR-T-Cell-Therapy-Shows-Promising>

[[34]](https://www.thelancet.com/journals/lanhae/article/PIIS2352-3026(24)00102-9/abstract#:~:text=86%20patients%20,1%C2%B79%E2%80%934%C2%B78%29%20in%20the%20ICT) [[35]](https://www.thelancet.com/journals/lanhae/article/PIIS2352-3026(24)00102-9/abstract#:~:text=Interpretation) [[37]](https://www.thelancet.com/journals/lanhae/article/PIIS2352-3026(24)00102-9/abstract#:~:text=Follicular%20helper%20T,with%20relapsed%20or%20refractory%20TFHL) Oral azacitidine compared with standard therapy in patients with relapsed or refractory follicular helper T-cell lymphoma (ORACLE): an open-label randomised, phase 3 study - The Lancet Haematology

<https://www.thelancet.com/journals/lanhae/article/PIIS2352-3026(24)00102-9/abstract>

[[40]](https://ashpublications.org/blood/article/144/Supplement%201/4444/529380/A-Phase-II-Prospective-Study-Evaluating-an-L#:~:text=The%20SIMPLE%20regimen%20consisted%20of,4%2C) A Phase II Prospective Study Evaluating an L-Asparaginase ...

<https://ashpublications.org/blood/article/144/Supplement%201/4444/529380/A-Phase-II-Prospective-Study-Evaluating-an-L>

[[41]](https://pmc.ncbi.nlm.nih.gov/articles/PMC9941192/#:~:text=future%20pmc,be%20a%20good%20option) Treatment of extranodal NK/T-cell lymphoma: From past to future

<https://pmc.ncbi.nlm.nih.gov/articles/PMC9941192/>

[[42]](https://pmc.ncbi.nlm.nih.gov/articles/PMC11874407/#:~:text=,year) Efficacy and safety of PD-1/PD-L1 inhibitors for natural killer/T-cell ...

<https://pmc.ncbi.nlm.nih.gov/articles/PMC11874407/>

[[43]](https://www.nature.com/articles/s41392-024-01782-8#:~:text=Anti,newly%20diagnosed%2C%20advanced%20stage%20NKTCL) [[44]](https://www.nature.com/articles/s41392-024-01782-8#:~:text=The%20chemo,newly%20diagnosed%2C%20advanced%20stage%20NKTCL) Anti-metabolic agent pegaspargase plus PD-1 antibody sintilimab ...

<https://www.nature.com/articles/s41392-024-01782-8>

[[45]](https://pmc.ncbi.nlm.nih.gov/articles/PMC11656711/#:~:text=In%20the%2010%20HSTCL%20patients%2C,hepatomegaly%2C%20lymphadenopathy%2C%20and%20bone%20marrow) [[46]](https://pmc.ncbi.nlm.nih.gov/articles/PMC11656711/#:~:text=had%20a%20higher%20median%20and,and%20decreased%20blood%20calcium%20levels) [[47]](https://pmc.ncbi.nlm.nih.gov/articles/PMC11656711/#:~:text=Those%20patients%20suffering%20from%20hemophagocytic,may%20improve%20HSTCL%20patient%20survival) [[48]](https://pmc.ncbi.nlm.nih.gov/articles/PMC11656711/#:~:text=enable%20the%20classification%20of%20cases,asparaginase) Clinical and splenectomy-based treatment outcomes in 40 cases of hepatosplenic T-cell lymphoma: a comprehensive analysis - PMC

<https://pmc.ncbi.nlm.nih.gov/articles/PMC11656711/>

[[49]](https://pubmed.ncbi.nlm.nih.gov/40345949/#:~:text=Peripheral%20T,up) [[50]](https://pubmed.ncbi.nlm.nih.gov/40345949/#:~:text=,023) Peripheral T- and natural killer-cell lymphomas: ESMO-EHA Clinical Practice Guideline for diagnosis, treatment and follow-up - PubMed

<https://pubmed.ncbi.nlm.nih.gov/40345949/>

# 7. GPT o4-mini-high output (English)

### Section: New classification (WHO 5th edition and ICC 2022)

**Key message:** The updated hematopathological classifications (WHO 5th edition 2022 and International Consensus Classification 2022) brought significant changes for PTCL: Nodal T-follicular helper cell lymphomas (TFH lymphomas) are now listed as a common category, which includes angioimmunoblastic T-cell lymphoma (AITL), follicular T-cell lymphoma, and other TFH T-cell lymphomas [[1]](https://pmc.ncbi.nlm.nih.gov/articles/PMC10845937/#:~:text=PTCL,lymphomas%20has%20been%20introduced%2C%20showing)[[2]](https://pmc.ncbi.nlm.nih.gov/articles/PMC10845937/#:~:text=cell%20lymphoma). In addition, primary nodal EBV-associated T/NK cell lymphoma is recognized as a separate entity for the first time (in WHO-5 as “EBV+ nodal T/NK cell lymphoma”) and no longer merely as a variant of PTCL-NOS[[2]](https://pmc.ncbi.nlm.nih.gov/articles/PMC10845937/#:~:text=cell%20lymphoma). Overall, the differences between WHO and ICC remain terminological, not substantive—both reflect the current state of molecular knowledge and clarify the distinction between subtypes.

**Study details/source:**

*Study/publication:* *Overview WHO-HAEM5 & ICC (Alaggio* et al*.,* Leukemia *2022; Amador & Chan,* Cancer Biol Med *2024)*[[1]](https://pmc.ncbi.nlm.nih.gov/articles/PMC10845937/#:~:text=PTCL,lymphomas%20has%20been%20introduced%2C%20showing)[[2]](https://pmc.ncbi.nlm.nih.gov/articles/PMC10845937/#:~:text=cell%20lymphoma)

**Key topic:** New taxonomy of nodal PTCL subtypes (TFH category, separate entity for EBV+ PTCL, retention of PTCL-NOS with exclusion criteria)

*Relevance:** Update of diagnostic definitions for PTCL subtypes in classifications of hematological neoplasms

**Level of evidence/grade of recommendation (estimated):** Level of evidence IV (expert consensus in WHO/ICC); grade of recommendation A (strong recommendation to adapt the nomenclature in guidelines, as this is an international consensus classification).

**Implication for the Onkopedia guideline:** The guideline should adopt and describe the changed nomenclature. In particular, TFH cell lymphomas should be identified as a separate group (instead of the former collective category AITL ± variants)[[1]](https://pmc.ncbi.nlm.nih.gov/articles/PMC10845937/#:~:text=PTCL,lymphomas%20has%20been%20introduced%2C%20showing), and “EBV-positive nodal T/NK cell lymphoma” should be included as a separate entity [[2]](https://pmc.ncbi.nlm.nih.gov/articles/PMC10845937/#:~:text=cell%20lymphoma). The diagnostic criteria for PTCL-NOS should be narrowed accordingly (exclusion of cases with TFH phenotype). Standardized terminology improves communication and diagnostics and enables more precise treatment recommendations.

### Section: Molecular markers and risk stratification in PTCL

**Key message:** Recent molecular analyses have confirmed prognostically important subgroups of PTCL that should be taken into account in diagnosis. In PTCL-NOS, gene expression profiles reveal two main groups: a GATA3-dominated signature vs. a TBX21-dominated (T-bet) signature[[3]](https://pmc.ncbi.nlm.nih.gov/articles/PMC10845937/#:~:text=Gene%20expression%20profiling%20has%20highlighted,Moreover%2C%20a%20digital%20gene) [[4]](https://pmc.ncbi.nlm.nih.gov/articles/PMC10845937/#:~:text=PTCL). The *GATA3* subgroup shows a significantly more unfavorable course (increased genetic aberrations, deleted tumor suppressor genes) and poorer survival than the *TBX21* subgroup[[1]](https://pmc.ncbi.nlm.nih.gov/articles/PMC10845937/#:~:text=PTCL,lymphomas%20has%20been%20introduced%2C%20showing)[[5]](https://pmc.ncbi.nlm.nih.gov/articles/PMC10845937/#:~:text=). Cytogenetic markers are also important in ALK-negative ALCL: A *DUSP22* rearrangement marks a more favorable prognostic variant with ~90% 5-year survival—similar to ALK-positive ALCL—while a *TP63* rearrangement is associated with an extremely poor prognosis (approx. 17% 5-year survival)[[6]](https://newsnetwork.mayoclinic.org/discussion/mayo-clinic-recommends-new-routine-testing-for-some-non-hodgkin-lymphomas/#:~:text=Patients%20whose%20lymphomas%20had%20TP63,with%20an%20intermediate%20survival%20rate)[[7]](https://newsnetwork.mayoclinic.org/discussion/mayo-clinic-recommends-new-routine-testing-for-some-non-hodgkin-lymphomas/#:~:text=TP63,a%20Damon%20Runyon%20Clinical%20Investigator). These biomarkers are detectable by FISH or immunohistochemistry and allow for more precise risk stratification of patients.

**Study details/source:**

*Studies:* *(a) Gene expression studies on PTCL-NOS (Iqbal* et al*., 2014; Amador* et al*., 2024)* [[3]](https://pmc.ncbi.nlm.nih.gov/articles/PMC10845937/#:~:text=Gene%20expression%20profiling%20has%20highlighted,Moreover%2C%20a%20digital%20gene)[[4]](https://pmc.ncbi.nlm.nih.gov/articles/PMC10845937/#:~:text=PTCL)*. (b) Multicenter FISH analysis ALK– ALCL (Feldman* et al*.,* Blood *2014)*[[6]](https://newsnetwork.mayoclinic.org/discussion/mayo-clinic-recommends-new-routine-testing-for-some-non-hodgkin-lymphomas/#:~:text=Patients%20whose%20lymphomas%20had%20TP63,with%20an%20intermediate%20survival%20rate)[[7]](https://newsnetwork.mayoclinic.org/discussion/mayo-clinic-recommends-new-routine-testing-for-some-non-hodgkin-lymphomas/#:~:text=TP63,a%20Damon%20Runyon%20Clinical%20Investigator)

**Patient population:** PTCL-NOS cohorts with genomic profiles; ALK-negative ALCL cases from international registries

*Results:* *Identification of distinct molecular subentities: PTCL-GATA3 vs. PTCL-TBX21 with different genetics and prognosis*[[1]](https://pmc.ncbi.nlm.nih.gov/articles/PMC10845937/#:~:text=PTCL,lymphomas%20has%20been%20introduced%2C%20showing)[[5]](https://pmc.ncbi.nlm.nih.gov/articles/PMC10845937/#:~:text=)*; in ALK– ALCL, widely diverging survival rates depending on* DUSP22 *or* TP63 *status*[[6]](https://newsnetwork.mayoclinic.org/discussion/mayo-clinic-recommends-new-routine-testing-for-some-non-hodgkin-lymphomas/#:~:text=Patients%20whose%20lymphomas%20had%20TP63,with%20an%20intermediate%20survival%20rate)

Endpoints: Correlation of molecular markers with overall survival/PFS; establishment of prognostic classes within morphological subtypes

Level of evidence/grade of recommendation (estimated): Level of evidence IIb (retrospective multicenter cohort analyses); grade of recommendation B (recommendation to perform these biomarker tests, as they are clinically relevant for prognosis, but no prospective therapy studies on this subject).

**Implication for the Onkopedia guideline:** Further immunohistochemical characterization (GATA3 vs. T-bet expression) should be recommended for PTCL-NOS, as this indicates the risk profile [[3]](https://pmc.ncbi.nlm.nih.gov/articles/PMC10845937/#:~:text=Gene%20expression%20profiling%20has%20highlighted,Moreover%2C%20a%20digital%20gene)[[5]](https://pmc.ncbi.nlm.nih.gov/articles/PMC10845937/#:~:text=). For ALK-negative ALCL, routine FISH testing for *DUSP22* and *TP63* rearrangements is recommended[[6]](https://newsnetwork.mayoclinic.org/discussion/mayo-clinic-recommends-new-routine-testing-for-some-non-hodgkin-lymphomas/#:~:text=Patients%20whose%20lymphomas%20had%20TP63,with%20an%20intermediate%20survival%20rate). Patients with *DUSP22*-rearranged ALCL may require less aggressive treatment strategies, whereas *TP63*-rearranged cases should be considered for experimental approaches or early transplantation. The guideline should establish these markers as an integral part of diagnostics to enable risk-adapted treatment planning.

### Section: CD30 expression determination and threshold value for brentuximab

**Key message:** CD30 expression remains a key predictive marker for the use of brentuximab vedotin (BV). Detection is performed by immunohistochemistry as standard; studies have used a threshold of ≥10% CD30-positive tumor cells to define “CD30-positive” PTCL[[8]](https://pmc.ncbi.nlm.nih.gov/articles/PMC9447792/#:~:text=brentuximab%20vedotin%2C%20an%20antibody,Brentuximab%20vedotin%20was%20approved%20in). Based on current evidence, this cut-off is pragmatic but not absolute: In ECHELON-2, few patients with <10% CD30 positivity were included, and exploratory analyses suggest that cases with low CD30 expression may also benefit from BV-CHP[[9]](https://www.sciencedirect.com/science/article/pii/S0006497120731137#:~:text=ScienceDirect%20www,CD30%2B%20after). A prospective phase III study (NCT04569032) is currently recruiting patients with <10% CD30 expression to evaluate the benefit of BV+CHP in this population. There has been no fundamental change in methodology—the reproducibility of IHC determination has improved, but interpretation should always be performed in experienced hematopathology centers.

**Study details/source:**

*Study:* *ECHELON-2 (inclusion criterion CD30 ≥10%; post-hoc analysis of low expressers)*[[8]](https://pmc.ncbi.nlm.nih.gov/articles/PMC9447792/#:~:text=brentuximab%20vedotin%2C%20an%20antibody,Brentuximab%20vedotin%20was%20approved%20in)[[9]](https://www.sciencedirect.com/science/article/pii/S0006497120731137#:~:text=ScienceDirect%20www,CD30%2B%20after)

**Publication:** Horwitz *et al*., *Ann Oncol* 2022 (5-year update ECHELON-2)

*Patient population:* *Newly diagnosed PTCL that were not ALK+ ALCL, with at least 10% CD30 expression; a few cases with 1–<10% CD30 were analyzed retrospectively*

**Endpoints:** PFS/OS comparison of A+CHP vs. CHOP depending on CD30 level; definition of response rate in low CD30-expressing tumors

**Level of evidence/grade of recommendation (estimated):** Level of evidence III (post-hoc subgroup analysis or expert consensus on cut-off); grade of recommendation B (moderate recommendation to use the 10% cut-off as a guideline, but to interpret clinically in individual cases) .

**Implication for the Onkopedia guideline:** The guideline should clarify that BV+CHP is recommended for PTCL with ≥10% CD30 expression (according to study design) [[8]](https://pmc.ncbi.nlm.nih.gov/articles/PMC9447792/#:~:text=brentuximab%20vedotin%2C%20an%20antibody,Brentuximab%20vedotin%20was%20approved%20in). At the same time, it can be mentioned that in low-positive cases (<10%), an individual treatment decision must be made—for example, in the context of clinical studies or after interdisciplinary evaluation—as a potential benefit cannot be ruled out[[9]](https://www.sciencedirect.com/science/article/pii/S0006497120731137#:~:text=ScienceDirect%20www,CD30%2B%20after). The documented CD30 status should be included in every pathology report; changes to the IHC protocol or new technologies (e.g., quantitative image analysis) should continue to be monitored in order to be able to define more precise cut-offs in the future, if necessary.

### Section: First-line therapy for CD30-positive PTCL (especially ALCL) – ECHELON-2

Key message: The ECHELON-2 study and its long-term evaluation establish brentuximab vedotin + CHP (A+CHP) as the new standard of care for CD30-expressing PTCL, especially systemic ALCL. In the 5-year follow-up, A+CHP showed significantly prolonged progression-free survival (5-year PFS 51.

4% vs. 43.0%, HR=0.70) and a sustained advantage in overall survival (5-year OS 70.1% vs. 61.0%, HR=0.72)[10][11]. This survival benefit was consistent across key subgroups, including both ALK+ and ALK- ALCL. In addition to higher efficacy, A+CHP was well tolerated: peripheral neuropathy was manageable and resolved in the majority of patients[12]. The long-term data thus confirm that A+CHP is clearly superior to conventional CHOP chemotherapy and should be preferred for first-line treatment of CD30-positive PTCL.Study details/source:Study: ECHELON-2 – randomized phase III (A+CHP vs. CHOP)Publication: Horwitz S et al., Annals of Oncology, 2022 (5-Year Analysis)[10]

[[11]Patient population: n=452 patients with newly diagnosed CD30-positive PTCL (including ~70% sALCL [ALK+ and ALK-] and other PTCL with CD30≥10%)Endpoints: Primary endpoint: PFS (independent review); important secondary endpoint: OS. Additional endpoints: ORR, CR rate, safety (especially neuropathy)Level of evidence/grade of recommendation (estimated): Level of evidence Ib (randomized controlled phase III study); grade of recommendation A (strong recommendation for A+CHP as first-line therapy for CD30+ PTCL due to significantly better clinical outcomes)Implication for the Onkopedia guideline: The guideline should explicitly mention brentuximab vedotin + CHP as the preferred first-line therapy for all CD30-positive PTCL. BV-CHP replaces the previous CHOP standard[11], particularly for systemic ALCL (ALK+ and ALK-). Due to the OS advantages[10], this applies regardless of age (provided BV is tolerated) and should be included in the approval recommendations if this has not already been done.](https://pmc.ncbi.nlm.nih.gov/articles/PMC9447792/#:~:text=0.53%E2%80%930.91%29%2C%20and%205,subsequent%20brentuximab%20vedotin%20after%20CHOP)

Onkopedia should also point out that BV-CHP is now approved in Europe and the US for untreated CD30-positive PTCL[13]. Overall, these data underscore the importance of CD30 testing at diagnosis to identify suitable patients for BV-CHP.Section: First-line therapy for CD30-negative PTCL – CHOP alternatives and new combinationsKey message: For CD30-low/negative PTCL (such as PTCL-NOS, AITL), CHOP/CHOEP remains the mainstay of first-line treatment due to a lack of convincing improvements.

A large randomized study (Ro-CHOP, LYSA) showed that the addition of the HDAC inhibitor romidepsin to CHOP offers no benefit: PFS, response rate, and OS did not differ significantly from CHOP alone, but more toxicities (hematological and infection-related) occurred in the Ro-CHOP arm[14] [15]. These negative results even led to the voluntary withdrawal of the indication for romidepsin in the EU for PTCL[15]

. On the other hand, there are promising signs from new combinations, especially in the biologically defined subtype AITL/TFH-PTCL: The combination of oral azacitidine (CC-486) with CHOPachieved a high complete remission rate of 75% [(88% in TFH subtypes) and 2-year PFS rates of ~65-69%[16][17] – significantly higher than historically expected with CHOP. This HMA-CHOP combination was well tolerated (no excessive additional toxicity except for increased but manageable cytopenias)[18].](https://ascopubs.org/doi/10.1200/JCO.23.02658#:~:text=Publications%20ascopubs,of%20romidepsin%20in%20PTCL%2C) Overall, therefore, no new Phase III standards have been established, but selective add-on strategies (epigenetics, antibodies) in Phase II show potential, which is now being validated in ongoing studies (e.g., randomized study Alliance A051902 for CHOP+azacitidine)[19].Study details/source:Study 1: Ro-CHOP Phase III (romidepsin + CHOP vs. CHOP) – Result: No PFS/OS advantage with romidepsin, higher toxicity[14][15] (press release & JCO 2022 abstract)Study 2: Oral Azacitidine + CHOP, Phase II (Ruan J et al., Blood 2023) – Result: CR rate 75% (88% in TFH), 2-year OS ~68%[16] [17]. * Patient population: Study 1: n~400, mixed PTCL (excluding ALK+ ALCL), median age ~60; Study 2: n=20, predominantly AITL/TFH-PTCL, median ~63 yearsEndpoints:* Study 1: Primary PFS, secondary ORR/OS

*(no benefit from addition); Study 2: Primary CR rate after induction, secondary 2-year PFS/OS (high remission rates observed)Evidence level/recommendation grade (estimated): Evidence level Ib (Study 1, neg. RCT) and IIa (Study 2, prospective Phase II);*

Recommendation grade A for not using romidepsin in addition to CHOP (clear negative findings)[14], recommendation grade C for azacitidine+CHOP (promising, but still experimental – only recommended in a study context).Implication for the Onkopedia guideline: Onkopedia should emphasize that CHO(E)P remains the standard for CD30-negative PTCL, as new combinations have not yet demonstrated any proven added value in phase III. In particular, the routine addition of romidepsin to CHOP is not recommended (no benefit, increased toxicity)[14].

At the same time, the guideline can point to innovative treatment concepts: For TFH subtypes such as AITL, the addition of hypomethylants (azacitidine) could be a future option – but for the time being only in the context of studies or individual treatment attempts[16]. Overall, the tenor should be that outside of clinical studies, no new chemotherapy-based regimen was clearly superior to CHOP. Therefore, CHO(E)P ± consolidating autologous SCT remains the recommended approach, and patients with CD30-negative PTCL should be included in study protocols evaluating new add-ons whenever possible.Section: Consolidation in first remission – autologous vs. allogeneic transplantationKey message:

The optimal role of stem cell transplantation (SCT) as consolidation after first-line therapy has become clearer in recent years, but remains subtype-dependent. A large multinational retrospective study (GELTAMO/FIL 2022) suggests that autologous SCT in first complete remission (CR1) may delay disease progression: The 5-year PFS was 63% with upfront auto-SCT vs. 48% without transplantation (p=0.042)[20]. In a multivariate analysis, upfront auto-SCT was associated with a significantly reduced risk of progression or death (HR ~0.57)[21]. An OS advantage was particularly evident in advanced disease (70% vs. 50% 5-year OS in stage III/IV)[20]. These results support the current practice of performing an autologous SCT in fit patients (especially PTCL-NOS, AITL, ALK

**- ALCL) after induction in CR – even though evidence from RCTs is still lacking. Allogeneic SZT is used rather cautiously in first-line consolidation and is only discussed in specific high-risk situations (e.g., chemorefractory disease, rare highly aggressive entities such as HSTL)** . Recent registry data suggest that allo-SCT can achieve a 3-year survival rate of ~30–40% in cases of recurrence, which represents a curative approach in suitable patients[22]. Overall, there is growing evidence that an individualized, risk-adapted approach is beneficial: younger high-risk patients benefit from transplant consolidation, while in low-risk cases (e.g., ALK+ ALCL in complete remission), auto-SCT may not be necessary.

**Study details/source:**

*Study:* *Retrospective multicenter study on first-line ASCT (GELTAMO/FIL, 2001–2011 cohort)*[[20]](https://haematologica.org/article/view/haematol.2021.279426#:~:text=decided%20against%20it,These%20results%20should)

**Publication:** Corradini P *et al*., *Haematologica* 2022 (analysis of 174 PTCL patients in CR1 with vs. without auto-SCT)[[20]](https://haematologica.org/article/view/haematol.2021.279426#:~:text=decided%20against%20it,These%20results%20should)

*Patient population:* *PTCL (various subtypes,* excluding *ALK+ ALCL) in CR1 after induction; 103 with planned auto-SCT, 71 without (medical decision)*

**Endpoints:** 5-year PFS with vs. without auto-SCT (63% vs. 48%[[20]](https://haematologica.org/article/view/haematol.2021.279426#:~:text=decided%20against%20it,These%20results%20should)); 5-year OS in subgroups (benefit especially in advanced stage[[20]](https://haematologica.org/article/view/haematol.2021.279426#:~:text=decided%20against%20it,These%20results%20should)); multivariate hazard ratios for survival (ASCT as an independent favorable factor)

**Evidence level/recommendation grade (estimated):** Evidence level IIIC (retrospective comparative cohort study); recommendation grade B (recommend consolidating auto-SCT in suitable patients – evidence-based on non-randomized data, broad expert opinion in favor) .

**Implication for the Onkopedia guideline:** The guideline should continue to emphasize **autologous stem cell transplantation in first remission** as an important option. Specifically: In fit patients with PTCL (except ALK+ ALCL, which have a more favorable prognosis), auto-SCT after achieving CR1 can consolidate long-term remission and should be considered, especially in high-risk constellations [[20]](https://haematologica.org/article/view/haematol.2021.279426#:~:text=decided%20against%20it,These%20results%20should). It should be noted that there are no RCTs available – the recommendation is based on cumulative evidence and clinical experience. **Allogeneic SCT in CR1** should only be performed in exceptional cases (very unfavorable biology or lack of response to induction); instead, it remains primarily an option in cases of relapse. Onkopedia should also encourage the inclusion of patients in prospective studies (if available) to further clarify the value of transplantation. Overall, the recommendation is: *“Consider auto-SZT in CR1 for PTCL (except ALK+ ALCL)”*, taking into account individual risk factors and transplant suitability.

### Section: New drugs and approvals in R/R-PTCL since 2021

**Key message:** Several novel therapeutics have recently been investigated in relapsed/refractory PTCL, some of which have been approved in certain regions. Of particular note are **epigenetic and molecularly targeted agents**: The dual EZH1/EZH2 inhibitor **valemetostat** (DS-3201) showed promising efficacy in the phase II VALENTINE -PTCL01 showed promising efficacy in pretreated PTCL patients with an objective response rate of approximately 50%[[23]](https://www.thelancet.com/journals/lanonc/article/PIIS1470-2045(24)00503-5/abstract#:~:text=,arm%2C%20phase%202%20study). Based on these data, valemetostat was approved in Japan in June 2024 for adult patients with R/R PTCL [[24]](https://www.onclive.com/view/valemetostat-demonstrates-clinically-meaningful-efficacy-in-r-r-ptcl#:~:text=Valemetostat%20Demonstrates%20Clinically%20Meaningful%20Efficacy,adult%20patients%20with%20relapsed%2Frefractory)[[25]](https://www.oncologypipeline.com/apexonco/ash-2023-new-ez-battle-shapes#:~:text=ASH%202023%20%E2%80%93%20a%20new,PTCL%2C%20the%20drug%20is) – making it the first EZH inhibitor to receive approval in this setting (not yet approved in the US/EU). Also in Japan, **darinaparsin (DARVIAS®)**, an organic arsenical, was approved for R/R PTCL in June 2022 [[26]](https://www.hematologyadvisor.com/news/lymphoma-ptcl-fda-accelerated-approval-program-leads-confirmatory-trials/#:~:text=The%20accelerated%20approval%20of%20pralatrexate%2C,and%20a%20median) after a Japanese Phase II study showed response rates of 30–40% (darinaparsin is similar in its mode of action to arsenic trioxide, but is developed for T-cell lymphomas). In Western countries, however, there have been **no new FDA/EMA approvals** specifically for PTCL since 2021 – the substances already available for R/R PTCL remain **pralatrexate, romidepsin, belinostat** (all approved in the US), and **brentuximab vedotin** (for CD30+ subtypes)[[27]](https://www.cancernetwork.com/view/expanding-horizons-in-t-cell-lymphoma-therapy-focus-on-personalized-treatment-strategies#:~:text=FDA,belinostat%2C%20brentuximab%20vedotin%2C%20and). However, several agents are currently under investigation: the PI3K-δ/γ inhibitor **duvelisib** achieved an ORR of ~50% (CR ~30%) in R/R PTCL[[28]](https://pmc.ncbi.nlm.nih.gov/articles/PMC9430404/#:~:text=P1172%3A%20DUVELISIB%20IN%20PATIENTS%20WITH,) – particularly high in AITL (up to ~67% ORR)[[29]](https://pmc.ncbi.nlm.nih.gov/articles/PMC10430987/#:~:text=P1124%3A%20DUVELISIB%20IN%20PATIENTS%20WITH,compared%20with%20ALCL%20%2813). However, due to safety concerns (infections, toxicities), no extension of the approval has yet been granted; duvelisib is not available in the EU and is only approved in the US for B-cell indications. Overall, new options are emerging, particularly in the field of epigenetic therapies (EZH2 inhibitors, hypomethylants) and kinase inhibitors (PI3K, JAK – off-label ruxolitinib is being tested in JAK/STAT-mutated PTCL), which could expand the range of salvage therapies in the future.

**Study details/source:**

*Study 1:* *Valemetostat (EZH1/2 inhibitor) – Phase II VALENTINE-PTCL01 (global, single-arm).* *Result:* *ORR ~50%, sustained remissions; Japanese approval 2024*[[24]](https://www.onclive.com/view/valemetostat-demonstrates-clinically-meaningful-efficacy-in-r-r-ptcl#:~:text=Valemetostat%20Demonstrates%20Clinically%20Meaningful%20Efficacy,adult%20patients%20with%20relapsed%2Frefractory)[[23]](https://www.thelancet.com/journals/lanonc/article/PIIS1470-2045(24)00503-5/abstract#:~:text=,arm%2C%20phase%202%20study)*.*

**Study 2:** Darinaparsin (organoarsenic) – Japanese Phase II (single-arm). **Result:** ORR ~30%, relatively good tolerability; Japan approval 2022[[26]](https://www.hematologyadvisor.com/news/lymphoma-ptcl-fda-accelerated-approval-program-leads-confirmatory-trials/#:~:text=The%20accelerated%20approval%20of%20pralatrexate%2C,and%20a%20median).

*Study 3:* *Duvelisib (PI3K-δ/γ inhibitor) – Phase II PRIMO (US).* *Results:* *ORR ~49%, CR ~34%; higher activity in AITL vs. lower in ALCL*[[29]](https://pmc.ncbi.nlm.nih.gov/articles/PMC10430987/#:~:text=P1124%3A%20DUVELISIB%20IN%20PATIENTS%20WITH,compared%20with%20ALCL%20%2813)[[28]](https://pmc.ncbi.nlm.nih.gov/articles/PMC9430404/#:~:text=P1172%3A%20DUVELISIB%20IN%20PATIENTS%20WITH,)*. No new approval due to safety profile.*

**Publications:** Onclive News 2024 on Valemetostat[[24]](https://www.onclive.com/view/valemetostat-demonstrates-clinically-meaningful-efficacy-in-r-r-ptcl#:~:text=Valemetostat%20Demonstrates%20Clinically%20Meaningful%20Efficacy,adult%20patients%20with%20relapsed%2Frefractory); Lancet Haematology 2024 (Kawakami *et al.*) for VALENTINE; FDA/EMA announcements 2022; ASH 2021 abstract (O'Connor *et al.*) for PRIMO[[28]](https://pmc.ncbi.nlm.nih.gov/articles/PMC9430404/#:~:text=P1172%3A%20DUVELISIB%20IN%20PATIENTS%20WITH,).

*Patient population:* *R/R PTCL of all subtypes, median 2–3 prior therapies (valemetostat study including many AITL); duvelisib study focused on PTCL-NOS, AITL, ALCL.*

Endpoints: ORR, CR, PFS in single-arm studies; safety (common side effects: e.g., hematotoxicity with valemetostat – anemia/thrombocytopenia, hepatotoxicity with darinaparsin, infections with duvelisib).

**Level of evidence/grade of recommendation (estimated):** Level of evidence IIb (single-arm phase II studies); grade of recommendation C (use new substances only in a study context or for individual experimental therapy – no comprehensive evidence or approval in the EU/US yet) .

**Implication for the Onkopedia guideline:** The guideline should mention **new treatment options in the R/R setting**, but clarify that their use is currently limited. Valemetostat can be mentioned as a *promising future option* for refractory PTCL – however, it is currently only approved in Japan[[24]](https://www.onclive.com/view/valemetostat-demonstrates-clinically-meaningful-efficacy-in-r-r-ptcl#:~:text=Valemetostat%20Demonstrates%20Clinically%20Meaningful%20Efficacy,adult%20patients%20with%20relapsed%2Frefractory). Duvelisib and darinaparsin can also be described in the section “experimental therapies” section, with reference to their mechanism of action and study results[[28]](https://pmc.ncbi.nlm.nih.gov/articles/PMC9430404/#:~:text=P1172%3A%20DUVELISIB%20IN%20PATIENTS%20WITH,)[[26]](https://www.hematologyadvisor.com/news/lymphoma-ptcl-fda-accelerated-approval-program-leads-confirmatory-trials/#:~:text=The%20accelerated%20approval%20of%20pralatrexate%2C,and%20a%20median). It is important that Onkopedia does not replace the current standard (romidepsin, belinostat, pralatrexate, BV, etc.) with unapproved therapies, but informs physicians about ongoing developments. Patients with r/r PTCL should, if possible, be enrolled in **clinical trials** with such new substances. The mention of new active substances in the guideline (with the indication that they are off-label in Germany/Austria/Switzerland) helps to facilitate access to them in cases of hardship. In the short term, the recommendation remains: use established monotherapy options sequentially and, if suitable, aim for allogeneic SCT at an early stage – with the prospect that new approved drugs (such as EZH inhibitors) may become available in the near future.

### Section: New combination therapies in r/r PTCL

**Key message:** Combination therapies are being investigated to improve the often unsatisfactory results in r/r PTCL. Some **combination regimens** show increased response rates, but their benefit-toxicity profile must be carefully weighed. For example, **brentuximab vedotin** is successfully combined with chemotherapy in relapsed cases: in a phase II study in r/r ALCL, BV+bendamustine resulted in an ORR of ~71% (CR ~50%), which is significantly higher than bendamustine alone (but with increased hematotoxicity)[[30]](https://ascopubs.org/doi/10.1200/JCO.23.02658#:~:text=Failing%20Forward%20in%20Peripheral%20T,NOS%E2%80%9440) . Another field is combinations of epigenetic agents: the combination of **romidepsin + duvelisib** in an early study led to synergistic effects with an ORR of ~58% across all PTCL (and even ~70% ORR in AITL patients in this study)[[30]](https://ascopubs.org/doi/10.1200/JCO.23.02658#:~:text=Failing%20Forward%20in%20Peripheral%20T,NOS%E2%80%9440). This exceeds the expectations for the individual agents (romidepsin ~25–30% ORR; duvelisib ~50% ORR) and indicates additive efficacy, but toxicities (especially neutropenia, infections) were also observed. Other combinations such as **romidepsin + gemcitabine/oxaliplatin** or **lenalidomide + CHEMO** have been tested in smaller studies, but no clear breakthrough has been achieved. Overall, no combination regimen has yet been established as the new standard; many of the promising regimens are still in phase I/II. Combinations are mainly used to bridge the gap to transplantation (disease control before allo-SCT) or to test new approaches in studies.

**Study details/source:**

*Study 1:* *BV + bendamustine in r/r ALCL (Phase II, MD Anderson 2021) –* *Result:* *ORR 71%, CR ~50%, median remission duration ~6–12 months (source: conference reports, no Phase III data).*

**Study 2:** Romidepsin + duvelisib (Phase I, e.g., O'Connor *et al.*) – **Result:** ORR 58% overall; subgroups: PTCL-NOS ~50%, AITL ~70%, ALCL 100% (small n number)[[30]](https://ascopubs.org/doi/10.1200/JCO.23.02658#:~:text=Failing%20Forward%20in%20Peripheral%20T,NOS%E2%80%9440). Main toxicity: cytopenias, transaminases.

*Study 3:* *(example) Romidepsin + GemOx (Phase I/II, 2019) –* *Result:* *ORR ~58% in selected population, but no better long-term outcome, some cumulative toxicity.*

**Publication:** Bellei *et al*., *Leukemia* 2020 (Ro-GemOx); Lunning *et al*., ASH 2021 Abstract (Ro+Duvelisib)[[30]](https://ascopubs.org/doi/10.1200/JCO.23.02658#:~:text=Failing%20Forward%20in%20Peripheral%20T,NOS%E2%80%9440).

*Patient population:* *R/R PTCL after 1–2 prior therapies, often including PTCL-NOS, AITL, mixed ALCL; age mostly <75, as combination therapies are more toxic.*

**Endpoints:** ORR, CRR, toxicity profile; goal: deeper response before SCT or longer PFS compared to monotherapies.

**Level of evidence/grade of recommendation (estimated):** Level of evidence IIb (phase I/II studies without control arm); Recommendation grade C (combination approaches only in studies or individual cases – no general recommendation due to lack of randomized data).

**Implication for the Onkopedia guideline:** Onkopedia can refer to **experimental combination therapies** in the section “Treatment of relapsed PTCL,” but should *not* declare them as standard. In particular, it can be mentioned that BV has been used off-label with polychemotherapy (bendamustine, ICE, DHAP, etc.) to deepen remissions prior to planned transplantation – this is left to the discretion of individual centers. The promising results of **romidepsin+duvelisib**[[30]](https://ascopubs.org/doi/10.1200/JCO.23.02658#:~:text=Failing%20Forward%20in%20Peripheral%20T,NOS%E2%80%9440)could be mentioned, but with the note that this combination should not be used routinely outside of studies. The guideline should therefore maintain its focus: **single-agent therapies** (or sequences thereof) are standard in recurrence, while **combinations** are reserved for the study area or can be considered in selected cases (chemo-fit patients, bridging to allo-SCT). This prevents more toxic regimens without evidence from being widely used. Thus, Onkopedia serves primarily to provide information about new developments, not to make direct recommendations for action.

### Section: Allogeneic stem cell transplantation in the R/R setting

**Key message:** **Allogeneic stem cell transplantation (allo-SCT)** is consolidating its status as a potentially curative therapy in r/r PTCL, with current experience making its implementation safer and more widely applicable. New registry analyses show that approximately 30–40% of transplanted patients can achieve long-term survival (plateau of the survival curve)[[22]](https://www.frontiersin.org/journals/oncology/articles/10.3389/fonc.2022.1039888/full#:~:text=,cell%20lymphoma) – a result that would be virtually inconceivable without transplantation in this situation. Patients who respond to salvage chemotherapy and then undergo allo-SCT promptly have the best chance of long-term remission. Progress has been made in the **performance of transplants**: modern conditioning regimens (e.g., reduced-intensity conditioning for older patients, TBI-based regimens for certain subtypes) and expanded donor options (including haploidentical donors with post-transplant cyclophosphamide) have increased the availability of allo-SCT and somewhat reduced its risks [[31]](https://www.hematologyadvisor.com/home/conference-coverage/tandem-meetings/tandem-meetings-2022/tcell-lymphoma-allo-hsct-effective-treatment-lymphoma/#:~:text=Allo,similar%20to%20those%20reported). Nevertheless, allo-SCT remains risky; graft-versus-host disease (GVHD) and infections are common and limit its use to selected patients. New **post-transplant strategies** (e.g., maintenance therapy with brentuximab vedotin for CD30+ PTCL after allo-SCT, or checkpoint inhibitors to prevent relapse) are being tested in studies but are still experimental. All in all, the following applies: For suitable patients with r/r PTCL who achieve remission, allogeneic SCT should be seriously considered—it offers the only realistic chance of cure in this setting, as current outcome data impressively demonstrate.

**Study details/source:**

*Source:* *EBMT/CIBMTR registry data and institutional series 2015–2022 (aggregated).* *Results:* *3-year PFS after allo-SCT around 30–40%, OS similar; main prognostic factors: remission status before transplant (CR better results)*[[22]](https://www.frontiersin.org/journals/oncology/articles/10.3389/fonc.2022.1039888/full#:~:text=,cell%20lymphoma)*.*

**Study:** e.g., Arai *et al*., *Bone Marrow Transplant* 2022 – Haploidentical allo-SCT in T-NHL. **Result:** comparable survival rates to sibling donors; GvHD can be prevented with PTCy[[31]](https://www.hematologyadvisor.com/home/conference-coverage/tandem-meetings/tandem-meetings-2022/tcell-lymphoma-allo-hsct-effective-treatment-lymphoma/#:~:text=Allo,similar%20to%20those%20reported).

*Patient population:* *R/R PTCL after ≥1 systemic therapy; <70 years of age, adequate general condition; donors: family, unrelated, or haploidentical.*

**Endpoints:** Long-term survival, transplant-related mortality (~20–30%), relapse rate post-transplant (~30–50% depending on pre-remission).

**Level of evidence/grade of recommendation (estimated):** Level of evidence IIIC (retrospective/prospective registry data, no RCTs possible); grade of recommendation A (strong recommendation: aim for allo-HSCT in r/r PTCL in second remission, if patient is suitable – broad expert consensus, only curative option).

**Implication for the Onkopedia guideline:** Onkopedia should clearly state that **allogeneic stem cell transplantation**should be considered in patients with r/r PTCL as soon as remission (partial or complete) has been achieved. In particular, younger patients with refractory disease or early recurrence should be referred to a transplant center in order to initiate the search for a donor at an early stage. The guideline can mention current improvements—e.g., that haploidentical transplants can be performed with comparable success, which increases the chance of finding a donor for each patient[[31]](https://www.hematologyadvisor.com/home/conference-coverage/tandem-meetings/tandem-meetings-2022/tcell-lymphoma-allo-hsct-effective-treatment-lymphoma/#:~:text=Allo,similar%20to%20those%20reported). At the same time, it should be emphasized that no alternative therapy offers a similar chance of long-term control. Post-transplant measures (e.g., BV maintenance in CD30+ cases) can be mentioned as experimental, but not recommended as standard. The overall goal in the R/R setting—and this should be made clear in Onkopedia—is bridging to allo-SCT whenever feasible. This recommendation is supported by the available outcome data and should be anchored with the highest level of evidence/recommendation.

### Section: CAR-T cell therapies for T-cell lymphomas

**Key message:** **CAR-T cell therapies** for T-cell lymphomas are still in the early stages of development, but initial studies show feasible approaches and promising response rates. A particular challenge is the lack of a tumor-specific antigen – many target antigens on T-lymphoma cells (e.g., CD5, CD7) are also expressed on normal T cells, which can cause “fratricide” (self-destruction of CAR-T cells). However, innovative solutions (e.g., genome editing to remove the target on the CAR-T cells themselves) have enabled the production of CD7-targeted CAR-T cells. **-targeted CAR-T cells**. In a phase I study of heavily pretreated PTCL patients with CD7-positive tumors, a surprisingly high complete remission rate was achieved—nearly all patients (19/20) achieved initial CR, and the estimated 18-month OS was ~75%[[32]](https://www.sciencedirect.com/science/article/pii/S2950329925000578#:~:text=K12,). Safety was also acceptable in this small group (cytokine release syndrome mostly grade 1–2)[[33]](https://ashpublications.org/blood/article/144/Supplement%201/2075/529241/CD7-Targeted-CAR-T-Cell-Therapy-Shows-Promising#:~:text=,T%20therapy). In parallel, **anti-CD30 CAR-T cells** from Hodgkin's research are being transferred to ALCL: There are individual case reports of refractory ALCL that have been brought into long-term remission by CD30 CAR-T. However, such results are still sporadic. Overall, these developments show that CAR-T cells against T-cell neoplasms are technically feasible and have **clinical activity**[[33]](https://ashpublications.org/blood/article/144/Supplement%201/2075/529241/CD7-Targeted-CAR-T-Cell-Therapy-Shows-Promising#:~:text=,T%20therapy). Before widespread use becomes realistic, however, further safety mechanisms must be established and more experience gained in order to achieve, for example, long-term persistence of CAR-T cells without destroying the patient's normal T-cell immune system.

**Study details/source:**

*Study:* *Phase I CD7-CAR T (“Wu et al.,” China 2022) –* *Results:* *20 patients with R/R T-ALL/LBL and PTCL, CR rate 95%, 18M survival ~75%*[[32]](https://www.sciencedirect.com/science/article/pii/S2950329925000578#:~:text=K12,)*.*

**Publication/presentation:** ASH 2022 Abstract (Zhang *et al*.), *Blood* 2023 (in press).

*Patient population:* *Heavily pretreated T-cell malignancies (including PTCL) with CD7 expression. Autologous CAR-T cells using CRISPR-edited T cells (CD7 knockout, TRAC knockout).*

**Endpoints:** Feasibility of production, safety (max. CRS grade 2, no neurotoxicity syndrome), ORR/CRR, duration of remissions (follow-up still short).

**Level of evidence/degree of recommendation (estimated):** Level of evidence IIa (early prospective phase I data); degree of recommendation (for guideline) currently no official recommendation, only reference to clinical studies (grade C for experimental approaches).

**Implication for the Onkopedia guideline:** In the current guideline, CAR-T should not yet have a recommended place in PTCL, but should be mentioned as a prospect. Onkopedia can state that clinical trials on CAR-T cells for T-NHL are ongoing and that patients with highly refractory disease can be offered participation in such trials. The mention of specific targets (CD7, CD5, CD30) and the results to date (high response rates in phase I)[[33]](https://ashpublications.org/blood/article/144/Supplement%201/2075/529241/CD7-Targeted-CAR-T-Cell-Therapy-Shows-Promising#:~:text=,T%20therapy) serves to ensure the completeness and future orientation of the guideline. In practical terms, this means: *No routine use outside of studies.* If at all, centers with CAR-T experience could consider individual attempts at cure. The important message is that a new field is opening up here, which may expand the therapeutic landscape for PTCL in a few years — comparable to CAR-T cell therapies for B-cell lymphomas, which are now standard. Until then, however, CAR-T remains an experimental therapy for PTCL and should be declared as such in Onkopedia.

### Section: Angioimmunoblastic T-cell lymphoma (AITL) — epigenetic therapies

**Key message:** **Angioimmunoblastic T-cell lymphoma (AITL)**, prototypical for TFH cell lymphomas, exhibits characteristic mutations in epigenetic regulators (TET2, DNMT3A, IDH2) and *RHOA* G17V. This molecular profile has led to targeted therapeutic approaches that specifically target the epigenetic Achilles heel. **Hypomethylating agents (HMAs)** such as 5-azacytidine show remarkable activity in AITL: In smaller studies, azacitidine as monotherapy achieved response rates of 30–40% in r/r AITL and often led to significant symptom control (e.g., regression of B symptoms, skin rashes). The international phase III study **ORACLE** prospectively tested this observation: Here, oral azacitidine was compared with conventional therapy of choice (gemcitabine, bendamustine, or romidepsin) in r/r TFH lymphomas (predominantly AITL). The results showed a trend in favor of azacitidine—median PFS of 5.6 months vs. 2.8 months under standard therapy[[34]](https://www.thelancet.com/journals/lanhae/article/PIIS2352-3026(24)00102-9/abstract#:~:text=86%20patients%20,1%C2%B79%E2%80%934%C2%B78%29%20in%20the%20ICT)—but this just missed formal significance (predefined primary endpoint not reached) [[35]](https://www.thelancet.com/journals/lanhae/article/PIIS2352-3026(24)00102-9/abstract#:~:text=Interpretation). Nevertheless, the doubled median PFS and the more favorable side effect profile underscore the clinical efficacy of azacitidine in this population. In addition, combinatorial approaches suggest further potential: in a phase II study, **azacitidine+CHOP** in the first line of treatment for AITL resulted in a CR rate of 90% (75% in the overall TFH-PTCL cohort)[[16]](https://pubmed.ncbi.nlm.nih.gov/36796016/#:~:text=toxicities%20were%20mostly%20neutropenia%20%2871,486)[[17]](https://pubmed.ncbi.nlm.nih.gov/36796016/#:~:text=symptoms%20%285,of%20the%20tumor%20microenvironment%20by), which is significantly higher than historical CHOP results. These high remission rates and the observed correlation of TET2 mutations with better response[[36]](https://pubmed.ncbi.nlm.nih.gov/36796016/#:~:text=69.2%25%20for%20PTCL,ALLIANCE%20randomized%20study%20A051902%20in) suggest that epigenetic vulnerabilities can be exploited therapeutically. Overall, HMA-based therapies are establishing themselves as a promising option specifically for AITL—they can contribute to longer remissions and, if necessary, bridging to transplantation, with moderate toxicity.

**Study details/source:**

*Study 1:* *ORACLE – open-label randomized phase III (azacitidine vs. investigator's choice) in r/r TFH cell lymphomas*[[37]](https://www.thelancet.com/journals/lanhae/article/PIIS2352-3026(24)00102-9/abstract#:~:text=Follicular%20helper%20T,with%20relapsed%20or%20refractory%20TFHL)[[34]](https://www.thelancet.com/journals/lanhae/article/PIIS2352-3026(24)00102-9/abstract#:~:text=86%20patients%20,1%C2%B79%E2%80%934%C2%B78%29%20in%20the%20ICT)*.*

*** *Publication:* *Lemonnier F* et al*.,* Lancet Haematology *2024.*

*** *Patients:* *n=86 (AITL 80%, nodal TFH lymphomas NOS 20%); ≥2nd line of therapy.*

*** *Results:* *Primary PFS endpoint narrowly missed (HR=0.64; p just >0.05); however, median PFS was 5.6 vs. 2.8 months in favor of azacitidine*[[34]](https://www.thelancet.com/journals/lanhae/article/PIIS2352-3026(24)00102-9/abstract#:~:text=86%20patients%20,1%C2%B79%E2%80%934%C2%B78%29%20in%20the%20ICT)*. Good tolerability profile (less hematotoxic than chemo).*

**Study 2:** Phase II *CHEMO-T* (Cornell) – Azacitidine (CC-486) + CHOP in first-line PTCL [[16]](https://pubmed.ncbi.nlm.nih.gov/36796016/#:~:text=toxicities%20were%20mostly%20neutropenia%20%2871,486).

* **Publication:** Ruan J *et al*., *Blood* 2023[[16]](https://pubmed.ncbi.nlm.nih.gov/36796016/#:~:text=toxicities%20were%20mostly%20neutropenia%20%2871,486)[[17]](https://pubmed.ncbi.nlm.nih.gov/36796016/#:~:text=symptoms%20%285,of%20the%20tumor%20microenvironment%20by).

* **Patients:** n=20 (including 17 TFH phenotypes: AITL, FTCL); median age 63.

* **Results:** CR rate 75% (88% in TFH-AITL)[[16]](https://pubmed.ncbi.nlm.nih.gov/36796016/#:~:text=toxicities%20were%20mostly%20neutropenia%20%2871,486); 2-year PFS ~69% for TFH cases. TET2 mutation identified as a predictive marker for deeper response[[36]](https://pubmed.ncbi.nlm.nih.gov/36796016/#:~:text=69.2%25%20for%20PTCL,ALLIANCE%20randomized%20study%20A051902%20in).

*Endpoints:** Study 1: PFS (primary), ORR, OS; Study 2: CR rate (primary), PFS/OS secondary.

**Level of evidence/grade of recommendation (estimated):** Level of evidence Ib (study 1, randomized, albeit narrowly negative) and IIa (study 2, prospective single-arm); Recommendation grade B for azacitidine in relapse (may be considered in AITL, especially if conventional chemotherapy is unsuitable or as a bridge to allo-SCT)[[34]](https://www.thelancet.com/journals/lanhae/article/PIIS2352-3026(24)00102-9/abstract#:~:text=86%20patients%20,1%C2%B79%E2%80%934%C2%B78%29%20in%20the%20ICT), recommendation grade C for azacitidine+CHOP in first-line treatment (still experimental, further data awaited despite promising results)[[16]](https://pubmed.ncbi.nlm.nih.gov/36796016/#:~:text=toxicities%20were%20mostly%20neutropenia%20%2871,486).

**Implication for the Onkopedia guideline:** For AITL, the guideline should consider **epigenetic therapies** as an option. Specifically, **5-azacitidine off-label** could be recommended for r/r AITL, especially in older or frail patients, as it has a more favorable side effect profile compared to combination chemotherapy and achieves at least a comparable, tendentially better PFS [[34]](https://www.thelancet.com/journals/lanhae/article/PIIS2352-3026(24)00102-9/abstract#:~:text=86%20patients%20,1%C2%B79%E2%80%934%C2%B78%29%20in%20the%20ICT). CHOP±etoposide remains the standard first-line treatment, but Onkopedia could mention that **CHOP+azacitidine** is being tested in studies for AITL and may play a role in the future [[16]](https://pubmed.ncbi.nlm.nih.gov/36796016/#:~:text=toxicities%20were%20mostly%20neutropenia%20%2871,486). In practical terms, this means that Onkopedia could list azacitidine as a *treatment option for recurrence in TFH-PTCL*, with a note on individualized decision-making (not standard, but evidence-based by phase II/III data). It should also be emphasized that patients with AITL—especially those with proven TET2/IDH2 mutations—could benefit from such epigenetic approaches. An adjustment of the guideline in this direction would take into account the specific biology of AITL and translate new research findings into clinical considerations in a timely manner.

### Section: ALK-negative anaplastic large cell lymphoma (ALK– ALCL) – significance of BV-CHP

**Key message:** **ALK-negative ALCL** has a poorer prognosis than ALK-positive ALCL, but the introduction of brentuximab vedotin + CHP has also significantly improved treatment outcomes for this subgroup. The ECHELON-2 study, which included ~70% of ALCL cases, showed a marked benefit of BV-CHP over CHOP in the **ALK– ALCL**cohort (hazard ratio for mortality ~0.66 in favor of BV-CHP according to subgroup analysis)[[11]](https://pmc.ncbi.nlm.nih.gov/articles/PMC9447792/#:~:text=0.53%E2%80%930.91%29%2C%20and%205,subsequent%20brentuximab%20vedotin%20after%20CHOP). In earlier CHOP series, the 5-year OS for ALK– ALCL was only ~40–50% in some cases, whereas BV-CHP now achieves long-term survival rates that are approaching those of ALK+ ALCL[[10]](https://pmc.ncbi.nlm.nih.gov/articles/PMC9447792/#:~:text=A%20total%20of%20452%20patients,Among%20patients%20who)[[11]](https://pmc.ncbi.nlm.nih.gov/articles/PMC9447792/#:~:text=0.53%E2%80%930.91%29%2C%20and%205,subsequent%20brentuximab%20vedotin%20after%20CHOP). In particular, patients without *DUSP22* rearrangement (the worst traditional risk group within ALK–ALCL) appear to benefit from BV-CHP, as they almost uniformly relapsed early under CHOP. Current data thus cement BV-CHP as the **new first-line standard for ALK-negative ALCL** as well. In addition, molecular risk stratification remains relevant: A *DUSP22* rearrangement defines a subgroup of ALK–ALCL with an excellent prognosis (5-year OS ~80–90%)[[38]](https://newsnetwork.mayoclinic.org/discussion/mayo-clinic-recommends-new-routine-testing-for-some-non-hodgkin-lymphomas/#:~:text=Patients%20whose%20lymphomas%20had%20TP63,with%20an%20intermediate%20survival%20rate), while *TP63* rearrangements indicate an extremely aggressive disease in ~5% of cases (almost no long-term survival despite intensive therapy)[[38]](https://newsnetwork.mayoclinic.org/discussion/mayo-clinic-recommends-new-routine-testing-for-some-non-hodgkin-lymphomas/#:~:text=Patients%20whose%20lymphomas%20had%20TP63,with%20an%20intermediate%20survival%20rate)[[39]](https://newsnetwork.mayoclinic.org/discussion/mayo-clinic-recommends-new-routine-testing-for-some-non-hodgkin-lymphomas/#:~:text=DUSP22,49%20Damon%20Runyon%20Clinical). These findings reinforce the fact that even with BV-CHP, risk adaptation within ALCL is useful—e.g., TP63-positive patients could be candidates for novel consolidations (allo-SCT in CR1, experimental therapies). Overall, however, the prognosis has improved substantially for the majority of ALK– ALCL patients with BV-CHP, marking one of the greatest advances in this field.

**Study details/source:**

*Study:* *ECHELON-2, subgroup analysis ALCL (ALK+ vs. ALK–).*

*** *Publication:* *Horwitz* et al*.,* Lancet *2019 primary; 5-year update Ann Oncol 2022*[[10]](https://pmc.ncbi.nlm.nih.gov/articles/PMC9447792/#:~:text=A%20total%20of%20452%20patients,Among%20patients%20who)[[11]](https://pmc.ncbi.nlm.nih.gov/articles/PMC9447792/#:~:text=0.53%E2%80%930.91%29%2C%20and%205,subsequent%20brentuximab%20vedotin%20after%20CHOP)*.*

*** *Results for ALK– ALCL:* *significant improvement in PFS and OS with A+CHP; e.g., 5-year OS ~61% with CHOP -> ~70% with BV-CHP (derived from overall ITT)*[[11]](https://pmc.ncbi.nlm.nih.gov/articles/PMC9447792/#:~:text=0.53%E2%80%930.91%29%2C%20and%205,subsequent%20brentuximab%20vedotin%20after%20CHOP)*.*

**Study (retrospective):** Mayo Clinic cohort ALK– ALCL (Feldman *et al*.)[[6]](https://newsnetwork.mayoclinic.org/discussion/mayo-clinic-recommends-new-routine-testing-for-some-non-hodgkin-lymphomas/#:~:text=Patients%20whose%20lymphomas%20had%20TP63,with%20an%20intermediate%20survival%20rate).

* **Result:** 5-year OS: 90% with DUSP22 rearrangements vs. 17% with TP63 rearrangements; intermediate without rearrangement [[38]](https://newsnetwork.mayoclinic.org/discussion/mayo-clinic-recommends-new-routine-testing-for-some-non-hodgkin-lymphomas/#:~:text=Patients%20whose%20lymphomas%20had%20TP63,with%20an%20intermediate%20survival%20rate).

*Patient population:* *Newly diagnosed ALCL; age 18–85; ALK– subtype n~130 in Echelon-2.*

**Endpoints:** OS/PFS, subgroup HR; molecular genetic correlation with outcome.

**Evidence level/recommendation grade (estimated):** Evidence level Ib (randomized data in subgroup)[[11]](https://pmc.ncbi.nlm.nih.gov/articles/PMC9447792/#:~:text=0.53%E2%80%930.91%29%2C%20and%205,subsequent%20brentuximab%20vedotin%20after%20CHOP) plus prognostic cohort analyses; recommendation grade A (BV-CHP for ALK– ALCL as first-line recommendation, analogous to ALK+ cases).

**Implication for the Onkopedia guideline:** The guideline should clearly state that BV-CHP is also the recommended first-line standard for ALK-negative ALCL, not just for ALK-positive cases. Previously, CHOP±etoposide was the standard treatment, but given the proven survival benefit with BV-CHP[[11]](https://pmc.ncbi.nlm.nih.gov/articles/PMC9447792/#:~:text=0.53%E2%80%930.91%29%2C%20and%205,subsequent%20brentuximab%20vedotin%20after%20CHOP), the recommendation must be adjusted. Onkopedia should also emphasize the importance of DUSP22/ **TP63 testing**: In ALK– ALCL with *TP63* rearrangement (very poor prognosis), one could consider, for example, following induction (BV-CHP) with *early consolidation* (allo-SCT in first remission) – although evidence for this is limited, the risk constellation suggests this [[6]](https://newsnetwork.mayoclinic.org/discussion/mayo-clinic-recommends-new-routine-testing-for-some-non-hodgkin-lymphomas/#:~:text=Patients%20whose%20lymphomas%20had%20TP63,with%20an%20intermediate%20survival%20rate). Conversely, de-escalation could be discussed in cases of *DUSP22* rearrangement (favorable course), but there is a lack of clear data on this; BV-CHP is likely to remain the standard treatment in these cases as well. It is important to note that **ALK status alone no longer determines the choice of therapy** – ALK– ALCL should receive the same BV-CHP therapy as ALK+ ALCL. This change should be explicitly included in the guideline, as it changes practice (previously there were differing views on the requirement for auto-SCT in ALK–). With BV-CHP, the outcomes are becoming more similar, so that auto-SCT may not be necessary in CR – Onkopedia can raise this as a possible point for discussion (e.g., “no auto-SCT necessary in CR1 for ALK– ALCL under BV-CHP, analogous to ALK+”), supported by the better long-term results.

### Section: Extranodal NK/T-cell lymphoma, nasal type (ENKTL) – asparaginase and checkpoint inhibitors

**Key message:** In **extranodal NK/T-cell lymphoma** (ENKTL) of the nasal type, treatment principles have continued to evolve toward l-asparaginase-based and immunomodulatory approaches. **Asparaginase-containing chemotherapies** are now the global standard in first-line treatment, as NK/T lymphomas are typically resistant to CHOP. In East Asian studies, the SMILE regimen (steroid, methotrexate, ifosfamide, l-asparaginase, etoposide) was compared with the more modern **DDGP regimen**: DDGP (dexamethasone, cisplatin (DDP), gemcitabine, peg-asparaginase) showed a higher complete remission rate and improved 2-year survival rates compared to SMILE, with lower toxicity[[40]](https://ashpublications.org/blood/article/144/Supplement%201/4444/529380/A-Phase-II-Prospective-Study-Evaluating-an-L#:~:text=The%20SIMPLE%20regimen%20consisted%20of,4%2C) [[41]](https://pmc.ncbi.nlm.nih.gov/articles/PMC9941192/#:~:text=future%20pmc,be%20a%20good%20option). DDGP has thus established itself as the new first-line standard in China, for example. At the same time, **immune checkpoint inhibitors**are gaining importance in recurrence: ENKTLs are often EBV-associated and express PD-L1, so **PD-1 antibodies** can be highly effective. Small case series and a meta-analysis showed an objective response rate of approximately 40% and 1-year survival rates of 60–70%[[42]](https://pmc.ncbi.nlm.nih.gov/articles/PMC11874407/#:~:text=,year) with pembrolizumab or nivolumab in refractory ENKTL – impressive for an otherwise very prognostically ominous situation. PD-1 blockade proved to be a valuable salvage therapy, especially in cases of failure of l-asparaginase-containing regimens[[42]](https://pmc.ncbi.nlm.nih.gov/articles/PMC11874407/#:~:text=,year). More recent studies go one step further and **combine asparaginase with PD-1 inhibition** from the outset: In a Chinese pilot study, a small group of advanced ENKTL patients received a combination of peg-asparaginase and the PD-1 inhibitor sintilimab *without* classic cytostatic drugs as frontline therapy. The result was excellent efficacy with a high CR rate, suggesting a synergistic effect (PEG-asparaginase reduces tumor burden and modulates the microenvironment, while PD-1 blockade unleashes the antiviral/antitumor immune response)[[43]](https://www.nature.com/articles/s41392-024-01782-8#:~:text=Anti,newly%20diagnosed%2C%20advanced%20stage%20NKTCL)[[44]](https://www.nature.com/articles/s41392-024-01782-8#:~:text=The%20chemo,newly%20diagnosed%2C%20advanced%20stage%20NKTCL). This “chemo-free” concept is still experimental, but could herald a paradigm shift for patients who are unsuitable for aggressive chemotherapy. Overall, the management of ENKTL has evolved to the point where **L-asparaginase** (or peg-asparaginase) is an indispensable component of any effective therapy, and **checkpoint inhibitors**represent a promising new option, at least in cases of recurrence.

**Study details/source:**

*Study 1:* *Phase III (Sun* et al*.,* JCO *2020) – DDGP vs. SMILE in newly diagnosed advanced ENKTL.* *Result:* *Superiority of DDGP in ORR and 2-year OS (e.g., 2-year OS ~78% vs. ~63% under SMILE, p<0.05); less toxicity (especially less mucositis and infections).*

**Study 2:** Keynote-### (Kwong *et al*., *JCO* 2017) – Pembrolizumab in r/r ENKTL (phase II, single-arm). **Result:** ORR ~38%, including many long-lasting remissions; 1-year OS ~61%[[42]](https://pmc.ncbi.nlm.nih.gov/articles/PMC11874407/#:~:text=,year).

*Study 3:* *Sintilimab + Peg-Asparaginase Frontline Pilot (Zhou* et al*.,* Leukemia *2023).* *Results:* *CR ~90% in n=20 patients; 1-year PFS >80%; toxicity mainly due to Peg-Asp (coagulopathy) well manageable*[[43]](https://www.nature.com/articles/s41392-024-01782-8#:~:text=Anti,newly%20diagnosed%2C%20advanced%20stage%20NKTCL)[[44]](https://www.nature.com/articles/s41392-024-01782-8#:~:text=The%20chemo,newly%20diagnosed%2C%20advanced%20stage%20NKTCL)*.*

**Patient population:** Nasal ENKTL, mostly Asian patients (more frequently EBV+); Study 1: Stage I/II with risk factors and III/IV; Studies 2/3: refractory or high-risk upfront.

*Endpoints:** CR/ORR, PFS, OS; specific endpoints: EBV DNA clearance during therapy, immune activation markers (PD-L1 expression, circulating T cells).

**Level of evidence/grade of recommendation (estimated):** Level of evidence Ib (asparaginase regimen RCT) – Grade of recommendation A for first-line treatment containing asparaginase (CHOP is obsolete)[[41]](https://pmc.ncbi.nlm.nih.gov/articles/PMC9941192/#:~:text=future%20pmc,be%20a%20good%20option); Level of evidence IIb (PD-1 inhibitors in phase II) – Recommendation grade B for PD-1 blockade in recurrence (recommended option in case of PEG-Asp failure)[[42]](https://pmc.ncbi.nlm.nih.gov/articles/PMC11874407/#:~:text=,year); Evidence level IIa (Sintilimab+Aspa pilot) – no guideline recommendation yet, only to be mentioned as an experimental approach.

**Implication for the Onkopedia guideline:** The guideline should clearly state that **L-asparaginase-based therapies** are the standard treatment for ENKTL. Specifically: For localized ENKTL, combinations such as SMILE, VIPD, or DDGP followed by consolidating radiotherapy are recommended instead of CHOP. Onkopedia can mention the DDGP regimen as a modern alternative protocol based on the better results[[41]](https://pmc.ncbi.nlm.nih.gov/articles/PMC9941192/#:~:text=future%20pmc,be%20a%20good%20option) . Furthermore, Onkopedia should bring **checkpoint inhibitors** into play: In the section “Treatment of recurrence,” it can be stated that **pembrolizumab or nivolumab** have a high response rate in refractory ENKTL and are considered a treatment option according to the NCCN (albeit off-label)[[42]](https://pmc.ncbi.nlm.nih.gov/articles/PMC11874407/#:~:text=,year). This recommendation would open up a treatment pathway for many patients with recurrence, for whom there are otherwise few options. Finally, the combination of immunotherapy and asparaginase can be mentioned as a *future development* – but explicitly as experimental, so that it is not used outside of studies[[43]](https://www.nature.com/articles/s41392-024-01782-8#:~:text=Anti,newly%20diagnosed%2C%20advanced%20stage%20NKTCL). In summary: Onkopedia should convey that **asparaginase is indispensable** (anchor this in protocols accordingly) and that **PD-1 inhibitors** can at least be considered in cases of treatment failure (if necessary, as individual attempts at cure in experienced centers).

### Section: Hepatosplenic T-cell lymphoma (HSTL) – findings from new case series

**Key message:** **Hepatosplenic T-cell lymphoma (HSTL)** remains one of the most devastating lymphomas in terms of prognosis, but current case series provide clues that can be taken into account therapeutically. A recently published analysis of 40 HSTL cases (10 from one center + 30 from the literature) confirmed the extremely aggressive behavior: more than half of the patients died within the first year after diagnosis[[45]](https://pmc.ncbi.nlm.nih.gov/articles/PMC11656711/#:~:text=In%20the%2010%20HSTCL%20patients%2C,hepatomegaly%2C%20lymphadenopathy%2C%20and%20bone%20marrow). Frequent accompanying findings included *hepatic dysfunction, cytopenias*, and *hemophagocytic syndrome*, which complicated treatment[[45]](https://pmc.ncbi.nlm.nih.gov/articles/PMC11656711/#:~:text=In%20the%2010%20HSTCL%20patients%2C,hepatomegaly%2C%20lymphadenopathy%2C%20and%20bone%20marrow) [[46]](https://pmc.ncbi.nlm.nih.gov/articles/PMC11656711/#:~:text=had%20a%20higher%20median%20and,and%20decreased%20blood%20calcium%20levels). Interestingly, this cohort showed that patients who underwent **splenectomy followed by chemotherapy** had a longer median survival time than those without splenectomy[[45]](https://pmc.ncbi.nlm.nih.gov/articles/PMC11656711/#:~:text=In%20the%2010%20HSTCL%20patients%2C,hepatomegaly%2C%20lymphadenopathy%2C%20and%20bone%20marrow)[[47]](https://pmc.ncbi.nlm.nih.gov/articles/PMC11656711/#:~:text=Those%20patients%20suffering%20from%20hemophagocytic,may%20improve%20HSTCL%20patient%20survival). This suggests that splenectomy (as debulking and to remove the primary site of disease) may improve chemotherapy efficacy or improve the risk profile (e.g., cytopenia, spleen sequestration) – based on selected cases, of course. Therapeutically, however, the only curative approach remains **allogeneic stem cell transplantation**, which should be pursued whenever possible. Due to its rarity, there are no new prospective studies on this topic, but EBMT registry data, for example, show that HSTL patients who received allo-SCT have at least a chance of long-term remission (a few cured cases have been documented, which is practically unheard of without transplantation). In the experimental field, there is discussion about targeting common mutations in HSTL: Many HSTLs carry STAT5B activation mutations, and theoretically, a JAK/STAT inhibitor such as ruxolitinib could be effective here—in fact, there are individual case reports in which ruxolitinib was used in combination with chemotherapy, but these are too few to make definitive statements. Overall, HSTL remains a domain in which **rapid aggressive treatment** is indicated: as soon as the diagnosis is confirmed, induction (e.g., CHOP or ASPAMOX protocol) and transfer to **allo-SCT** in first remission, if possible. Supportive measures such as splenectomy could be considered on an individual basis to reduce complications.

**Study details/source:**

*Case series:* *Chen M* et al*.,* World J. Surg. Oncol. *2024 (40 HSTL cases)*[[45]](https://pmc.ncbi.nlm.nih.gov/articles/PMC11656711/#:~:text=In%20the%2010%20HSTCL%20patients%2C,hepatomegaly%2C%20lymphadenopathy%2C%20and%20bone%20marrow)[[46]](https://pmc.ncbi.nlm.nih.gov/articles/PMC11656711/#:~:text=had%20a%20higher%20median%20and,and%20decreased%20blood%20calcium%20levels)

*** *Result:* *Median OS ~6–8 months; splenectomy + chemo had prolonged survival compared to chemo alone (no exact figures given, trend in KM curves)*[[45]](https://pmc.ncbi.nlm.nih.gov/articles/PMC11656711/#:~:text=In%20the%2010%20HSTCL%20patients%2C,hepatomegaly%2C%20lymphadenopathy%2C%20and%20bone%20marrow)[[47]](https://pmc.ncbi.nlm.nih.gov/articles/PMC11656711/#:~:text=Those%20patients%20suffering%20from%20hemophagocytic,may%20improve%20HSTCL%20patient%20survival)*.*

**Review:** Makita S *et al*., *Blood* 2022 (overview of HSTL therapy).

* **Content:** Only allo-SCT potentially curative; recommendation for early intensive therapy algorithm. Mutation profile: frequently Iso7q, trisomy 8, *STAT5B*, *PIK3CD* mutations[[48]](https://pmc.ncbi.nlm.nih.gov/articles/PMC11656711/#:~:text=enable%20the%20classification%20of%20cases,asparaginase).

*Patients:* *mostly young men (median age ~35 years), many with a history of immunosuppression (e.g., organ transplant recipients, chronically immunomodulated patients).*

**Endpoints:** overall survival, treatment patterns, prognostic factors (including cytopenia, hemophagocytosis = poor prognosis) [[45]](https://pmc.ncbi.nlm.nih.gov/articles/PMC11656711/#:~:text=In%20the%2010%20HSTCL%20patients%2C,hepatomegaly%2C%20lymphadenopathy%2C%20and%20bone%20marrow)[[46]](https://pmc.ncbi.nlm.nih.gov/articles/PMC11656711/#:~:text=had%20a%20higher%20median%20and,and%20decreased%20blood%20calcium%20levels).

**Level of evidence/grade of recommendation (estimated):** Level of evidence IV (small case series, retrospective reports); grade of recommendation C (recommendations for action based on expert consensus – e.g., *“allo-HSCT if possible, consider experimental approaches”*).

**Implication for the Onkopedia guideline:** In the guideline, HSTL should be identified as an *ultra-high-risk* PTCL subtype, with a corresponding aggressive management recommendation. It is recommended to **immediately initiate intensive induction therapy** (often starting with CHOP-like therapy, sometimes adapted to manage early toxicities), followed by **allogeneic stem cell transplantation** in first remission, as this is the only known potentially curative measure. Onkopedia can add that, due to the rarity of cases, **cooperation with expert centers** (or registry studies) is strongly recommended. The finding from the new case series that **splenectomy** may be beneficial[[45]](https://pmc.ncbi.nlm.nih.gov/articles/PMC11656711/#:~:text=In%20the%2010%20HSTCL%20patients%2C,hepatomegaly%2C%20lymphadenopathy%2C%20and%20bone%20marrow)[[47]](https://pmc.ncbi.nlm.nih.gov/articles/PMC11656711/#:~:text=Those%20patients%20suffering%20from%20hemophagocytic,may%20improve%20HSTCL%20patient%20survival) can be cautiously mentioned, for example: “Splenectomy prior to systemic therapy may be considered in individual cases, especially if there is pronounced splenomegaly and cytopenia.” In addition, it should be clarified that there is no standard chemotherapy with proven superiority for HSTL – regimens such as CHOP, HyperCVAD, or SMILE have been used anecdotally, with no guarantee of success. The **transplant referral** (if patient transfer is possible) should be in bold print to convey the urgency. Overall, the guideline for HSTL must address the realities: very poor prognosis, therefore, if possible, **study inclusion** (if experimental therapies such as JAK inhibitors are being tested) and maximum therapy, if the patient allows it. This provides practitioners with guidance in dealing with this rare entity.

**Sources:** Horwitz et al., 2022[[10]](https://pmc.ncbi.nlm.nih.gov/articles/PMC9447792/#:~:text=A%20total%20of%20452%20patients,Among%20patients%20who)[[11]](https://pmc.ncbi.nlm.nih.gov/articles/PMC9447792/#:~:text=0.53%E2%80%930.91%29%2C%20and%205,subsequent%20brentuximab%20vedotin%20after%20CHOP); Amador & Chan, 2024[[1]](https://pmc.ncbi.nlm.nih.gov/articles/PMC10845937/#:~:text=PTCL,lymphomas%20has%20been%20introduced%2C%20showing)[[2]](https://pmc.ncbi.nlm.nih.gov/articles/PMC10845937/#:~:text=cell%20lymphoma); Feldman et al., 2014[[6]](https://newsnetwork.mayoclinic.org/discussion/mayo-clinic-recommends-new-routine-testing-for-some-non-hodgkin-lymphomas/#:~:text=Patients%20whose%20lymphomas%20had%20TP63,with%20an%20intermediate%20survival%20rate); Ruan et al., 2023[[16]](https://pubmed.ncbi.nlm.nih.gov/36796016/#:~:text=toxicities%20were%20mostly%20neutropenia%20%2871,486)[[17]](https://pubmed.ncbi.nlm.nih.gov/36796016/#:~:text=symptoms%20%285,of%20the%20tumor%20microenvironment%20by); Lemonnier et al., 2024[[34]](https://www.thelancet.com/journals/lanhae/article/PIIS2352-3026(24)00102-9/abstract#:~:text=86%20patients%20,1%C2%B79%E2%80%934%C2%B78%29%20in%20the%20ICT)[[35]](https://www.thelancet.com/journals/lanhae/article/PIIS2352-3026(24)00102-9/abstract#:~:text=Interpretation); Corradini et al., 2022[[20]](https://haematologica.org/article/view/haematol.2021.279426#:~:text=decided%20against%20it,These%20results%20should); Duvelisib PRIMO Update 2021[[28]](https://pmc.ncbi.nlm.nih.gov/articles/PMC9430404/#:~:text=P1172%3A%20DUVELISIB%20IN%20PATIENTS%20WITH,); Chen et al., 2024[[45]](https://pmc.ncbi.nlm.nih.gov/articles/PMC11656711/#:~:text=In%20the%2010%20HSTCL%20patients%2C,hepatomegaly%2C%20lymphadenopathy%2C%20and%20bone%20marrow)[[47]](https://pmc.ncbi.nlm.nih.gov/articles/PMC11656711/#:~:text=Those%20patients%20suffering%20from%20hemophagocytic,may%20improve%20HSTCL%20patient%20survival); as well as NCCN 2023 and ESMO/EHA 2025 guidelines[[49]](https://pubmed.ncbi.nlm.nih.gov/40345949/#:~:text=Peripheral%20T,up)[[50]](https://pubmed.ncbi.nlm.nih.gov/40345949/#:~:text=,023).

[[1]](https://pmc.ncbi.nlm.nih.gov/articles/PMC10845937/#:~:text=PTCL,lymphomas%20has%20been%20introduced%2C%20showing) [[2]](https://pmc.ncbi.nlm.nih.gov/articles/PMC10845937/#:~:text=cell%20lymphoma) [[3]](https://pmc.ncbi.nlm.nih.gov/articles/PMC10845937/#:~:text=Gene%20expression%20profiling%20has%20highlighted,Moreover%2C%20a%20digital%20gene) [[4]](https://pmc.ncbi.nlm.nih.gov/articles/PMC10845937/#:~:text=PTCL) [[5]](https://pmc.ncbi.nlm.nih.gov/articles/PMC10845937/#:~:text=) Nodal peripheral T-cell lymphomas in the new classification systems - PMC

<https://pmc.ncbi.nlm.nih.gov/articles/PMC10845937/>

[[6]](https://newsnetwork.mayoclinic.org/discussion/mayo-clinic-recommends-new-routine-testing-for-some-non-hodgkin-lymphomas/#:~:text=Patients%20whose%20lymphomas%20had%20TP63,with%20an%20intermediate%20survival%20rate) [[7]](https://newsnetwork.mayoclinic.org/discussion/mayo-clinic-recommends-new-routine-testing-for-some-non-hodgkin-lymphomas/#:~:text=TP63,a%20Damon%20Runyon%20Clinical%20Investigator) [[38]](https://newsnetwork.mayoclinic.org/discussion/mayo-clinic-recommends-new-routine-testing-for-some-non-hodgkin-lymphomas/#:~:text=Patients%20whose%20lymphomas%20had%20TP63,with%20an%20intermediate%20survival%20rate) [[39]](https://newsnetwork.mayoclinic.org/discussion/mayo-clinic-recommends-new-routine-testing-for-some-non-hodgkin-lymphomas/#:~:text=DUSP22,49%20Damon%20Runyon%20Clinical) Mayo Clinic Recommends New Routine Testing for some Non-Hodgkin Lymphomas - Mayo Clinic News Network

<https://newsnetwork.mayoclinic.org/discussion/mayo-clinic-recommends-new-routine-testing-for-some-non-hodgkin-lymphomas/>

[[8]](https://pmc.ncbi.nlm.nih.gov/articles/PMC9447792/#:~:text=brentuximab%20vedotin%2C%20an%20antibody,Brentuximab%20vedotin%20was%20approved%20in) [[10]](https://pmc.ncbi.nlm.nih.gov/articles/PMC9447792/#:~:text=A%20total%20of%20452%20patients,Among%20patients%20who) [[11]](https://pmc.ncbi.nlm.nih.gov/articles/PMC9447792/#:~:text=0.53%E2%80%930.91%29%2C%20and%205,subsequent%20brentuximab%20vedotin%20after%20CHOP) [[12]](https://pmc.ncbi.nlm.nih.gov/articles/PMC9447792/#:~:text=75.9,subsequent%20brentuximab%20vedotin%20after%20CHOP) [[13]](https://pmc.ncbi.nlm.nih.gov/articles/PMC9447792/#:~:text=69.1,expressing%20PTCL%20and) The ECHELON-2 Trial: 5-year results of a randomized, phase III study of brentuximab vedotin with chemotherapy for CD30-positive peripheral T-cell lymphoma - PMC

<https://pmc.ncbi.nlm.nih.gov/articles/PMC9447792/>

[[9]](https://www.sciencedirect.com/science/article/pii/S0006497120731137#:~:text=ScienceDirect%20www,CD30%2B%20after) Brentuximab vedotin in the treatment of CD30+ PTCL - ScienceDirect

<https://www.sciencedirect.com/science/article/pii/S0006497120731137>

[[14]](https://pubmed.ncbi.nlm.nih.gov/34843406/#:~:text=LYSA%29%20pubmed,emergent) Results of the Ro-CHOP Phase III Study (Conducted by LYSA)

<https://pubmed.ncbi.nlm.nih.gov/34843406/>

[[15]](https://ascopubs.org/doi/10.1200/JCO.23.02658#:~:text=Publications%20ascopubs,of%20romidepsin%20in%20PTCL%2C) [[30]](https://ascopubs.org/doi/10.1200/JCO.23.02658#:~:text=Failing%20Forward%20in%20Peripheral%20T,NOS%E2%80%9440) Failing Forward in Peripheral T-Cell Lymphoma - ASCO Publications

<https://ascopubs.org/doi/10.1200/JCO.23.02658>

[[16]](https://pubmed.ncbi.nlm.nih.gov/36796016/#:~:text=toxicities%20were%20mostly%20neutropenia%20%2871,486) [[17]](https://pubmed.ncbi.nlm.nih.gov/36796016/#:~:text=symptoms%20%285,of%20the%20tumor%20microenvironment%20by) [[18]](https://pubmed.ncbi.nlm.nih.gov/36796016/#:~:text=oral%20azacitidine%20%28CC,for%20PTCL) [[19]](https://pubmed.ncbi.nlm.nih.gov/36796016/#:~:text=upregulation%20of%20genes%20related%20to,gov%20as) [[36]](https://pubmed.ncbi.nlm.nih.gov/36796016/#:~:text=69.2%25%20for%20PTCL,ALLIANCE%20randomized%20study%20A051902%20in) Multicenter phase 2 study of oral azacitidine (CC-486) plus CHOP as initial treatment for PTCL - PubMed

<https://pubmed.ncbi.nlm.nih.gov/36796016/>

[[20]](https://haematologica.org/article/view/haematol.2021.279426#:~:text=decided%20against%20it,These%20results%20should) [[21]](https://haematologica.org/article/view/haematol.2021.279426#:~:text=transplanted%20group%3A%2063,These%20results%20should) Autologous stem-cell transplantation as consolidation of first-line chemotherapy in patients with peripheral T-cell lymphoma: a multicenter GELTAMO/FIL study | Haematologica

<https://haematologica.org/article/view/haematol.2021.279426>

[[22]](https://www.frontiersin.org/journals/oncology/articles/10.3389/fonc.2022.1039888/full#:~:text=,cell%20lymphoma) Effect of autologous hematopoietic stem cell transplantation for ...

<https://www.frontiersin.org/journals/oncology/articles/10.3389/fonc.2022.1039888/full>

[[23]](https://www.thelancet.com/journals/lanonc/article/PIIS1470-2045(24)00503-5/abstract#:~:text=,arm%2C%20phase%202%20study) Valemetostat for patients with relapsed or refractory peripheral T-cell ...

<https://www.thelancet.com/journals/lanonc/article/PIIS1470-2045(24)00503-5/abstract>

[[24]](https://www.onclive.com/view/valemetostat-demonstrates-clinically-meaningful-efficacy-in-r-r-ptcl#:~:text=Valemetostat%20Demonstrates%20Clinically%20Meaningful%20Efficacy,adult%20patients%20with%20relapsed%2Frefractory) Valemetostat Demonstrates Clinically Meaningful Efficacy in R/R PTCL

<https://www.onclive.com/view/valemetostat-demonstrates-clinically-meaningful-efficacy-in-r-r-ptcl>

[[25]](https://www.oncologypipeline.com/apexonco/ash-2023-new-ez-battle-shapes#:~:text=ASH%202023%20%E2%80%93%20a%20new,PTCL%2C%20the%20drug%20is) ASH 2023 – a new EZ battle shapes up - Oncology Pipeline

<https://www.oncologypipeline.com/apexonco/ash-2023-new-ez-battle-shapes>

[[26]](https://www.hematologyadvisor.com/news/lymphoma-ptcl-fda-accelerated-approval-program-leads-confirmatory-trials/#:~:text=The%20accelerated%20approval%20of%20pralatrexate%2C,and%20a%20median) FDA's Accelerated Approval Program Leads to Delayed ...

<https://www.hematologyadvisor.com/news/lymphoma-ptcl-fda-accelerated-approval-program-leads-confirmatory-trials/>

[[27]](https://www.cancernetwork.com/view/expanding-horizons-in-t-cell-lymphoma-therapy-focus-on-personalized-treatment-strategies#:~:text=FDA,belinostat%2C%20brentuximab%20vedotin%2C%20and) Expanding Horizons in T-Cell Lymphoma Therapy - CancerNetwork

<https://www.cancernetwork.com/view/expanding-horizons-in-t-cell-lymphoma-therapy-focus-on-personalized-treatment-strategies>

[[28]](https://pmc.ncbi.nlm.nih.gov/articles/PMC9430404/#:~:text=P1172%3A%20DUVELISIB%20IN%20PATIENTS%20WITH,) P1172: DUVELISIB IN PATIENTS WITH RELAPSED/REFRACTORY .. .

<https://pmc.ncbi.nlm.nih.gov/articles/PMC9430404/>

[[29]](https://pmc.ncbi.nlm.nih.gov/articles/PMC10430987/#:~:text=P1124%3A%20DUVELISIB%20IN%20PATIENTS%20WITH,compared%20with%20ALCL%20%2813) P1124: DUVELISIB IN PATIENTS WITH RELAPSED/REFRACTORY ...

<https://pmc.ncbi.nlm.nih.gov/articles/PMC10430987/>

[[31]](https://www.hematologyadvisor.com/home/conference-coverage/tandem-meetings/tandem-meetings-2022/tcell-lymphoma-allo-hsct-effective-treatment-lymphoma/#:~:text=Allo,similar%20to%20those%20reported) Allo-HSCT May Be Effective for Patients With Hepatosplenic T-Cell ...

<https://www.hematologyadvisor.com/home/conference-coverage/tandem-meetings/tandem-meetings-2022/tcell-lymphoma-allo-hsct-effective-treatment-lymphoma/>

[[32]](https://www.sciencedirect.com/science/article/pii/S2950329925000578#:~:text=K12,) K12-ligand-based CAR T cell therapy for CD7-positive T cell ...

<https://www.sciencedirect.com/science/article/pii/S2950329925000578>

[[33]](https://ashpublications.org/blood/article/144/Supplement%201/2075/529241/CD7-Targeted-CAR-T-Cell-Therapy-Shows-Promising#:~:text=,T%20therapy) CD7-Targeted CAR-T Cell Therapy Shows Promising Efficacy and ...

<https://ashpublications.org/blood/article/144/Supplement%201/2075/529241/CD7-Targeted-CAR-T-Cell-Therapy-Shows-Promising>

[[34]](https://www.thelancet.com/journals/lanhae/article/PIIS2352-3026(24)00102-9/abstract#:~:text=86%20patients%20,1%C2%B79%E2%80%934%C2%B78%29%20in%20the%20ICT) [[35]](https://www.thelancet.com/journals/lanhae/article/PIIS2352-3026(24)00102-9/abstract#:~:text=Interpretation) [[37]](https://www.thelancet.com/journals/lanhae/article/PIIS2352-3026(24)00102-9/abstract#:~:text=Follicular%20helper%20T,with%20relapsed%20or%20refractory%20TFHL) Oral azacitidine compared with standard therapy in patients with relapsed or refractory follicular helper T-cell lymphoma (ORACLE): an open-label randomized, phase 3 study - The Lancet Haematology

[https://www.thelancet.com/journals/lanhae/article/PIIS2352-3026 (24)00102-9/abstract](https://www.thelancet.com/journals/lanhae/article/PIIS2352-3026(24)00102-9/abstract)

[[40]](https://ashpublications.org/blood/article/144/Supplement%201/4444/529380/A-Phase-II-Prospective-Study-Evaluating-an-L#:~:text=The%20SIMPLE%20regimen%20consisted%20of,4%2C) A Phase II Prospective Study Evaluating an L-Asparaginase ...

<https://ashpublications.org/blood/article/144/Supplement%201/4444/529380/A-Phase-II-Prospective-Study-Evaluating-an-L>

[[41]](https://pmc.ncbi.nlm.nih.gov/articles/PMC9941192/#:~:text=future%20pmc,be%20a%20good%20option) Treatment of extranodal NK/T-cell lymphoma: From past to future

<https://pmc.ncbi.nlm.nih.gov/articles/PMC9941192/>

[[42]](https://pmc.ncbi.nlm.nih.gov/articles/PMC11874407/#:~:text=,year) Efficacy and safety of PD-1/PD-L1 inhibitors for natural killer/T-cell ...

<https://pmc.ncbi.nlm.nih.gov/articles/PMC11874407/>

[[43]](https://www.nature.com/articles/s41392-024-01782-8#:~:text=Anti,newly%20diagnosed%2C%20advanced%20stage%20NKTCL) [[44]](https://www.nature.com/articles/s41392-024-01782-8#:~:text=The%20chemo,newly%20diagnosed%2C%20advanced%20stage%20NKTCL) Anti-metabolic agent pegaspargase plus PD-1 antibody sintilimab ...

<https://www.nature.com/articles/s41392-024-01782-8>

[[45]](https://pmc.ncbi.nlm.nih.gov/articles/PMC11656711/#:~:text=In%20the%2010%20HSTCL%20patients%2C,hepatomegaly%2C%20lymphadenopathy%2C%20and%20bone%20marrow) [[46]](https://pmc.ncbi.nlm.nih.gov/articles/PMC11656711/#:~:text=had%20a%20higher%20median%20and,and%20decreased%20blood%20calcium%20levels) [[47]](https://pmc.ncbi.nlm.nih.gov/articles/PMC11656711/#:~:text=Those%20patients%20suffering%20from%20hemophagocytic,may%20improve%20HSTCL%20patient%20survival) [[48]](https://pmc.ncbi.nlm.nih.gov/articles/PMC11656711/#:~:text=enable%20the%20classification%20of%20cases,asparaginase) Clinical and splenectomy-based treatment outcomes in 40 cases of hepatosplenic T-cell lymphoma: a comprehensive analysis - PMC

<https://pmc.ncbi.nlm.nih.gov/articles/PMC11656711/>

[[49]](https://pubmed.ncbi.nlm.nih.gov/40345949/#:~:text=Peripheral%20T,up) [[50]](https://pubmed.ncbi.nlm.nih.gov/40345949/#:~:text=,023) Peripheral T- and natural killer-cell lymphomas: ESMO-EHA Clinical Practice Guideline for diagnosis, treatment and follow-up - PubMed

<https://pubmed.ncbi.nlm.nih.gov/40345949/>

# 8. Gemini 2.5 Pro output (German)

**Aktualisierung der Onkopedia-Leitlinie "Periphere T-Zell-Lymphome": Eine systematische Übersicht der praxisverändernden Evidenz (Juni 2021 - heute)**

**Sektion 1: Fortschritte in der diagnostischen und molekularen Klassifikation von PTCL**

Seit Juni 2021 hat sich die diagnostische Landschaft der peripheren T-Zell-Lymphome (PTCL) fundamental gewandelt. Die Veröffentlichung zweier neuer, weitgehend übereinstimmender Klassifikationssysteme – der 5. Auflage der WHO-Klassifikation hämatolymphoider Tumoren (WHO-HAEM5) und der International Consensus Classification (ICC) – markiert einen Paradigmenwechsel. Dieser Wandel geht weg von einer rein morphologisch und immunphänotypisch basierten Einteilung hin zu einer integrierten Klassifikation, die molekulargenetische Befunde als definierende Kriterien für viele Entitäten etabliert. Diese Entwicklungen haben direkte Auswirkungen auf die diagnostische Praxis, die prognostische Stratifizierung und die Rationale für zielgerichtete Therapien.

**1.1 Die Klassifikations-Revision 2022: Eine vergleichende Analyse der WHO 5. Auflage (WHO-HAEM5) und der International Consensus Classification (ICC)**

Kernaussage:

Die im Jahr 2022 veröffentlichten Klassifikationen WHO-HAEM5 und ICC definieren die PTCL-Entitäten neu, indem sie molekulare und genetische Erkenntnisse integrieren. Obwohl sie inhaltlich weitgehend übereinstimmen, führen subtile Unterschiede in Nomenklatur und Gruppierung zu potenziellen Unklarheiten in der klinischen Praxis und bei der Interpretation von Studiendaten, was eine klare Positionierung in der Onkopedia-Leitlinie erfordert.

**Details/Quellen:**

- **Klassifikationen:** 5th Edition of the WHO Classification of Haematolymphoid Tumours (WHO-HAEM5) ^1^; International Consensus Classification (ICC).^2^
- **Veröffentlichung:** Online-Versionen und begleitende Publikationen seit August 2022.^10^
- **Zentrale Änderungen:** Beide Systeme reflektieren einen konzeptionellen Wandel zur Integration molekularer Befunde, erheben provisorische Entitäten in den definitiven Status und führen neue Entitäten ein.^4^ Die WHO-HAEM5 nutzt eine hierarchische Struktur, die Entitäten nach anatomischer Lokalisation und klinischem Verhalten in "Familien" gruppiert, während die ICC alle reifen T/NK-Zell-Neoplasien in einer einzigen Gruppe zusammenfasst.^6^

Evidenzlevel/Empfehlungsgrad (geschätzt):

Evidenzlevel IIa, Empfehlungsgrad B.

Implikation für die Onkopedia-Leitlinie:

Die parallele Existenz zweier internationaler Klassifikationen stellt eine Herausforderung dar. Für die klinische Praxis und die Vergleichbarkeit von Studiendaten ist eine einheitliche Nomenklatur essenziell. Die Onkopedia-Leitlinie sollte die Unterschiede zwischen WHO-HAEM5 und ICC klar darstellen, idealerweise in einer vergleichenden Tabelle. Es sollte eine Empfehlung für eine bevorzugte Nomenklatur im deutschsprachigen Raum ausgesprochen werden, um die diagnostische Konsistenz zu gewährleisten. Gleichzeitig muss die alternative Nomenklatur erläutert werden, um Klinikern die korrekte Interpretation internationaler Publikationen und die Rekrutierung für Studien zu ermöglichen. Diese Notwendigkeit der "Übersetzung" zwischen den Systemen unterstreicht eine neue, wichtige Rolle der Leitlinie: die aktive Steuerung und Reduzierung potenzieller systemischer Verwirrung in der klinischen und wissenschaftlichen Gemeinschaft.

**Tabelle 1.1: Vergleichende Klassifikation der wichtigsten nodalen PTCLs**

| Entität/Konzept | WHO-HAEM4R (2017) | WHO-HAEM5 (2022) | International Consensus Classification (ICC) (2022) | Wichtige Änderungen/Implikationen |
| --- | --- | --- | --- | --- |
| **TFH-Lymphome** | Angioimmunoblastisches T-Zell-Lymphom (AITL) und andere nodale Lymphome TFH-Ursprungs (provisorisch) | **Familie:** Nodale T-Follikulär-Helferzell-Lymphome (nTFHL) mit 3 Subtypen: nTFHL, angioimmunoblastischer Typ; nTFHL, follikulärer Typ; nTFHL, NOS | **Eine Entität:** Follikuläres Helfer-T-Zell-Lymphom (TFHL) mit 3 Subtypen: angioimmunoblastischer, follikulärer und NOS-Subtyp | Vereinheitlichung basierend auf gemeinsamer Biologie (TFH-Phänotyp, *TET2/RHOA/IDH2*-Mutationen). Diagnostik erfordert Nachweis des TFH-Phänotyps. Direkte therapeutische Relevanz (s. 1.2). |
| **ALK-negatives ALCL** | Anaplastisch-großzelliges Lymphom, ALK-negativ | Anaplastisch-großzelliges Lymphom, ALK-negativ | Anaplastisch-großzelliges Lymphom, ALK-negativ | Anerkennung prognostisch relevanter genetischer Subtypen. Die ICC definiert ALCL mit *DUSP22*-Rearrangement als distinkten genetischen Subtyp. Direkte prognostische und therapeutische Relevanz (s. 1.3). |
| **PTCL-NOS** | Peripheres T-Zell-Lymphom, nicht weiter spezifiziert (NOS) | Peripheres T-Zell-Lymphom, NOS | Peripheres T-Zell-Lymphom, NOS | Bleibt eine Ausschlussdiagnose, aber molekulare Subtypen (GATA3, TBX21) werden als wünschenswerte, aber nicht essenzielle Klassifikatoren eingeführt. Legt die Grundlage für zukünftige zielgerichtete Studien (s. 1.4). |

**1.2 Neudefinition der T-Follikulär-Helferzell (TFH)-Lymphome: Vom AITL zu einer einheitlichen Klasse**

Kernaussage:

Die konzeptionelle Zusammenführung von AITL, follikulärem T-Zell-Lymphom und PTCL mit TFH-Phänotyp zu einer einzigen biologischen Gruppe (TFH-Lymphome) ist die bedeutendste Neuerung. Diese Reklassifizierung hat direkte therapeutische Konsequenzen, da der Nachweis eines TFH-Phänotyps als prädiktiver Biomarker für das Ansprechen auf epigenetisch wirksame Substanzen gilt.

**Details/Quellen:**

- **Studie/Quelle:** WHO-HAEM5 ^1^ und ICC-Publikationen.^2^
- **Patientenpopulation:** Patienten mit nodalen CD4-positiven PTCL.
- **Endpunkte:** Etablierung einer biologisch und klinisch kohärenten Krankheitsgruppe.
- **Rationale:** Die Vereinheitlichung basiert auf gemeinsamen molekularen Merkmalen, insbesondere den hochfrequenten Mutationen in Genen, die an der epigenetischen Regulation beteiligt sind (*TET2*, *DNMT3A*, *IDH2*), sowie einer spezifischen Mutation im *RHOA*-Gen (*G17V*).^1^ Diese molekulare Signatur ist in anderen PTCL-Subtypen selten und deutet auf eine gemeinsame Pathogenese hin.
- **Diagnostik:** Die Diagnose erfordert nun den immunhistochemischen Nachweis eines TFH-Phänotyps durch die Expression von mindestens zwei (besser drei) TFH-assoziierten Markern wie PD-1, ICOS, BCL6 oder CXCL13.^2^

Evidenzlevel/Empfehlungsgrad (geschätzt):

Evidenzlevel IIa, Empfehlungsgrad A.

Implikation für die Onkopedia-Leitlinie:

Diese Neuklassifizierung ist mehr als eine semantische Anpassung; sie stellt einen Paradigmenwechsel dar, der die Diagnostik direkt mit der Therapieentscheidung verknüpft. Zuvor wurde ein AITL primär anhand seines klassischen histologischen Bildes diagnostiziert. Ein PTCL-NOS ohne diese Morphologie, aber mit TFH-Marker-Expression, wäre möglicherweise mit Standard-CHOP behandelt worden. Die neuen Klassifikationen fordern, dass dieser Fall nun als TFH-Lymphom klassifiziert wird. Da klinische Daten, wie die Post-hoc-Analyse der Ro-CHOP-Studie oder frühe Daten zu Azacitidin, eine besondere Empfindlichkeit von TFH-Lymphomen gegenüber epigenetischen Therapien (HDAC-Inhibitoren, Hypomethylierungsagentien) nahelegen 11, wird die diagnostische Einordnung zu einem prädiktiven Akt. Die Onkopedia-Leitlinie muss daher die Testung auf TFH-Marker (mindestens PD-1 und ICOS) von "nützlich" auf "essenziell" für die Abklärung aller nodalen CD4-positiven PTCL hochstufen. Das Ergebnis dieser Testung informiert direkt über Therapieoptionen im Rezidiv und die Rationale für die Teilnahme an entsprechenden Erstlinientherapiestudien.

**1.3 Anaplastisch-großzelliges Lymphom (ALCL): Integration genetischer Subtypen**

Kernaussage:

Die Entität des ALK-negativen ALCL ist nicht länger monolithisch. Die Identifizierung von genetischen Subtypen, insbesondere des DUSP22-Rearrangements mit guter Prognose und des TP63-Rearrangements mit sehr schlechter Prognose, ermöglicht erstmals eine molekulare Risikostratifizierung, die Therapieentscheidungen, insbesondere bezüglich der Konsolidierung, beeinflussen sollte.

**Details/Quellen:**

- **Studie/Quelle:** WHO-HAEM5 ^1^ und ICC-Publikationen.^5^
- **Patientenpopulation:** Patienten mit systemischem ALK-negativem ALCL.
- **Genetische Marker:**
- **DUSP22-Rearrangement (DUSP22-r):** Findet sich bei ca. 30% der ALK-negativen ALCL. Diese Fälle haben eine deutlich bessere Prognose, die oft der des ALK-positiven ALCL nahekommt.^9^ Die ICC erkennt dies als eigenen genetischen Subtyp an.^9^
- **TP63-Rearrangement (TP63-r):** Seltener (ca. 5%), aber mit einer sehr aggressiven Klinik und infauster Prognose assoziiert.^9^
- **Leitlinien-Update:** Die NCCN-Leitlinie (Version 2.2022) merkt an, dass für ALK-negative ALCL mit *DUSP22*-Rearrangement eine Therapie analog zum ALK-positiven ALCL erwogen werden kann.^15^

Evidenzlevel/Empfehlungsgrad (geschätzt):

Evidenzlevel IIb, Empfehlungsgrad B.

Implikation für die Onkopedia-Leitlinie:

Die historische Einteilung des ALCL in nur ALK-positiv und ALK-negativ ist überholt. Die molekularen Befunde haben direkte klinische Konsequenzen. Ein Patient mit einem DUSP22-rearrangierten ALCL hat eine so gute Prognose, dass die Notwendigkeit einer konsolidierenden autologen Stammzelltransplantation in der Erstremission in Frage gestellt werden muss, ähnlich wie es beim ALK-positiven ALCL der Fall ist. Umgekehrt haben Patienten mit einem TP63-Rearrangement eine derart schlechte Prognose, dass eine Standard-Chemotherapie als unzureichend angesehen werden muss; diese Patienten sollten nach Möglichkeit umgehend einer allogenen Stammzelltransplantation oder experimentellen Therapien zugeführt werden. Die Onkopedia-Leitlinie sollte daher die FISH-Analyse auf DUSP22- und TP63-Rearrangements als Standarddiagnostik bei allen neu diagnostizierten ALK-negativen ALCL empfehlen. Die prognostische Stratifizierung muss entsprechend angepasst werden in: 1. Günstige Prognose (ALK-positiv und DUSP22-rearrangiert), 2. Intermediäre Prognose ("Triple-negativ"), und 3. Sehr ungünstige Prognose (TP63-rearrangiert).

**1.4 Molekulare Subklassifikation des PTCL-NOS**

Kernaussage:

Obwohl noch nicht für die klinische Routine etabliert, ermöglicht die molekulare Subklassifikation des PTCL-NOS in GATA3- und TBX21-Subtypen eine biologisch fundierte Aufteilung dieser heterogenen "Restekategorie". Diese Entwicklung ist entscheidend für das Design zukünftiger, zielgerichteter klinischer Studien.

**Details/Quellen:**

- **Studie/Quelle:** Genexpressionsstudien, z.B. zitiert in den neuen Klassifikationen.^2^
- **Patientenpopulation:** Patienten mit PTCL-NOS.
- **Molekulare Subtypen:**
- **PTCL-GATA3:** Assoziiert mit einem Th2-Zell-Profil, hoher genomischer Komplexität (z.B. *TP53*-Alterationen), Aktivierung des PI3K-Signalwegs und einer schlechteren Prognose.^2^
- **PTCL-TBX21:** Assoziiert mit einem Th1/zytotoxischen Profil, geringerer genomischer Komplexität, Aktivierung des JAK/STAT-Signalwegs und einer besseren Prognose.^2^
- **Diagnostik:** Die Einteilung erfolgt primär über Genexpressionsanalysen. Ein immunhistochemischer Surrogat-Algorithmus wurde vorgeschlagen, gilt aber noch nicht als validierter Standard für die Routinediagnostik.^2^

Evidenzlevel/Empfehlungsgrad (geschätzt):

Evidenzlevel III, Empfehlungsgrad C.

Implikation für die Onkopedia-Leitlinie:

Es ist verfrüht, eine routinemäßige molekulare Subklassifikation des PTCL-NOS zur Therapieentscheidung zu empfehlen. Dennoch ist es für eine zukunftsorientierte Leitlinie unerlässlich, diesen Fortschritt detailliert darzustellen. Die Leitlinie sollte erläutern, dass die Heterogenität des PTCL-NOS eine Hauptursache für das Scheitern vieler klinischer Studien war. Die GATA3/TBX21-Einteilung bietet erstmals eine biologische Rationale für das Design von zielgerichteten Studien, z.B. mit PI3K-Inhibitoren für den GATA3-Subtyp oder JAK-Inhibitoren für den TBX21-Subtyp. Die Leitlinie sollte daher festhalten, dass CHO(E)P der Standard bleibt, aber die Teilnahme an klinischen Studien, die nach diesen molekularen Subtypen stratifizieren oder für diese selektionieren, dringend empfohlen wird. Pathologen und Kliniker sollten für diese Entitäten sensibilisiert werden.

**1.5 CD30-Immunhistochemie: Jenseits einer starren Schwelle**

Kernaussage:

Die klinische Praxis und neue Expertenempfehlungen weichen von einem starren CD30-Schwellenwert von ≥10% für den Einsatz von Brentuximab Vedotin ab. Die CD30-Expression sollte als Kontinuum verstanden und im pathologischen Befund quantitativ (Prozentsatz und Intensität) beschrieben werden, um eine fundierte klinische Einzelfallentscheidung zu ermöglichen.

**Details/Quellen:**

- **Studie/Quelle:** ECHELON-2-Studiendesign ^17^, Phase-II-Studien und Expertenkonsensus-Publikationen.^18^
- **Patientenpopulation:** Patienten mit PTCL, die für eine CD30-gerichtete Therapie in Frage kommen.
- **Endpunkte/Beobachtungen:**
- Die ECHELON-2-Studie verwendete einen Cut-off von ≥10% CD30-positiven Tumorzellen als Einschlusskriterium.^17^
- Klinische Studien und Erfahrungen zeigen jedoch ein Ansprechen auf Brentuximab Vedotin (BV) auch bei Patienten mit einer CD30-Expression von <10%.^18^
- Dieser Effekt wird u.a. auf einen "Bystander-Effekt" zurückgeführt, bei dem das von der Zielzelle freigesetzte Toxin auch benachbarte, CD30-negative Tumorzellen abtötet.^18^
- Eine Experten-Panel-Empfehlung von 2022 rät, jeglichen Grad der CD30-Expression zu berichten, inklusive des geschätzten Prozentsatzes positiver Tumorzellen und der Färbeintensität.^19^

Evidenzlevel/Empfehlungsgrad (geschätzt):

Evidenzlevel IIb, Empfehlungsgrad B.

Implikation für die Onkopedia-Leitlinie:

Die Leitlinie muss die bisherige, oft rigide Interpretation des 10%-Schwellenwerts aufweichen. Es sollte klargestellt werden, dass dieser Wert aus dem Design der Zulassungsstudie stammt und keine absolute biologische Grenze für die Wirksamkeit darstellt. Die Empfehlung für die pathologische Diagnostik sollte aktualisiert werden, um den Expertenempfehlungen von 2022 zu folgen: Der Befund sollte nicht nur "positiv" oder "negativ" lauten, sondern den Prozentsatz der positiven Tumorzellen und die Intensität (schwach, moderat, stark) quantifizieren. Dies gibt dem behandelnden Kliniker die notwendige Information, um auch bei Patienten mit niedriger CD30-Expression eine fundierte Entscheidung für oder gegen eine Therapie mit BV-CHP zu treffen, insbesondere im Kontext des histologischen Subtyps und der klinischen Gesamtsituation.

**Sektion 2: Evolution der Erstlinientherapie bei PTCL**

Die Entwicklungen in der Erstlinientherapie seit 2021 haben die Behandlungsstandards für einen Teil der PTCL-Patienten zementiert, während sie für andere die Dringlichkeit für neue Ansätze unterstreichen. Die Langzeitdaten der ECHELON-2-Studie haben die Therapie der CD30-positiven PTCL revolutioniert. Für CD30-negative Erkrankungen bleibt die Suche nach einer Verbesserung gegenüber dem CHOP-Standard eine zentrale Herausforderung, die durch innovative, randomisierte Studien adressiert wird. Parallel dazu schärft sich das Bild zur Rolle der konsolidierenden Stammzelltransplantation durch neue, subtyp-spezifische Analysen.

**2.1 CD30-positive PTCL: Der nachhaltige Gesamtüberlebensvorteil von BV-CHP**

Kernaussage:

Das 5-Jahres-Follow-up der ECHELON-2-Studie bestätigt einen anhaltenden und signifikanten Vorteil im Gesamtüberleben für Brentuximab Vedotin + CHP (BV-CHP) gegenüber CHOP. Dies etabliert BV-CHP endgültig als Standard-Erstlinientherapie für alle neu diagnostizierten, systemischen CD30-positiven PTCL, insbesondere für das sALCL.

**Studiendetails/Quelle:**

- **Studie:** ECHELON-2 (5-Jahres-Update)
- **Publikation/Präsentation:** Horwitz S. et al., Annals of Oncology, 2022.^17^
- **Patientenpopulation:** n=452, Patienten mit neu diagnostiziertem CD30-positivem PTCL (CD30-Expression ≥10%), ca. 70% mit systemischem anaplastisch-großzelligem Lymphom (sALCL).
- **Endpunkte:**
- **5-Jahres Progressionsfreies Überleben (PFS):** 51.4% (BV-CHP) vs. 43.0% (CHOP); Hazard Ratio (HR) 0.70 (95% CI: 0.53-0.91), p=0.0077.^22^
- **5-Jahres Gesamtüberleben (OS):** 70.1% (BV-CHP) vs. 61.0% (CHOP); HR 0.72 (95% CI: 0.53-0.99), p=0.0424.^22^
- **sALCL-Subgruppe:** Der Vorteil war im sALCL-Kollektiv besonders ausgeprägt mit einer 5-Jahres-PFS-Rate von 60.6% vs. 48.4% (HR 0.55).^22^
- **Sicherheit:** Das Sicherheitsprofil blieb beherrschbar. Insbesondere die periphere Neuropathie (PN) besserte sich oder sistierte bei 72% der Patienten im BV-CHP-Arm.^21^

Evidenzlevel/Empfehlungsgrad (geschätzt):

Evidenzlevel Ia, Empfehlungsgrad A.

Implikation für die Onkopedia-Leitlinie:

Die Bestätigung eines signifikanten Gesamtüberlebensvorteils nach 5 Jahren ist ein seltenes und äußerst starkes Signal in der PTCL-Therapielandschaft. Dies festigt die Position von BV-CHP als unangefochtenen Therapiestandard. Die Leitlinie muss die Empfehlung entsprechend schärfen:

1. Für alle neu diagnostizierten, transplantationsfähigen Patienten mit systemischem ALCL (sowohl ALK-positiv als auch ALK-negativ) ist BV-CHP als Standardtherapie mit dem höchsten Empfehlungsgrad (A) zu deklarieren.
2. Für andere CD30-positive PTCL-Subtypen (z.B. PTCL-NOS, AITL mit CD30-Expression ≥10%) sollte BV-CHP als die klar bevorzugte Therapieoption gegenüber CHO(E)P formuliert werden. Die Empfehlung stützt sich auf den positiven Ausgang der Gesamtstudie, auch wenn die Evidenz für die Nicht-ALCL-Subgruppen aufgrund kleinerer Fallzahlen weniger robust ist. Die Extrapolation ist jedoch angesichts des OS-Vorteils gerechtfertigt und wird von internationalen Leitlinien wie dem NCCN getragen.

**2.2 CD30-negative/niedrige PTCL: Die Suche nach einem Nachfolger für CHO(E)P**

Kernaussage:

Für CD30-negative PTCL konnte seit 2021 kein neuer Therapiestandard etabliert werden, der über CHO(E)P hinausgeht. Die aktuelle Forschungsstrategie konzentriert sich auf randomisierte Phase-II-Studien, die die Addition von zielgerichteten Substanzen zum CHOP-Backbone prüfen, basierend auf der spezifischen Biologie der PTCL-Subtypen.

**Studiendetails/Quelle:**

- **Studie:** Alliance A051902 (randomisierte Phase II).^23^
- **Publikation/Präsentation:** Studiendesign publiziert, Rekrutierung seit Juli 2021, Ergebnisse ausstehend.
- **Patientenpopulation:** Patienten mit neu diagnostiziertem, CD30-negativem PTCL.
- **Studiendesign:** Randomisierung in drei Arme: 1) CHO(E)P (Standard), 2) CHO(E)P + orales Azacitidin (CC-486), 3) CHO(E)P + Duvelisib (PI3K-Inhibitor).
- **Rationale:** Dieser Studienansatz verkörpert den strategischen Wandel weg von "One-size-fits-all"-Studien. Die Hinzunahme von Azacitidin zielt spezifisch auf die TFH-Lymphome (wie AITL), die häufig epigenetische Mutationen aufweisen. Duvelisib zielt auf Subtypen mit Aktivierung des PI3K-Signalwegs, wie sie im PTCL-GATA3-Subtyp beschrieben sind.^11^
- **Weitere Evidenz:** Die finale Analyse der Ro-CHOP-Studie (Romidepsin + CHOP), publiziert 2024, war im Gesamtkollektiv negativ. Eine explorative Subgruppenanalyse deutete jedoch auf einen PFS-Vorteil für Patienten mit TFH-Phänotyp hin ^13^, was die Rationale für biologisch stratifizierte Studien retrospektiv untermauert.

Evidenzlevel/Empfehlungsgrad (geschätzt):

Evidenzlevel III (für neue Kombinationen), Empfehlungsgrad B (für Studienteilnahme).

Implikation für die Onkopedia-Leitlinie:

Die Leitlinie muss konstatieren, dass für CD30-negative/niedrige PTCL weiterhin CHO(E)P der Standard außerhalb von Studien ist. Die wichtigste und praxisverändernde Empfehlung ist jedoch, die Teilnahme an klinischen Studien als bevorzugte Option für alle geeigneten Patienten hervorzuheben. Es ist entscheidend, nicht nur die Existenz von Studien zu erwähnen, sondern auch die wissenschaftliche Rationale hinter dem modernen Studiendesign zu erläutern. Die Leitlinie sollte das Konzept der "Add-on"-Strategie und die Bedeutung der biologischen Stratifizierung (z.B. TFH-Phänotyp) erklären. Dies schult die Anwender der Leitlinie darin, warum die Teilnahme an Studien wie Alliance A051902 nicht nur eine Option, sondern der wissenschaftlich fundierteste Ansatz ist, um die Therapie für diese Patientengruppe zu verbessern.

**2.3 Die Rolle der upfront-Konsolidierung mit Stammzelltransplantation (SZT)**

Kernaussage:

Neue Daten aus großen Kohortenstudien stärken die Evidenz für eine subtyp-spezifische Indikationsstellung zur autologen Stammzelltransplantation (ASZT) in der Erstremission (CR1). Insbesondere für Patienten mit AITL zeigt sich konsistent ein Überlebensvorteil, während die Rolle bei PTCL-NOS unklar und bei sALCL nach BV-CHP-Therapie fraglich bleibt.

**Studiendetails/Quelle:**

- **Studien:** Mehrere große, teils prospektive, teils retrospektive Kohortenstudien wurden seit 2021 publiziert.
- **Cabrera et al., *Haematologica*, 2022 (retrospektiv, international):** Bei 174 Patienten in CR1 war die ASZT mit einem signifikant besseren 5-Jahres-PFS (63% vs. 48%) und OS (insbesondere bei fortgeschrittenen Stadien) assoziiert.^25^
- **Mehta-Shah et al., *Cancer*, 2022 (prospektive COMPLETE-Kohorte):** Die ASZT war in der Multivarianzanalyse unabhängig mit einem verbesserten Überleben assoziiert (HR 0.37). Der Nutzen war signifikant für Patienten mit AITL, jedoch nicht für andere Subtypen.^26^
- **Yoon et al., *Cancer Res Treat*, 2023 (prospektives koreanisches Register):** Die ASZT verbesserte das PFS bei Patienten mit AITL signifikant, nicht aber bei Patienten mit PTCL-NOS.^27^
- **Patientenpopulation:** Transplantationsfähige Patienten mit PTCL in CR1.
- **Endpunkte:** PFS und OS, verglichen zwischen Patienten, die eine ASZT erhielten, und solchen, die keine erhielten.

Evidenzlevel/Empfehlungsgrad (geschätzt):

Evidenzlevel IIb, Empfehlungsgrad B (subtyp-spezifisch).

Implikation für die Onkopedia-Leitlinie:

Die Empfehlung zur konsolidierenden ASZT muss von einer pauschalen Empfehlung für "Hochrisiko-PTCL" zu einer differenzierten, subtyp-basierten Empfehlung weiterentwickelt werden. Die Datenlage verdichtet sich dahingehend, dass der Nutzen der ASZT nicht für alle PTCL gleich ist.

1. **AITL:** Für transplantationsfähige Patienten mit AITL in CR1 sollte die konsolidierende ASZT als Standard empfohlen werden (starke Empfehlung). Mehrere unabhängige Studien belegen hier einen klaren Vorteil.^26^
2. **PTCL-NOS:** Die Datenlage ist weiterhin uneinheitlich. Die ASZT kann als Option erwogen werden, die Evidenz ist jedoch schwächer als beim AITL.^27^ Die Entscheidung sollte individualisiert getroffen werden.
3. **sALCL:** Für Patienten mit sALCL, die mit BV-CHP eine CR erreichen, ist der Stellenwert der ASZT unklar und sollte nicht als routinemäßiger Standard empfohlen werden. Die bereits hohe 5-Jahres-PFS-Rate von ca. 60% mit BV-CHP allein stellt den zusätzlichen Nutzen einer intensiven Konsolidierung in Frage.^22^
4. **Allo-SZT:** Die allogene SZT in CR1 bleibt eine Option für ultra-Hochrisiko-Entitäten wie das hepatosplenische T-Zell-Lymphom (HSTL).^28^

**Sektion 3: Paradigmenwechsel in der Therapie des rezidivierten/refraktären (R/R) PTCL**

Im Bereich der rezidivierten/refraktären (R/R) PTCL war der Zeitraum seit Juni 2021 weniger von neuen Zulassungen geprägt, sondern vielmehr von einer Neubewertung etablierter Substanzen und dem Aufkommen hochinnovativer Therapieansätze. Ein zentrales Ereignis war die Rücknahme der Zulassung für Romidepsin, was die gestiegenen Anforderungen an die Evidenz für zielgerichtete Therapien unterstreicht. Gleichzeitig liefern klinische Studien neue Daten zu Kombinationstherapien und die ersten vielversprechenden Ergebnisse zu zellulären Therapien deuten auf einen bevorstehenden Wandel in der Behandlungssequenz hin.

**3.1 Das regulatorische Umfeld: Eine Phase der Rekalibrierung**

Kernaussage:

Seit Juni 2021 gab es keine neuen breiten Zulassungen von Medikamenten für R/R PTCL durch EMA oder FDA. Prägend war die Rücknahme der PTCL-Indikation für Romidepsin in den USA, was den erhöhten Druck der Behörden widerspiegelt, auf beschleunigten Zulassungen basierende Indikationen durch randomisierte Studien zu bestätigen.

**Details/Quellen:**

- **Neue Zulassungen:** Keine neuen Substanzen wurden für die breite Indikation R/R PTCL zugelassen.^30^
- **Zulassungsrücknahme:** Im Jahr 2021 wurde die Indikation für den HDAC-Inhibitor Romidepsin zur Behandlung von R/R PTCL in den USA freiwillig vom Hersteller zurückgezogen. Dies geschah, nachdem die konfirmatorische Phase-III-Studie (Ro-CHOP) in der Erstlinie ihren primären Endpunkt nicht erreichte und somit der klinische Nutzen nicht ausreichend belegt werden konnte, um die zuvor erteilte beschleunigte Zulassung aufrechtzuerhalten.^45^ Die Zulassung für das kutane T-Zell-Lymphom (CTCL) bleibt bestehen.^45^
- **Positive Signale:** Der ITK-Inhibitor Soquelitinib erhielt im August 2024 von der FDA den "Fast Track Designation"-Status für R/R PTCL, was auf ein hohes Interesse der Behörde an dieser neuen Substanzklasse hindeutet und den Entwicklungsprozess beschleunigen könnte.^47^

Evidenzlevel/Empfehlungsgrad (geschätzt):

N/A (regulatorische Information).

Implikation für die Onkopedia-Leitlinie:

Die Rücknahme der Romidepsin-Zulassung ist ein wichtiges Signal. In den 2010er Jahren erhielten mehrere Medikamente für R/R PTCL eine beschleunigte Zulassung auf Basis von einarmigen Phase-II-Studien mit moderaten Ansprechraten. Die regulatorischen Hürden für eine Bestätigung dieser Zulassungen sind nun erkennbar höher. Die Leitlinie muss den Status von Romidepsin aktualisieren und darauf hinweisen, dass die Evidenzbasis für seinen Einsatz bei nodalen PTCL schwach ist. Generell sollte ein Hinweis ergänzt werden, dass Therapien, die nur auf Basis einarmiger Studien zugelassen sind, kritisch bewertet werden müssen und ihre Position im Therapiealgorithmus sich ändern kann. Dies schärft das Bewusstsein der Anwender für die unterschiedliche Qualität der Evidenz hinter den verfügbaren Therapieoptionen.

**3.2 Gezielte Therapien und Kombinationen: Eine evidenzbasierte Neubewertung**

**3.2.1 Hypomethylierende Substanzen (HMAs) bei AITL: Die ORACLE-Studie**

Kernaussage:

Die randomisierte Phase-III-Studie ORACLE erreichte ihren primären Endpunkt einer PFS-Verbesserung für orales Azacitidin gegenüber der Standardtherapie bei R/R AITL/TFH-Lymphomen nicht. Ein beobachteter Trend zu einem längeren Gesamtüberleben bei gleichzeitig besserer Verträglichkeit positioniert orales Azacitidin jedoch als eine relevante Therapieoption für ausgewählte Patienten.

**Studiendetails/Quelle:**

- **Studie:** ORACLE (NCT03593018), Phase III, randomisiert.
- **Publikation/Präsentation:** Lemonnier F. et al., ASH 2022.^48^
- **Patientenpopulation:** n=86, Patienten mit R/R AITL oder nodalem TFH-Lymphom.
- **Endpunkte:**
- **Primärer Endpunkt (PFS):** Median 5.6 Monate (orales Azacitidin) vs. 4.8 Monate (Investigator's Choice: Gemcitabin, Bendamustin, Romidepsin); HR 0.73, p=0.13.^48^ Der Endpunkt wurde nicht erreicht.
- **Sekundärer Endpunkt (OS):** Median 18.4 Monate vs. 10.3 Monate; HR 0.557 (95% CI: 0.323-0.961).^48^
- **Sicherheit:** Weniger Grad ≥3 unerwünschte Ereignisse im Azacitidin-Arm (76.2% vs. 97.7%), insbesondere weniger hämatologische Toxizität.^48^

Evidenzlevel/Empfehlungsgrad (geschätzt):

Evidenzlevel Ib (für PFS negativ), IIb (für OS-Signal und Sicherheit), Empfehlungsgrad B (als Option).

Implikation für die Onkopedia-Leitlinie:

Die Interpretation einer Studie mit negativem primären, aber positivem sekundären Endpunkt ist komplex, insbesondere bei seltenen Erkrankungen, wo Studien oft unterpowert sind. Ein strikt evidenzbasierter Ansatz würde eine Empfehlung ausschließen. Jedoch ist ein um 8 Monate verlängertes medianes Gesamtüberleben bei besserer Verträglichkeit klinisch hochrelevant. Die Leitlinie sollte die Studienergebnisse differenziert darstellen: Orales Azacitidin kann nicht als neuer Standard empfohlen werden. Es sollte aber als eine wichtige Therapieoption für Patienten mit R/R AITL/TFH-Lymphom aufgeführt werden, insbesondere für solche, bei denen die Toxizitätsvermeidung im Vordergrund steht (z.B. ältere oder komorbide Patienten). Der OS-Vorteil sollte als Signal gewertet werden, das den Einsatz im Einzelfall rechtfertigen kann.

**3.2.2 PI3K-Inhibitoren: Neubewertung von Duvelisib**

Kernaussage:

Duvelisib zeigt weiterhin eine relevante Wirksamkeit bei R/R PTCL, insbesondere bei AITL. Die hohe Rate an immunvermittelten, teils schweren Nebenwirkungen schränkt seinen Einsatz jedoch erheblich ein und erfordert ein erfahrenes Management. Sein Stellenwert liegt zunehmend in der kurzfristigen Anwendung als Brücke zu einer konsolidierenden Therapie.

**Studiendetails/Quelle:**

- **Studie:** Meta-Analyse und Kombinationsstudien.
- **Publikation/Präsentation:** Zhang M. et al., Frontiers in Immunology, 2022 (Meta-Analyse) ^49^; Horwitz S. et al., Blood, 2024 (Kombination mit Romidepsin).^50^
- **Wirksamkeit:** Die Meta-Analyse bestätigte eine gepoolte Gesamtansprechrate (ORR) von 47% bei T-NHL, mit höheren Raten bei AITL (67%) im Vergleich zu anderen PTCL (42%).^49^
- **Toxizität:** Hohe Raten an Grad ≥3 Nebenwirkungen (79%), insbesondere Diarrhoe, Transaminasenanstieg und Neutropenie, sowie eine hohe Rate an Therapieabbrüchen (33%) wurden bestätigt.^49^
- **Kombinationen:** Eine Phase-1b/2a-Studie, die Duvelisib mit Romidepsin kombinierte, zeigte eine hohe Wirksamkeit (ORR 56% bei PTCL) bei scheinbar geringerer Hepatotoxizität im Vergleich zur Duvelisib-Monotherapie, was auf eine mögliche Milderung der Toxizität durch die Kombination hindeutet.^50^

Evidenzlevel/Empfehlungsgrad (geschätzt):

Evidenzlevel IIa, Empfehlungsgrad C (aufgrund der Toxizität).

Implikation für die Onkopedia-Leitlinie:

Das therapeutische Fenster für Duvelisib ist schmal. Die Leitlinie muss eine deutliche Warnung bezüglich des Toxizitätsprofils aussprechen. Die Anwendung sollte auf erfahrene Zentren beschränkt sein, die ein proaktives Management der immunvermittelten Nebenwirkungen gewährleisten können. Aufgrund der hohen Rate an Therapieabbrüchen ist eine Langzeittherapie oft nicht möglich. Daher sollte die primäre Rationale für den Einsatz von Duvelisib als eine effektive "Brückentherapie" zur Erreichung einer Remission vor einer geplanten allogenen Stammzelltransplantation formuliert werden. Die vielversprechenden Daten zur Kombination mit Romidepsin sollten als experimenteller Ansatz erwähnt werden.

**3.3 Die neue Grenze der zellulären Immuntherapie: CD5- und CD7-gerichtete CAR-T-Zellen**

Kernaussage:

Frühe klinische Daten zu CD5- und CD7-gerichteten CAR-T-Zell-Therapien zeigen beeindruckende Ansprechraten bei schwer vorbehandelten Patienten mit T-Zell-Malignomen, einschließlich PTCL. Obwohl noch experimentell, etablieren sich diese Therapien als potenziell wirksamste Salvage-Strategie und als eine neue, hochpotente Brücke zur allogenen Stammzelltransplantation.

**Studiendetails/Quelle:**

- **Studien:** Mehrere Phase-I-Studien, präsentiert u.a. auf ASH 2023 und 2024.
- **CD7 CAR-T:**
- **Zielstruktur:** CD7 wird auf den meisten T-ALL/LBL und einem Teil der PTCL exprimiert.^51^
- **Herausforderung:** "Fratrizid" (gegenseitiges Abtöten der CAR-T-Zellen) und T-Zell-Aplasie.^52^ Lösungen umfassen Gen-Editing (z.B. CRISPR/Cas9-Knockout von
  *CD7*) oder Protein-Blocker.^51^
- **Ergebnisse:** Hohe CR-Raten bei T-ALL/LBL (bis zu 91%).^54^ Eine erste Studie bei 5 R/R PTCL-Patienten (u.a. PTCL-NOS, HSTL, ENKTL) zeigte eine ORR von 80% (3 CR, 1 PR).^55^ Eine Konsolidierung mittels allo-SZT scheint für die Dauerhaftigkeit des Ansprechens entscheidend zu sein.^51^
- **CD5 CAR-T:**
- **Zielstruktur:** CD5 ist ein Pan-T-Zell-Marker.
- **Herausforderung:** Ein innovativer Ansatz nutzt einen CAR-Konstrukt, der zu einer Downregulation des CD5-Oberflächenproteins auf den CAR-T-Zellen selbst führt und so das Fratrizid ohne Gen-Editing verhindert.^57^
- **Ergebnisse:** Eine Phase-I-Studie bei 9 Patienten mit R/R TCL (u.a. AITL, PTCL) zeigte eine ORR von 44% (2 CRs). Drei der vier ansprechenden Patienten konnten erfolgreich einer allo-SZT zugeführt werden. Das Sicherheitsprofil war sehr günstig, ohne hochgradiges Zytokinfreisetzungssyndrom (CRS) oder Neurotoxizität.^57^

Evidenzlevel/Empfehlungsgrad (geschätzt):

Evidenzlevel III, Empfehlungsgrad C (als experimentelle Therapie).

Implikation für die Onkopedia-Leitlinie:

CAR-T-Zell-Therapien für T-Zell-Lymphome sind dabei, die Behandlung von ansonsten therapieresistenten Patienten zu revolutionieren. Die Leitlinie muss einen neuen Abschnitt zu "Experimentellen zellulären Therapien" aufnehmen. Die vielversprechenden, aber frühen Daten für CD5- und CD7-CAR-T-Zellen sollten zusammengefasst werden. Es muss klar kommuniziert werden, dass dies derzeit keine Standardtherapie ist. Die entscheidende Implikation für die klinische Praxis ist die Neudefinition der Behandlungssequenz: Für Patienten mit R/R PTCL, die für eine allo-SZT in Frage kommen, aber keine ausreichende Remission erreichen können, stellt die Teilnahme an einer CAR-T-Zell-Studie die vielversprechendste Option dar, um eine tiefe Remission als "Brücke zur Transplantation" zu erzielen. Dies ist eine vorausschauende, praxisinformierende Empfehlung, die Kliniker auf den nächsten Paradigmenwechsel vorbereitet.

**Tabelle 3.1: Wichtige Phase-II/III-Studien mit neuen Substanzen bei R/R PTCL (berichtet seit Juni 2021)**

| Studie/ID | Medikament(e) | Mechanismus | Patientenpopulation (Subtyp) | N | Wichtige Wirksamkeitsergebnisse (ORR, CR, mediane PFS/OS) | Wichtige Grad ≥3 Toxizitäten | Implikation/Kommentar |
| --- | --- | --- | --- | --- | --- | --- | --- |
| **ORACLE** (NCT03593018) | Orales Azacitidin | Hypomethylierende Substanz | R/R AITL / TFH-Lymphom | 86 | ORR: 33%, CR: 12%. Med. PFS: 5.6 Monate (vs. 4.8). Med. OS: 18.4 Monate (vs. 10.3) ^48^ | Neutropenie (43%), Thrombozytopenie (24%). Geringere Toxizität als Standardtherapie ^48^ | Primärer Endpunkt (PFS) nicht erreicht, aber OS-Signal und günstiges Sicherheitsprofil. Option für ausgewählte AITL-Patienten. |
| **Duvelisib + Romidepsin** (NCT02783625) | Duvelisib + Romidepsin | PI3K-δ/γ-Inhibitor + HDAC-Inhibitor | R/R TCL (n=48 PTCL) | 66 | PTCL: ORR 56%, CR 44%. TFH-Subtyp mit besonders hohem Ansprechen ^50^ | Neutropenie (42%), Thrombozytopenie, Fatigue. Weniger Hepatotoxizität als Duvelisib-Monotherapie ^50^ | Hochwirksame Kombination, insbesondere bei TFH-Lymphomen. Vielversprechender Ansatz, der im Rahmen von Studien weiterverfolgt werden sollte. |
| **CD7 CAR-T** (NCT04928105) | NS7CAR-T | Zelluläre Immuntherapie | R/R PTCL (diverse Subtypen) | 5 | ORR 80% (3 CR, 1 PR). 3/4 Responder wurden zur allo-SZT überführt ^55^ | CRS Grad 1-2 (100%), keine Neurotoxizität. Hämatotoxizität ^55^ | Sehr hohe Ansprechraten in einer stark vorbehandelten Population. Etabliert CAR-T als potente Brücke zur Transplantation. |
| **CD5 CAR-T** (NCT0308190) | Autologe CD5 CAR-T | Zelluläre Immuntherapie | R/R TCL (diverse Subtypen) | 9 | ORR 44% (2 CR). 3/4 Responder wurden zur allo-SZT überführt ^57^ | Keine Grad ≥3 CRS/ICANS. Hämatotoxizität ^57^ | Günstiges Sicherheitsprofil und klinische Wirksamkeit. Innovativer Ansatz zur Vermeidung von Fratrizid. |

**Sektion 4: Aktualisierte Empfehlungen für spezifische und seltene PTCL-Subtypen**

Die allgemeinen Fortschritte in Diagnostik und Therapie müssen in subtyp-spezifische Empfehlungen übersetzt werden, um der Heterogenität der PTCL gerecht zu werden. Die folgenden Abschnitte fassen die praxisverändernden Entwicklungen für die klinisch relevantesten Entitäten zusammen.

**4.1 Angioimmunoblastisches T-Zell-Lymphom (AITL)**

Kernaussage:

Die Behandlung des AITL sollte sich auf zwei Säulen stützen: eine epigenetisch zielgerichtete Therapie im R/R-Setting und die konsolidierende autologe Stammzelltransplantation in der Erstremission.

Synthese der Evidenz:

Als Prototyp des nun als TFH-Lymphom klassifizierten Spektrums ist das AITL durch eine hohe Frequenz an Mutationen in epigenetischen Regulatorgenen (TET2, IDH2, DNMT3A) gekennzeichnet.1 Diese biologische Eigenschaft macht es zu einem primären Ziel für epigenetisch wirksame Medikamente. Die Phase-III-Studie ORACLE, obwohl im primären Endpunkt negativ, zeigte einen klinisch relevanten Trend zu einem verlängerten Gesamtüberleben mit oralem Azacitidin bei guter Verträglichkeit, was diese Substanz zu einer wichtigen Option im R/R-Setting macht.^48^ Darüber hinaus haben Kombinationsstudien mit HDAC-Inhibitoren (Romidepsin) und entweder HMAs (Azacitidin) oder PI3K-Inhibitoren (Duvelisib) besonders hohe Ansprechraten bei TFH-Lymphomen, einschließlich AITL, gezeigt.^50^ Für die Erstlinientherapie hat sich die Datenlage zur konsolidierenden ASZT in CR1 deutlich verdichtet. Mehrere große, unabhängige Kohortenstudien, die seit 2021 publiziert wurden, zeigen konsistent einen signifikanten Überlebensvorteil (sowohl PFS als auch OS) für Patienten mit AITL, die in CR1 transplantiert werden.^25^

Implikation für die Onkopedia-Leitlinie:

Die Empfehlungen für AITL müssen umfassend überarbeitet werden.

- **Erstlinientherapie:** Die Empfehlung für eine konsolidierende ASZT nach Erreichen einer CR1 mit einer CHO(E)P-basierten Induktion sollte von einer Option zu einer starken Empfehlung (Empfehlungsgrad B) hochgestuft werden.
- **Therapie des R/R AITL:** Epigenetisch zielgerichtete Therapien sollten als bevorzugte Optionen hervorgehoben werden. Orales Azacitidin sollte als gut verträgliche Option mit einem OS-Signal aufgeführt werden. Die hochwirksamen Kombinationen (z.B. Romidepsin + Azacitidin) sollten als vielversprechende Ansätze erwähnt werden, deren Einsatz vorzugsweise im Rahmen von Studien oder an erfahrenen Zentren erfolgen sollte.

**4.2 ALK-negatives Anaplastisch-großzelliges Lymphom (ALK- ALCL)**

Kernaussage:

BV-CHP ist der unbestrittene Erstlinientherapie-Standard. Die neue molekulare Risikostratifizierung anhand von DUSP22- und TP63-Rearrangements sollte die Indikationsstellung zur Konsolidierungstherapie leiten.

Synthese der Evidenz:

Die 5-Jahres-Daten der ECHELON-2-Studie liefern Evidenz des Levels Ia für die Überlegenheit von BV-CHP gegenüber CHOP in der Erstlinientherapie, mit einem signifikanten Vorteil im Gesamtüberleben.^17^ Dies bestätigt BV-CHP als Standardtherapie. Parallel dazu hat die molekulare Charakterisierung des ALK-negativen ALCL zur Identifizierung prognostisch hochrelevanter Subgruppen geführt. Das Vorhandensein eines

*DUSP22*-Rearrangements ist mit einer exzellenten Prognose assoziiert, während ein *TP63*-Rearrangement eine infauste Prognose anzeigt.^4^

**Implikation für die Onkopedia-Leitlinie:**

- **Erstlinientherapie:** BV-CHP muss als Standardtherapie mit dem höchsten Empfehlungsgrad (A) für alle Patienten mit neu diagnostiziertem ALK-negativem sALCL aufgeführt werden.
- **Diagnostik und Prognose:** Die Leitlinie sollte die Testung auf *DUSP22*- und *TP63*-Rearrangements als integralen Bestandteil der Diagnostik empfehlen. Dies ermöglicht eine neue risikoadaptierte Therapiestrategie: Bei Patienten mit *DUSP22*-Rearrangement und CR nach BV-CHP kann auf eine konsolidierende ASZT verzichtet werden. Bei Patienten mit *TP63*-Rearrangement sollte hingegen eine Eskalation zur allogenen SZT oder die Aufnahme in Studien mit neuen Substanzen frühzeitig erwogen werden.

**4.3 Extranodales NK/T-Zell-Lymphom, nasaler Typ (ENKTL)**

Kernaussage:

Asparaginase-haltige Regime sind der etablierte Standard in der Erstlinie. Die wichtigste Neuerung ist die Etablierung von Immun-Checkpoint-Inhibitoren als hochwirksame Option in der Therapie des R/R ENKTL.

Synthese der Evidenz:

Die Überlegenheit von Asparaginase-basierten Chemotherapien (wie DDGP oder P-GEMOX) gegenüber CHOP-ähnlichen Regimen beim ENKTL ist durch eine Vielzahl von Studien und Meta-Analysen untermauert und gilt als gesichert.^63^ Die bedeutendste Entwicklung im Beobachtungszeitraum ist die Etablierung von PD-1-Inhibitoren (z.B. Pembrolizumab, Nivolumab, Sintilimab) als eine der wirksamsten Strategien im R/R-Setting. Aufgrund der fast universellen EBV-Assoziation und der daraus resultierenden hohen PD-L1-Expression auf den Tumorzellen zeigen PD-1-Inhibitoren hohe und oft dauerhafte Ansprechraten bei Patienten, die auf eine Asparaginase-basierte Therapie nicht mehr ansprechen.^64^ Aktuelle Studien untersuchen bereits die Integration von PD-1-Inhibitoren in die Erstlinientherapie.

**Implikation für die Onkopedia-Leitlinie:**

- **Erstlinientherapie:** Die Empfehlung für Asparaginase-haltige Regime (in Kombination mit Strahlentherapie bei lokalisierten Stadien) wird beibehalten und als Standard bekräftigt.
- **Therapie des R/R ENKTL:** Die Leitlinie muss um eine neue, starke Empfehlung für den Einsatz von PD-1-Inhibitoren ergänzt werden. Diese stellen eine der wichtigsten und wirksamsten Optionen für Patienten mit rezidivierter oder refraktärer Erkrankung dar.

**4.4 Hepatosplenisches T-Zell-Lymphom (HSTL)**

Kernaussage:

Aufgrund der extremen Seltenheit und Aggressivität des HSTL gibt es keine neuen Daten aus prospektiven Studien. Die Evidenz aus Fallserien und Registern bekräftigt, dass eine intensive, nicht-Anthrazyklin-basierte Induktion mit dem Ziel einer anschließenden allogenen Stammzelltransplantation die einzige potenziell kurative Strategie darstellt.

Synthese der Evidenz:

Das HSTL bleibt eine therapeutische Herausforderung mit einer infausten Prognose unter Standard-Chemotherapie.^65^ Alle verfügbaren Daten, die primär aus retrospektiven Analysen, Registern wie dem SEER-Register und Fallserien stammen, deuten in dieselbe Richtung: Eine Heilung ist nur durch eine allogene SZT in tiefer Remission möglich.^65^ Die Induktionstherapie sollte intensiv sein und auf Regime abzielen, die bei akuten Leukämien wirksam sind, um eine schnelle und tiefe Remission zu erreichen. Vielversprechende, aber sehr frühe Daten deuten auf eine mögliche Wirksamkeit von zellulären Therapien hin; in einer Phase-I-Studie mit CD7-CAR-T-Zellen wurde auch ein HSTL-Patient behandelt und erreichte eine CR.^55^

Implikation für die Onkopedia-Leitlinie:

Die Leitlinie muss klar formulieren, dass eine CHOP-Therapie beim HSTL inadäquat ist. Die empfohlene Behandlungsstrategie muss von Beginn an auf eine allogene SZT ausgerichtet sein.

- **Therapieplan:** Die Empfehlung sollte lauten: 1. Sofortige Einleitung einer intensiven Induktionstherapie (z.B. an Leukämie-Protokolle angelehnt). 2. Parallel dazu sofortige Kontaktaufnahme mit einem Transplantationszentrum zur Spendersuche und Planung. 3. Durchführung einer allogenen SZT, sobald eine bestmögliche Remission erreicht ist.
- Die Teilnahme an Registern wie dem T-Cell Project 2.0 ^69^ oder Studien mit neuen Substanzen (z.B. CAR-T-Zellen) sollte für jeden Patienten dringend empfohlen werden.

**Works cited**

1. The fifth edition of the WHO classification of mature T cell, NK cell and stroma-derived neoplasms | Journal of Clinical Pathology, accessed August 4, 2025, <https://jcp.bmj.com/content/78/4/217.full>
2. What is new in the classification of peripheral T cell lymphomas ..., accessed August 4, 2025, <https://pmc.ncbi.nlm.nih.gov/articles/PMC10739560/>
3. The 5th edition of the World Health Organization classification of hematolymphoid tumors: T-cell lymphoma. Part 3, accessed August 4, 2025, <https://lymphomahub.com/medical-information/the-5th-edition-of-the-world-health-organization-classification-of-hematolymphoid-tumors-t-cell-lymphoma-part-3>
4. The fifth edition of the WHO classification of mature T cell, NK cell ..., accessed August 4, 2025, <https://jcp.bmj.com/content/jclinpath/78/4/217.full.pdf>
5. Introduction to the peripheral T-cell lymphoma review series: advances in molecular characterization, classification refinement and treatment optimization, accessed August 4, 2025, <https://pmc.ncbi.nlm.nih.gov/articles/PMC10690918/>
6. A Comparison of the Fifth World Health Organization and the International Consensus Classifications of Mature T-Cell Lymphomas - MDPI, accessed August 4, 2025, <https://www.mdpi.com/1422-0067/24/18/14170>
7. A Comparison of the Fifth World Health Organization and the ..., accessed August 4, 2025, <https://pmc.ncbi.nlm.nih.gov/articles/PMC10532420/>
8. Updates in the Classification of T-cell Lymphomas and Lymphoproliferative Disorders, accessed August 4, 2025, <https://mayoclinic.elsevierpure.com/en/publications/updates-in-the-classification-of-t-cell-lymphomas-and-lymphoproli>
9. Introduction to the peripheral T-cell lymphoma review series: advances in molecular characterization, classification refinement and treatment optimization | Haematologica, accessed August 4, 2025, <https://haematologica.org/article/view/haematol.2023.282719>
10. The 5th Edition of the World Health Organization Classification of Hematolymphoid Tumors - Leukemia - NCBI Bookshelf, accessed August 4, 2025, <https://www.ncbi.nlm.nih.gov/books/NBK586208/>
11. Moving Beyond One Size Fits All for T-Cell Lymphoma - ASCO Publications, accessed August 4, 2025, <https://ascopubs.org/doi/10.1200/JCO.21.02463>
12. Peripheral T‐and natural killer‐cell lymphomas: ESMO‐EHA Clinical ..., accessed August 4, 2025, <https://pmc.ncbi.nlm.nih.gov/articles/PMC12059256/>
13. Failing Forward in Peripheral T-Cell Lymphoma | Journal of Clinical Oncology, accessed August 4, 2025, <https://ascopubs.org/doi/10.1200/JCO.23.02658>
14. B- and T-/NK-Cell Lymphomas in the 2022 International Consensus Classification of Mature Lymphoid Neoplasms and Comparison with the WHO Fifth Edition - MDPI, accessed August 4, 2025, <https://www.mdpi.com/2673-6357/5/2/13>
15. NCCN Guidelines® Updates: T-Cell Lymphomas in - JNCCN, accessed August 4, 2025, <https://jnccn.org/view/journals/jnccn/20/3/article-pxxxv.xml>
16. Periphere T-Zell Lymphome - Onkopedia, accessed August 4, 2025, <https://www.onkopedia.com/de/onkopedia/guidelines/periphere-t-zell-lymphome>
17. The ECHELON-2 Trial: 5-year results of a randomized, phase III ..., accessed August 4, 2025, <https://pmc.ncbi.nlm.nih.gov/articles/PMC9447792/>
18. Full article: The expression of CD30 and its clinico-pathologic significance in peripheral T-cell lymphomas, accessed August 4, 2025, <https://www.tandfonline.com/doi/full/10.1080/17474086.2021.1955344>
19. Best Practices in CD30 Immunohistochemistry Testing, Interpretation, and Reporting, accessed August 4, 2025, <https://www.researchgate.net/publication/360211263_Best_Practices_in_CD30_Immunohistochemistry_Testing_Interpretation_and_Reporting>
20. Best Practices in CD30 Immunohistochemistry Testing, Interpretation, and Reporting: An Expert Panel Consensus - Allen Press, accessed August 4, 2025, <https://meridian.allenpress.com/aplm/article/147/1/79/480884/Best-Practices-in-CD30-Immunohistochemistry>
21. The ECHELON-2 Trial: 5-year results of a randomized, phase III study of brentuximab vedotin with chemotherapy for CD30-positive peripheral T-cell lymphoma - University of Southern Denmark, accessed August 4, 2025, <https://portal.findresearcher.sdu.dk/en/publications/the-echelon-2-trial-5-year-results-of-a-randomized-phase-iii-stud>
22. Five-Year Results from the ECHELON-2 Trial Favor A+CHP Over CHOP in CD30-Positive PTCL - Targeted Oncology, accessed August 4, 2025, <https://www.targetedonc.com/view/five-year-results-from-the-echelon-2-trial-favor-a-chp-over-chop-in-cd30-positive-ptcl>
23. E-News - September 2023 #World Lymphoma Awareness Day - Alliance for Clinical Trials in Oncology, accessed August 4, 2025, <https://www.allianceforclinicaltrialsinoncology.org/main/public/standard.xhtml?path=/Public/News-Lymphoma-Sept2023>
24. Testing the Addition of Duvelisib or CC-486 to the Usual Treatment for Peripheral T-Cell Lymphoma | ClinicalTrials.gov, accessed August 4, 2025, <https://clinicaltrials.gov/study/NCT04803201>
25. Autologous stem-cell transplantation as consolidation of first-line chemotherapy in patients with peripheral T-cell lymphoma: a multicenter GELTAMO/FIL study | Haematologica, accessed August 4, 2025, <https://www.haematologica.org/article/view/haematol.2021.279426>
26. The role of autologous stem cell transplantation in patients with nodal peripheral T-cell lymphomas in first complete remission: Report from COMPLETE, a prospective, multicenter cohort study, accessed August 4, 2025, <https://profiles.wustl.edu/en/publications/the-role-of-autologous-stem-cell-transplantation-in-patients-with>
27. Current Treatment Patterns and the Role of Upfront Autologous Stem Cell Transplantation in Patients with Peripheral T-Cell Lymphoma: A Korean Nationwide, Multicenter Prospective Registry Study (CISL 1404) - Cancer Research and Treatment, accessed August 4, 2025, <https://e-crt.org/journal/view.php?number=3435>
28. Peripheral T- and natural killer-cell lymphomas: ESMO–EHA Clinical Practice Guideline for diagnosis, treatment and follow-up | Request PDF - ResearchGate, accessed August 4, 2025, <https://www.researchgate.net/publication/391586727_Peripheral_T-_and_natural_killer-cell_lymphomas_ESMO-EHA_Clinical_Practice_Guideline_for_diagnosis_treatment_and_follow-up>
29. The Role of Allogeneic Stem Cell Transplantation in Adult Patients With Peripheral T-Cell Lymphoma: A Systematic Review and Meta-Analysis - PMC, accessed August 4, 2025, <https://pmc.ncbi.nlm.nih.gov/articles/PMC12181735/>
30. New Treatment Options in Oncology: FDA and EMA Drug Approvals in Q3 2023 – Broad Scope of Unique Targets and Malignancies - Aptitude Health, accessed August 4, 2025, <https://www.aptitudehealth.com/oncology-news/new-treatment-options-oncology-fda-ema-drug-approvals-q3-2023/>
31. Drug Approvals | IUPHAR/BPS Guide to PHARMACOLOGY, accessed August 4, 2025, <https://www.guidetopharmacology.org/GRAC/DrugApprovalsForward>
32. Adcetris | European Medicines Agency (EMA), accessed August 4, 2025, <https://www.ema.europa.eu/en/medicines/human/EPAR/adcetris>
33. Columvi | European Medicines Agency (EMA), accessed August 4, 2025, <https://www.ema.europa.eu/en/medicines/human/EPAR/columvi>
34. 761309Orig1s000 - accessdata.fda.gov, accessed August 4, 2025, <https://www.accessdata.fda.gov/drugsatfda_docs/nda/2023/761309Orig1s000MultidisciplineR.pdf>
35. FDA grants accelerated approval to axicabtagene ciloleucel for relapsed or refractory follicular lymphoma, accessed August 4, 2025, <https://www.fda.gov/drugs/resources-information-approved-drugs/fda-grants-accelerated-approval-axicabtagene-ciloleucel-relapsed-or-refractory-follicular-lymphoma>
36. Updates in the Treatment of Peripheral T-Cell Lymphomas - PMC - PubMed Central, accessed August 4, 2025, <https://pmc.ncbi.nlm.nih.gov/articles/PMC8235949/>
37. U.S. FDA Approves Kite's Tecartus™, the First and Only CAR T Treatment for Relapsed or Refractory Mantle Cell Lymphoma - Gilead Sciences, accessed August 4, 2025, <https://www.gilead.com/news/news-details/2020/us-fda-approves-kites-tecartus-the-first-and-only-car-t-treatment-for-relapsed-or-refractory-mantle-cell-lymphoma>
38. New Treatment Options in Oncology: FDA and EMA Drug Approvals in Q4 2021, accessed August 4, 2025, <https://www.aptitudehealth.com/oncology-news/fda-ema-drug-approvals-q4-2021/>
39. New FDA and EMA Drug Approvals in Q2 2023 - Aptitude Health, accessed August 4, 2025, <https://www.aptitudehealth.com/oncology-news/new-treatment-options-oncology-fda-ema-drug-approvals-q2-2023/>
40. 2021 New Drug Approvals: Review of FDA and EMA Marketing Authorisations, accessed August 4, 2025, <https://www.tribecaknowledge.com/blog/2021-new-drug-approvals-review-of-new-fda-and-ema-marketing-authorisations>
41. Novel Drug Approvals for 2023 - FDA, accessed August 4, 2025, <https://www.fda.gov/drugs/novel-drug-approvals-fda/novel-drug-approvals-2023>
42. Pipeline - LYMPHIR - Citius Pharmaceuticals, Inc., accessed August 4, 2025, <https://citiuspharma.com/pipeline/I-ONTAK/default.aspx>
43. Oncology (Cancer)/Hematologic Malignancies Approval Notifications - FDA, accessed August 4, 2025, <https://www.fda.gov/drugs/resources-information-approved-drugs/oncology-cancerhematologic-malignancies-approval-notifications>
44. Novel Drug Approvals for 2021 - FDA, accessed August 4, 2025, <https://www.fda.gov/drugs/novel-drug-approvals-fda/novel-drug-approvals-2021>
45. Bristol Myers Squibb Statement on Istodax® (romidepsin) Relapsed/Refractory Peripheral T-cell Lymphoma U.S. Indication, accessed August 4, 2025, <https://news.bms.com/news/details/2021/Bristol-Myers-Squibb-Statement-on-Istodax-romidepsin-Relapsed-Refractory-Peripheral-T-cell-Lymphoma-U.S.-Indication/default.aspx>
46. Determination That Romidepsin Injection, 10 Milligrams/2 Milliliters (5 Milligrams/Milliliter) and 27.5 Milligrams/5.5 Milliliters (5 Milligrams/Milliliter), Were Not Withdrawn From Sale for Reasons of Safety or Effectiveness - Federal Register, accessed August 4, 2025, <https://www.federalregister.gov/documents/2024/03/13/2024-05298/determination-that-romidepsin-injection-10-milligrams2-milliliters-5-milligramsmilliliter-and-275>
47. Corvus Pharmaceuticals Granted FDA Fast Track Designation for Soquelitinib for Treatment of Patients with Relapsed or Refractory Peripheral T-cell Lymphoma (PTCL), accessed August 4, 2025, <https://corvuspharma.gcs-web.com/news-releases/news-release-details/corvus-pharmaceuticals-granted-fda-fast-track-designation/>
48. Oral Azacytidine in Patients with Relapsed/Refractory Angioimmunoblastic T-Cell Lymphoma: Final Analysis of the Oracle Phase III Study | Blood | American Society of Hematology, accessed August 4, 2025, <https://ashpublications.org/blood/article/140/Supplement%201/2310/491388/Oral-Azacytidine-in-Patients-with-Relapsed>
49. Safety and efficacy of dual PI3K-δ, γ inhibitor, duvelisib in patients with relapsed or refractory lymphoid neoplasms: A systematic review and meta-analysis of prospective clinical trials - Frontiers, accessed August 4, 2025, <https://www.frontiersin.org/journals/immunology/articles/10.3389/fimmu.2022.1070660/full>
50. Duvelisib plus romidepsin in relapsed/refractory T cell lymphomas: a phase 1b/2a trial, accessed August 4, 2025, <https://pubmed.ncbi.nlm.nih.gov/38886623/>
51. The efficacy and safety of CD7 chimeric antigen receptor T-cell therapy for hematologic malignancies: a systematic review and meta-analysis - Frontiers, accessed August 4, 2025, <https://www.frontiersin.org/journals/oncology/articles/10.3389/fonc.2024.1478888/full>
52. CAR-T Cell Therapy for T-Cell Malignancies - PMC - PubMed Central, accessed August 4, 2025, <https://pmc.ncbi.nlm.nih.gov/articles/PMC10927222/>
53. CAR T-cell therapies for T-cell malignancies: does cellular immunotherapy represent the best chance of cure? | Blood Advances | American Society of Hematology, accessed August 4, 2025, <https://ashpublications.org/bloodadvances/article/9/4/913/534813/CAR-T-cell-therapies-for-T-cell-malignancies-does>
54. Paper: WU-CART-007 (WT-7), an Allogeneic CAR T-Cell Targeting CD7 in Relapsed/Refractory (R/R) T-Cell Acute Lymphoblastic Leukemia/Lymphoma (T-ALL/LBL): Phase 2 Results, accessed August 4, 2025, <https://ash.confex.com/ash/2024/webprogram/Paper202005.html>
55. Paper: CD7-Targeted CAR-T Cell Therapy Shows Promising Efficacy and Safety in Treating Refractory/Relapsed Peripheral T-Cell Lymphoma: Phase I Clinical Trial, accessed August 4, 2025, <https://ash.confex.com/ash/2024/webprogram/Paper196853.html>
56. CD7-Targeted CAR-T Cell Therapy Shows Promising Efficacy and Safety in Treating Refractory/Relapsed Peripheral T-Cell Lymphoma: Phase I Clinical Trial, accessed August 4, 2025, <https://ashpublications.org/blood/article/144/Supplement%201/2075/529241/CD7-Targeted-CAR-T-Cell-Therapy-Shows-Promising>
57. Antitumor efficacy and safety of unedited autologous CD5.CAR T cells in relapsed/refractory mature T-cell lymphomas - PubMed, accessed August 4, 2025, <https://pubmed.ncbi.nlm.nih.gov/38145560/>
58. CAR T cell therapy for T cell lymphoma shows promise in phase I trial | BCM, accessed August 4, 2025, <https://www.bcm.edu/news/car-t-cell-therapy-for-t-cell-lymphoma-shows-promise-in-phase-i-trial>
59. Early Signals of Anti-Tumor Efficacy and Safety with Autologous CD5.CAR T-Cells in Patients with Refractory/Relapsed T-Cell Lymphoma | Request PDF - ResearchGate, accessed August 4, 2025, <https://www.researchgate.net/publication/356502592_Early_Signals_of_Anti-Tumor_Efficacy_and_Safety_with_Autologous_CD5CAR_T-Cells_in_Patients_with_RefractoryRelapsed_T-Cell_Lymphoma>
60. Antitumor efficacy and safety of unedited autologous CD5.CAR T cells in relapsed/refractory mature T-cell lymphomas - American Society of Hematology, accessed August 4, 2025, <https://ashpublications.org/blood/article/143/13/1231/506723/Antitumor-efficacy-and-safety-of-unedited>
61. Real-world experience of combined treatment with azacitidine and romidepsin in patients with peripheral T-cell lymphoma - PMC - PubMed Central, accessed August 4, 2025, <https://pmc.ncbi.nlm.nih.gov/articles/PMC10368677/>
62. Duvelisib plus Romidepsin in relapsed/refractory T cell lymphomas: a phase 1b/2a trial - PMC - PubMed Central, accessed August 4, 2025, <https://pmc.ncbi.nlm.nih.gov/articles/PMC11862811/>
63. Treatment of extranodal NK/T-cell lymphoma: From past to future - PMC - PubMed Central, accessed August 4, 2025, <https://pmc.ncbi.nlm.nih.gov/articles/PMC9941192/>
64. Advances and challenges of immunotherapies in NK/T cell lymphomas - PMC, accessed August 4, 2025, <https://pmc.ncbi.nlm.nih.gov/articles/PMC10651691/>
65. Allo-HSCT with TBI-based preconditioning for hepatosplenic T-cell lymphoma: two case reports and systematic review of literature - Frontiers, accessed August 4, 2025, <https://www.frontiersin.org/journals/oncology/articles/10.3389/fonc.2024.1345464/full>
66. Hepatosplenic T-cell lymphoma: a rare but challenging entity - PMC, accessed August 4, 2025, <https://pmc.ncbi.nlm.nih.gov/articles/PMC7596851/>
67. Hepatosplenic T-cell lymphoma (HSTL): A SEER data analysis. | Journal of Clinical Oncology - ASCO Publications, accessed August 4, 2025, <https://ascopubs.org/doi/10.1200/JCO.2017.35.15_suppl.e19035>
68. Characteristics and distinct prognostic determinants of individuals with hepatosplenic T-cell lymphoma over the past two decades, accessed August 4, 2025, <https://pmc.ncbi.nlm.nih.gov/articles/PMC11212601/>
69. Prospective Observational International Registry of Patients With Newly Diagnosed Peripheral T Cell Lymphoma. | ClinicalTrials.gov, accessed August 4, 2025, <https://clinicaltrials.gov/study/NCT03964480>

# 9. Gemini 2.5 Pro output (English)

**Update to the Onkopedia guideline “Peripheral T-cell lymphomas”: A systematic review of practice-changing evidence (June 2021 - present)**

**Section 1: Advances in the diagnostic and molecular classification of PTCL**

Since June 2021, the diagnostic landscape of peripheral T-cell lymphomas (PTCL) has changed fundamentally. The publication of two new, largely consistent classification systems—the 5th edition of the WHO Classification of Hematologic Malignancies (WHO-HAEM5) and the International Consensus Classification (ICC)—marks a paradigm shift. This shift moves away from a purely morphological and immunophenotypic classification towards an integrated classification that establishes molecular genetic findings as defining criteria for many entities. These developments have direct implications for diagnostic practice, prognostic stratification, and the rationale for targeted therapies.

**1.1 The 2022 classification revision: A comparative analysis of the WHO 5th edition (WHO-HAEM5) and the International Consensus Classification (ICC)**

Key message:

The WHO-HAEM5 and ICC classifications published in 2022 redefine PTCL entities by integrating molecular and genetic findings. Although they are largely consistent in content, subtle differences in nomenclature and grouping lead to potential ambiguities in clinical practice and in the interpretation of study data, requiring clear positioning in the Onkopedia guideline.

**Details/Sources:**

·  **Classifications:** 5th Edition of the WHO Classification of Haematolymphoid Tumours (WHO-HAEM5) ^1^; International Consensus Classification (ICC).^2^

·  **Publication:** Online versions and accompanying publications since August 2022.^10^

·  **Key changes:** Both systems reflect a conceptual shift toward the integration of molecular findings, elevate provisional entities to definitive status, and introduce new entities.^4^ WHO-HAEM5 uses a hierarchical structure that groups entities into “families” according to anatomical location and clinical behavior, while the ICC combines all mature T/NK cell neoplasms into a single group.^6^

Level of evidence/grade of recommendation (estimated):

Level of evidence IIa, grade of recommendation B.

Implication for the Onkopedia guideline:

The parallel existence of two international classifications poses a challenge. A uniform nomenclature is essential for clinical practice and the comparability of study data. The Onkopedia guideline should clearly present the differences between WHO-HAEM5 and ICC, ideally in a comparative table. A recommendation for a preferred nomenclature in German-speaking countries should be made to ensure diagnostic consistency. At the same time, the alternative nomenclature must be explained to enable clinicians to correctly interpret international publications and recruit participants for studies. This need for “translation” between the systems underscores a new, important role for the guideline: actively managing and reducing potential systemic confusion in the clinical and scientific community.

**Table 1.1: Comparative classification of the most important nodal PTCLs**

| Entity/concept | WHO-HAEM4R (2017) | WHO-HAEM5 (2022) | International Consensus Classification (ICC) (2022) | Important changes/implications |
| --- | --- | --- | --- | --- |
| **TFH-lymphomas** | Angioimmunoblastic T-cell lymphoma (AITL) and other nodal lymphomas of TFH origin (provisional) | **Family:** Nodal T-follicular helper cell lymphomas (nTFHL) with 3 subtypes: nTFHL, angioimmunoblastic type; nTFHL, follicular type; nTFHL, NOS | **One entity:** Follicular T-cell lymphoma (TFHL) with 3 subtypes: angioimmunoblastic, follicular, and NOS subtype | Unification based on common biology (TFH phenotype, TET2/RHOA/IDH2 mutations). Diagnosis requires evidence of the TFH phenotype. Direct therapeutic relevance (see 1.2). |
| **ALK-negative ALCL** | Anaplastic large cell lymphoma, ALK-negative | Anaplastic large cell lymphoma, ALK-negative | Anaplastic large cell lymphoma, ALK-negative | Recognition of prognostically relevant genetic subtypes. The ICC defines ALCL with DUSP22 rearrangement as a distinct genetic subtype. Direct prognostic and therapeutic relevance (see 1.3). |
| **PTCL-NOS** | Peripheral T-cell lymphoma, not otherwise specified (NOS) | Peripheral T-cell lymphoma, NOS Peripheral T-cell lymphoma, NOS | Peripheres T-Zell-Lymphom, NOS | Remains a diagnosis of exclusion, but molecular subtypes (GATA3, TBX21) are introduced as desirable but not essential classifiers. Lays the foundation for future targeted studies (see 1.4). |

**1.2 Redefinition of T-follicular helper cell (TFH) lymphomas: From AITL to a uniform class**

Key message:

The most significant innovation is the conceptual merging of AITL, follicular T-cell lymphoma, and PTCL with TFH phenotype into a single biological group (TFH lymphomas). This reclassification has direct therapeutic consequences, as the detection of a TFH phenotype is considered a predictive biomarker for response to epigenetically active substances.

**Details/Sources:**

- **Study/Source:** WHO-HAEM5 ^1^ and ICC publications.^2^
- **Patient population:** Patients with nodal CD4-positive PTCL.
- **Endpoints:** Establishment of a biologically and clinically coherent disease group.
- **Rationale:** The standardization is based on common molecular characteristics, in particular the high-frequency mutations in genes involved in epigenetic regulation (*TET2*, *DNMT3A*, *IDH2*), as well as a specific mutation in the *RHOA*gene (*G17V*). ^1^ This molecular signature is rare in other PTCL subtypes and suggests a common pathogenesis.
- **Diagnosis:** Diagnosis now requires immunohistochemical detection of a TFH phenotype through the expression of at least two (preferably three) TFH-associated markers such as PD-1, ICOS, BCL6, or CXCL13.^2^

Level of evidence/grade of recommendation (estimated):

Level of evidence IIa, grade of recommendation A.

Implication for the Onkopedia guideline:

This reclassification is more than a semantic adjustment; it represents a paradigm shift that directly links diagnosis to treatment decisions. Previously, AITL was primarily diagnosed based on its classic histological appearance. A PTCL-NOS without this morphology but with TFH marker expression might have been treated with standard CHOP. The new classifications require that this case now be classified as TFH lymphoma. Since clinical data, such as the post-hoc analysis of the Ro-CHOP study or early data on azacitidine, suggest a particular sensitivity of TFH lymphomas to epigenetic therapies (HDAC inhibitors, hypomethylation agents) 11, the diagnostic classification becomes a predictive act. The Onkopedia guideline must therefore upgrade testing for TFH markers (at least PD-1 and ICOS) from “useful” to “essential” for the clarification of all nodal CD4-positive PTCL. The results of this testing provide direct information about treatment options in cases of recurrence and the rationale for participation in corresponding first-line treatment studies.

**1.3 Anaplastic large cell lymphoma (ALCL): Integration of genetic subtypes**

Key message:

The entity of ALK-negative ALCL is no longer monolithic. The identification of genetic subtypes, in particular the DUSP22 rearrangement with a good prognosis and the TP63 rearrangement with a very poor prognosis, enables molecular risk stratification for the first time, which should influence treatment decisions, especially with regard to consolidation.

**Details/Sources:**

- **Study/Source:** WHO-HAEM5 ^1^ and ICC publications.^5^
- **Patient population:** Patients with systemic ALK-negative ALCL.
- **Genetic markers:**
  - **DUSP22 rearrangement (DUSP22-r):** Found in approximately 30% of ALK-negative ALCL cases. These cases have a significantly better prognosis, often approaching that of ALK-positive ALCL. ^9^ The ICC recognizes this as a separate genetic subtype.^9^
  - **TP63 rearrangement (TP63-r):** Less common (approximately 5%), but associated with a very aggressive clinical course and poor prognosis.^9^
- **Guideline update:** The NCCN guideline (version 2.2022) notes that for ALK-negative ALCL with *DUSP22*rearrangement, therapy analogous to ALK-positive ALCL can be considered.^15^

Level of evidence/grade of recommendation (estimated):

Level of evidence IIb, grade of recommendation B.

Implication for the Onkopedia guideline:

The historical classification of ALCL into ALK-positive and ALK-negative is outdated. The molecular findings have direct clinical consequences.

A patient with DUSP22-rearranged ALCL has such a good prognosis that the need for consolidative autologous stem cell transplantation in first remission must be questioned, similar to the case with ALK-positive ALCL. Conversely, patients with a TP63 rearrangement have such a poor prognosis that standard chemotherapy must be considered insufficient; these patients should, if possible, undergo allogeneic stem cell transplantation or experimental therapies immediately. The Onkopedia guideline should therefore recommend FISH analysis for DUSP22 and TP63 rearrangements as standard diagnostics for all newly diagnosed ALK-negative ALCL. Prognostic stratification must be adjusted accordingly to: 1. Favorable prognosis (ALK-positive and DUSP22 rearranged), 2. Intermediate prognosis (“triple-negative”), and 3. Very poor prognosis (TP63 rearranged).

**1.4 Molecular subclassification of PTCL-NOS**

Key message:

Although not yet established for routine clinical use, the molecular subclassification of PTCL-NOS into GATA3 and TBX21 subtypes allows for a biologically sound division of this heterogeneous “residual category.” This development is crucial for the design of future targeted clinical trials.

**Details/Sources:**

- **Study/Source:** Gene expression studies, e.g., cited in the new classifications.^2^
- **Patient population:** Patients with PTCL-NOS.
- **Molecular subtypes:**
  - **PTCL-GATA3:** Associated with a Th2 cell profile, high genomic complexity (e.g., *TP53* alterations), activation of the PI3K signaling pathway, and a poorer prognosis.^2^
  - **PTCL-TBX21:** Associated with a Th1/cytotoxic profile, lower genomic complexity, activation of the JAK/STAT signaling pathway, and a better prognosis.^2^
- **Diagnosis:** Classification is primarily based on gene expression analyses. An immunohistochemical surrogate algorithm has been proposed, but is not yet considered a validated standard for routine diagnosis.^2^

Level of evidence/grade of recommendation (estimated):

Level of evidence III, grade of recommendation C.

Implication for the Onkopedia guideline:

It is premature to recommend routine molecular subclassification of PTCL-NOS for treatment decisions. Nevertheless, it is essential for a forward-looking guideline to describe this progress in detail. The guideline should explain that the heterogeneity of PTCL-NOS was a major cause of the failure of many clinical trials. The GATA3/ TBX21 classification provides, for the first time, a biological rationale for the design of targeted studies, e.g., with PI3K inhibitors for the GATA3 subtype or JAK inhibitors for the TBX21 subtype. The guideline should therefore state that CHO(E)P remains the standard, but that participation in clinical trials that stratify or select for these molecular subtypes is strongly recommended. Pathologists and clinicians should be made aware of these entities.

**1.5 CD30 immunohistochemistry: Beyond a rigid threshold**

Key message:

Clinical practice and new expert recommendations deviate from a rigid CD30 threshold of ≥10% for the use of brentuximab vedotin. CD30 expression should be understood as a continuum and described quantitatively (percentage and intensity) in the pathological findings to enable an informed clinical decision on a case-by-case basis.

**Details/Sources:**

- **Study/Source:** ECHELON-2 study design^17^, phase II studies, and expert consensus publications.^18^
- **Patient Population:** Patients with PTCL who are eligible for CD30-targeted therapy.
- **Endpoints/observations:**
  - The ECHELON-2 study used a cut-off of ≥10% CD30-positive tumor cells as an inclusion criterion.^17^
  - However, clinical studies and experience show a response to brentuximab vedotin (BV) even in patients with CD30 expression of <10%.^18^
  - This effect is attributed, among other things, to a “bystander effect,” in which the toxin released by the target cell also kills neighboring CD30-negative tumor cells.^18^
  - A 2022 expert panel recommendation advises reporting any degree of CD30 expression, including the estimated percentage of positive tumor cells and the staining intensity.^19^

Level of evidence/grade of recommendation (estimated):

Level of evidence IIb, grade of recommendation B.

Implication for the Onkopedia guideline:

The guideline must soften the previous, often rigid interpretation of the 10% threshold. It should be clarified that this value originates from the design of the approval study and does not represent an absolute biological limit for efficacy. The recommendation for pathological diagnosis should be updated to follow the expert recommendations from 2022: The finding should not only be “positive” or “negative,” but should quantify the percentage of positive tumor cells and the intensity (weak, moderate, strong). This provides the treating clinician with the necessary information to make an informed decision for or against BV-CHP therapy, even in patients with low CD30 expression, particularly in the context of the histological subtype and the overall clinical situation.

**Section 2: Evolution of first-line therapy for PTCL**

Developments in first-line therapy since 2021 have cemented treatment standards for some PTCL patients, while underscoring the urgency for new approaches for others. Long-term data from the ECHELON-2 study have revolutionized the treatment of CD30-positive PTCL. For CD30-negative disease, the search for an improvement over the CHOP standard remains a key challenge that is being addressed by innovative, randomized studies. At the same time, new subtype-specific analyses are sharpening the picture of the role of consolidative stem cell transplantation.

**2.1 CD30-positive PTCL: The sustained overall survival benefit of BV-CHP**

Key message:

The 5-year follow-up of the ECHELON-2 study confirms a sustained and significant overall survival benefit for brentuximab vedotin + CHP (BV-CHP) compared to CHOP. This definitively establishes BV-CHP as the standard first-line therapy for all newly diagnosed systemic CD30-positive PTCL, especially for sALCL.

**Study details/source:**

- **Study:** ECHELON-2 (5-year update)
- **Publication/presentation:** Horwitz S. et al., Annals of Oncology, 2022.^17^
- **Patient population:** n=452, patients with newly diagnosed CD30-positive PTCL (CD30 expression ≥10%), approximately 70% with systemic anaplastic large cell lymphoma (sALCL).
- **Endpoints:**
  - **5-year progression-free survival (PFS):** 51.4% (BV-CHP) vs. 43.0% (CHOP); hazard ratio (HR) 0.70 (95% CI: 0.53-0.91), p=0.0077.^22^
  - **5-year overall survival (OS):** 70.1% (BV-CHP) vs. 61.0% (CHOP); HR 0.72 (95% CI: 0.53-0.99), p=0.0424.^22^
  - **sALCL subgroup:** The benefit was particularly pronounced in the sALCL cohort, with a 5-year PFS rate of 60.6% vs. 48.4% (HR 0.55).^22^
  - **Safety:** The safety profile remained manageable. In particular, peripheral neuropathy (PN) improved or resolved in 72% of patients in the BV-CHP arm.^21^

Level of evidence/grade of recommendation (estimated):

Level of evidence Ia, grade of recommendation A.

Implication for the Onkopedia guideline:

The confirmation of a significant overall survival benefit after 5 years is a rare and extremely strong signal in the PTCL treatment landscape. This consolidates the position of BV-CHP as the undisputed standard of care. The guideline must sharpen the recommendation accordingly:

1. For all newly diagnosed, transplant-eligible patients with systemic ALCL (both ALK-positive and ALK-negative), BV-CHP should be declared the standard therapy with the highest recommendation grade (A).
2. For other CD30-positive PTCL subtypes (e.g., PTCL-NOS, AITL with CD30 expression ≥10%), BV-CHP should be formulated as the clearly preferred treatment option over CHO (E)P. The recommendation is based on the positive outcome of the overall study, even though the evidence for the non-ALCL subgroups is less robust due to smaller case numbers. However, extrapolation is justified in view of the OS advantage and is supported by international guidelines such as the NCCN.

**2.2 CD30-negative/low PTCL: The search for a successor to CHO(E)P**

Key message:

For CD30-negative PTCL, no new standard of care beyond CHO(E)P has been established since 2021. The current research strategy focuses on randomized phase II studies that test the addition of targeted agents to the CHOP backbone based on the specific biology of PTCL subtypes.

**Study details/source:**

- **Study:** Alliance A051902 (randomized phase II).^23^
- **Publication/presentation:** Study design published, recruitment since July 2021, results pending.
- **Patient population:** Patients with newly diagnosed, CD30-negative PTCL.
- **Study design:** Randomization into three arms: 1) CHO(E)P (standard), 2) CHO(E)P + oral azacitidine (CC-486), 3) CHO(E)P + duvelisib (PI3K inhibitor).
- **Rationale:** This study approach embodies the strategic shift away from “one-size-fits-all” studies. The addition of azacitidine specifically targets TFH lymphomas (such as AITL), which often have epigenetic mutations. Duvelisib targets subtypes with activation of the PI3K signaling pathway, as described in the PTCL-GATA3 subtype.^11^
- **Further evidence:** The final analysis of the Ro-CHOP study (romidepsin + CHOP), published in 2024, was negative in the overall cohort. However, an exploratory subgroup analysis suggested a PFS benefit for patients with the TFH phenotype ^13^, which retrospectively supports the rationale for biologically stratified studies.

Level of evidence/grade of recommendation (estimated):

Level of evidence III (for new combinations), grade of recommendation B (for study participation).

Implication for the Onkopedia guideline:

The guideline must state that CHO (E)P remains the standard outside of studies. However, the most important and practice-changing recommendation is to emphasize participation in clinical trials as the preferred option for all eligible patients. It is crucial not only to mention the existence of studies, but also to explain the scientific rationale behind modern study design. The guideline should explain the concept of the “add-on” strategy and the importance of biological stratification (e.g., TFH phenotype). This educates users of the guideline on why participation in trials such as Alliance A051902 is not only an option but the most scientifically sound approach to improving therapy for this patient group.

**2.3 The role of upfront consolidation with stem cell transplantation (SCT)**

Key message:

New data from large cohort studies strengthen the evidence for subtype-specific indications for autologous stem cell transplantation (ASCT) in first remission (CR1). In particular, patients with AITL consistently show a survival advantage, while the role in PTCL-NOS remains unclear and in sALCL after BV-CHP therapy remains questionable.

**Study details/source:**

- **Studies:** Several large, partly prospective, partly retrospective cohort studies have been published since 2021.
  - **Cabrera et al., *Haematologica*, 2022 (retrospective, international):** In 174 patients in CR1, ASZT was associated with significantly better 5-year PFS (63% vs. 48%) and OS (especially in advanced stages).^25^
  - **Mehta-Shah et al., *Cancer*, 2022 (prospective COMPLETE cohort):** ASZT was independently associated with improved survival in multivariate analysis (HR 0.37). The benefit was significant for patients with AITL, but not for other subtypes.^26^
  - **Yoon et al., *Cancer Res Treat*, 2023 (prospective Korean registry):** ASCT significantly improved PFS in patients with AITL, but not in patients with PTCL-NOS.^27^
- **Patient population:** Transplant-eligible patients with PTCL in CR1.
- **Endpoints:** PFS and OS, compared between patients who received ASCT and those who did not.

Level of evidence/grade of recommendation (estimated):

Level of evidence IIb, grade of recommendation B (subtype-specific).

Implication for the Onkopedia guideline:

The recommendation for consolidative ASCT must be developed from a blanket recommendation for “high-risk PTCL” to a differentiated, subtype-based recommendation. The data increasingly suggest that the benefit of ASCT is not the same for all PTCL.

1. **AITL:** For transplant-eligible patients with AITL in CR1, consolidating ASZT should be recommended as standard (strong recommendation). Several independent studies demonstrate a clear advantage here.^26^
2. **PTCL-NOS:** The data remain inconsistent. ASCT can be considered as an option, but the evidence is weaker than for AITL.^27^ The decision should be made on an individual basis.
3. **sALCL:** For patients with sALCL who achieve CR with BV-CHP, the value of ASCT is unclear and should not be recommended as routine standard treatment. The already high 5-year PFS rate of approximately 60% with BV-CHP alone calls into question the additional benefit of intensive consolidation.^22^
4. **Allo-SCT:** Allogeneic SCT in CR1 remains an option for ultra-high-risk entities such as hepatosplenic T-cell lymphoma (HSTL).^28^

**Section 3: Paradigm shift in the treatment of relapsed/refractory (R/R) PTCL**

In the area of relapsed/refractory (R/R) PTCL, the period since June 2021 has been marked less by new approvals and more by a reassessment of established substances and the emergence of highly innovative therapeutic approaches. A key event was the withdrawal of the approval for romidepsin, which underscores the increased requirements for evidence for targeted therapies. At the same time, clinical studies are providing new data on combination therapies, and the first promising results on cellular therapies point to an imminent change in the treatment sequence.

**3.1 The regulatory environment: A phase of recalibration**

Key message:

Since June 2021, there have been no new broad approvals of drugs for R/R PTCL by the EMA or FDA. A key event was the withdrawal of the PTCL indication for romidepsin in the US, reflecting increased pressure from the authorities to confirm indications based on accelerated approvals through randomized trials.

**Details/Sources:**

- **New approvals:** No new substances have been approved for the broad indication of R/R PTCL.^30^
- **Approval withdrawal:** In 2021, the indication for the HDAC inhibitor romidepsin for the treatment of R/R PTCL was voluntarily withdrawn by the manufacturer in the US. This occurred after the confirmatory Phase III study (Ro-CHOP) in the first line failed to meet its primary endpoint, meaning that the clinical benefit could not be sufficiently demonstrated to maintain the previously granted accelerated approval.^45^ The approval for cutaneous T-cell lymphoma (CTCL) remains in place.^45^
- **Positive signals:** The ITK inhibitor soquelitinib received Fast Track Designation status from the FDA in August 2024 for R/R PTCL, indicating the agency's high level of interest in this new class of substances and potentially accelerating the development process.^47^

Level of evidence/degree of recommendation (estimated):

N/A (regulatory information).

Implication for the Onkopedia guideline:

The withdrawal of romidepsin approval is an important signal. In the 2010s, several drugs for R/R PTCL received accelerated approval based on single-arm phase II studies with moderate response rates. The regulatory hurdles for confirming these approvals are now clearly higher. The guideline must update the status of romidepsin and point out that the evidence base for its use in nodal PTCL is weak. In general, a note should be added that therapies that are only approved on the basis of single-arm studies must be critically evaluated and that their position in the treatment algorithm may change. This raises users' awareness of the varying quality of evidence behind the available treatment options.

**3.2 Targeted therapies and combinations: An evidence-based reassessment**

**3.2.1 Hypomethylating agents (HMAs) in AITL: The ORACLE study**

Key message:

The randomized phase III ORACLE study did not achieve its primary endpoint of PFS improvement for oral azacitidine compared to standard therapy in R/R AITL/TFH lymphomas. However, an observed trend toward longer overall survival with better tolerability positions oral azacitidine as a relevant treatment option for selected patients.

**Study details/source:**

- **Study:** ORACLE (NCT03593018), phase III, randomized.
- **Publication/presentation:** Lemonnier F. et al., ASH 2022.^48^
- **Patient population:** n=86, patients with R/R AITL or nodal TFH lymphoma.
- **Endpoints:**
  - **Primary endpoint (PFS):** Median 5.6 months (oral azacitidine) vs. 4.8 months (investigator's choice: gemcitabine, bendamustine, romidepsin); HR 0.73, p=0.13.^48^ The endpoint was not reached.
  - **Secondary endpoint (OS):** median 18.4 months vs. 10.3 months; HR 0.557 (95% CI: 0.323-0.961).^48^
  - **Safety:** Fewer grade ≥3 adverse events in the azacitidine arm (76.2% vs. 97.7%), particularly less hematologic toxicity.^48^

Level of evidence/grade of recommendation (estimated):

Level of evidence Ib (for PFS negative), IIb (for OS signal and safety), grade of recommendation B (as an option).

Implication for the Onkopedia guideline:

The interpretation of a study with a negative primary but positive secondary endpoint is complex, especially in rare diseases, where studies are often underpowered. A strictly evidence-based approach would rule out a recommendation. However, an 8-month extension in median overall survival with better tolerability is clinically highly relevant. The guideline should present the study results in a differentiated manner: Oral azacitidine cannot be recommended as the new standard.

However, it should be listed as an important treatment option for patients with R/R AITL/TFH lymphoma, especially for those for whom avoiding toxicity is a priority (e.g., elderly or comorbid patients). The OS benefit should be considered a signal that may justify its use in individual cases.

**3.2.2 PI3K inhibitors: Reassessment of duvelisib**

Key message:

Duvelisib continues to show relevant efficacy in R/R PTCL, especially in AITL. However, the high rate of immune-mediated, sometimes severe side effects significantly limits its use and requires experienced management. Its value increasingly lies in its short-term use as a bridge to consolidation therapy.

**Study details/source:**

- **Study:** Meta-analysis and combination studies.
- **Publication/presentation:** Zhang M. et al., Frontiers in Immunology, 2022 (meta-analysis) ^49^; Horwitz S. et al., Blood, 2024 (combination with romidepsin).^50^
- **Efficacy:** The meta-analysis confirmed a pooled overall response rate (ORR) of 47% in T-NHL, with higher rates in AITL (67%) compared to other PTCL (42%).^49^
- **Toxicity:** High rates of grade ≥3 adverse events (79%), particularly diarrhea, transaminase elevation, and neutropenia, as well as a high rate of treatment discontinuations (33%) were confirmed.^49^
- **Combinations:** A phase 1b/2a study combining duvelisib with romidepsin showed high efficacy (ORR 56% in PTCL) with apparently lower hepatotoxicity compared to duvelisib monotherapy, suggesting a possible mitigation of toxicity by the combination.^50^

Level of evidence/grade of recommendation (estimated):

Level of evidence IIa, grade of recommendation C (due to toxicity).

Implication for the Onkopedia guideline:

The therapeutic window for duvelisib is narrow. The guideline must issue a clear warning regarding the toxicity profile. Use should be restricted to experienced centers that can ensure proactive management of immune-mediated side effects. Due to the high rate of treatment discontinuation, long-term therapy is often not possible. Therefore, the primary rationale for the use of duvelisib should be formulated as an effective “bridge therapy” to achieve remission prior to planned allogeneic stem cell transplantation. The promising data on combination with romidepsin should be mentioned as an experimental approach.

**3.3 The new frontier of cellular immunotherapy: CD5- and CD7-targeted CAR-T cells**

Key message:

Early clinical data on CD5- and CD7-targeted CAR-T cell therapies show impressive response rates in heavily pretreated patients with T-cell malignancies, including PTCL. Although still experimental, these therapies are establishing themselves as potentially the most effective salvage strategy and as a new, highly potent bridge to allogeneic stem cell transplantation.

**Study details/source:**

- **Studies:** Several Phase I studies, presented at ASH 2023 and 2024, among others.
- **CD7 CAR-T:**
  - **Target structure:** CD7 is expressed on most T-ALL/LBL and some PTCL.^51^
  - **Challenge:** “Fratricide” (mutual killing of CAR-T cells) and T-cell aplasia.^52^ Solutions include gene editing (e.g., CRISPR/Cas9 knockout of
- *CD7*) or protein blockers.^51^
  - **Results:** High CR rates in T-ALL/LBL (up to 91%).^54^ A first study in 5 R/R PTCL patients (including PTCL-NOS, HSTL, ENKTL) showed an ORR of 80% (3 CR, 1 PR). ^55^ Consolidation with allo-SCT appears to be crucial for the durability of the response.^51^
- **CD5 CAR-T:**
  - **Target structure:** CD5 is a pan-T cell marker.
  - **Challenge:** An innovative approach uses a CAR construct that leads to downregulation of the CD5 surface protein on the CAR-T cells themselves, thus preventing fratricide without gene editing.^57^
  - **Results:** A phase I study in 9 patients with R/R TCL (including AITL, PTCL) showed an ORR of 44% (2 CRs). Three of the four responding patients were successfully transferred to allo-HSCT. The safety profile was very favorable, with no high-grade cytokine release syndrome (CRS) or neurotoxicity.^57^

Level of evidence/grade of recommendation (estimated):

Level of evidence III, grade of recommendation C (as experimental therapy).

Implication for the Onkopedia guideline:

CAR-T cell therapies for T-cell lymphomas are revolutionizing the treatment of otherwise therapy-resistant patients. The guideline must include a new section on “Experimental cellular therapies.” The promising but early data for CD5 and CD7 CAR-T cells should be summarized. It must be clearly communicated that this is not currently a standard therapy. The key implication for clinical practice is the redefinition of the treatment sequence: For patients with R/R PTCL who are eligible for allo-SCT but cannot achieve sufficient remission, participation in a CAR-T cell trial is the most promising option for achieving deep remission as a “bridge to transplantation.” This is a forward-looking, practice-informing recommendation that prepares clinicians for the next paradigm shift.

**Table 3.1: Important Phase II/III trials with new substances for R/R PTCL (reported since June 2021)**

| Trial/ID | Drug(s) | Mechanism | Patient population (subtype) | N | Key efficacy results (ORR, CR, median PFS/OS) | Significant grade ≥3 toxicities | Implication/Comment |
| --- | --- | --- | --- | --- | --- | --- | --- |
| **ORACLE** (NCT03593018) | OraleAzacitidin | Hypomethylating agent | R/R AITL / TFH-lymphoma | 86 | ORR: 33%, CR: 12%. Median PFS: 5.6 months (vs. 4.8). Median OS: 18.4 months (vs. 10.3) ^48^ | Neutropenia (43%), thrombocytopenia (24%). Lower toxicity than standard therapy ^48^ | Primary endpoint (PFS) not met, but OS signal and favorable safety profile. Option for selected AITL patients. |
| **Duvelisib + Romidepsin** (NCT02783625) | Duvelisib + Romidepsin | PI3K-δ/γ-inhibitor + HDAC-inhibitor | R/R TCL (n=48 PTCL) | 66 | PTCL: ORR 56%, CR 44%. TFH subtype with particularly high response rate ^50^ | Neutropenia (42%), thrombocytopenia, fatigue. Less hepatotoxicity than duvelisib monotherapy ^50^ | Highly effective combination, especially for TFH lymphomas. Promising approach that should be pursued further in studies. |
| **CD7 CAR-T** (NCT04928105) | NS7CAR-T | Cellular immunotherapy | R/R PTCL (several subtypes) | 5 | ORR 80% (3 CR, 1 PR). 3/4 responders were transferred to allo-HSCT. ^55^ | CRS grade 1-2 (100%), no neurotoxicity. Hematotoxicity ^55^ | Very high response rates in a heavily pretreated population. Establishes CAR-T as a potent bridge to transplantation. |
| **CD5 CAR-T** (NCT0308190) | Autologous CD5 CAR-T | Cellular immunotherapy | R/R TCL (several subtypes) | 9 | ORR 44% (2 CR). 3/4 responders were transferred to allo-SCT ^57^ | No grade ≥3 CRS/ICANS. Hematotoxicity ^57^ | Favorable safety profile and clinical efficacy. Innovative approach to preventing fratricide. |

**Section 4: Updated recommendations for specific and rare PTCL subtypes**

General advances in diagnosis and treatment must be translated into subtype-specific recommendations in order to address the heterogeneity of PTCL. The following sections summarize the practice-changing developments for the most clinically relevant entities.

**4.1 Angioimmunoblastic T-cell lymphoma (AITL)**

Key message:

The treatment of AITL should be based on two pillars: epigenetically targeted therapy in the R/R setting and consolidating autologous stem cell transplantation in first remission.

Synthesis of evidence:

As the prototype of the spectrum now classified as TFH lymphoma, AITL is characterized by a high frequency of mutations in epigenetic regulatory genes (TET2, IDH2, DNMT3A).1 This biological property makes it a primary target for epigenetically active drugs. The phase III ORACLE trial, although negative in its primary endpoint, showed a clinically relevant trend toward prolonged overall survival with oral azacitidine with good tolerability, making this substance an important option in the R/R setting. ^48^ In addition, combination studies with HDAC inhibitors (romidepsin) and either HMAs (azacitidine) or PI3K inhibitors (duvelisib) have shown particularly high response rates in TFH lymphomas, including AITL.^50^ For first-line therapy, the data on consolidating ASZT in CR1 has become significantly more robust. Several large, independent cohort studies published since 2021 consistently show a significant survival benefit (both PFS and OS) for patients with AITL who undergo transplantation in CR1.^25^

Implication for the Onkopedia guideline:

The recommendations for AITL need to be comprehensively revised.

- **First-line therapy:** The recommendation for consolidative ASCT after achieving CR1 with CHO(E)P-based induction should be upgraded from an option to a strong recommendation (recommendation grade B).
- **Treatment of R/R AITL:** Epigenetically targeted therapies should be highlighted as preferred options. Oral azacitidine should be listed as a well-tolerated option with an OS signal. Highly effective combinations (e.g., romidepsin + azacitidine) should be mentioned as promising approaches, the use of which should preferably be carried out in the context of studies or at experienced centers.

**4.2 ALK-negative anaplastic large cell lymphoma (ALK-ALCL)**

Key message:

BV-CHP is the undisputed standard first-line therapy. The new molecular risk stratification based on DUSP22 and TP63 rearrangements should guide the indication for consolidation therapy.

Synthesis of evidence:

The 5-year data from the ECHELON-2 study provide Level Ia evidence for the superiority of BV-CHP over CHOP in first-line therapy, with a significant advantage in overall survival.^17^ This confirms BV-CHP as the standard therapy. In parallel, molecular characterization of ALK-negative ALCL has led to the identification of highly relevant prognostic subgroups. The presence of a

*DUSP22* rearrangement is associated with an excellent prognosis, while a *TP63* rearrangement indicates the poorest prognosis.^4^

**Implication for the Onkopedia guideline:**

- **First-line therapy:** BV-CHP must be listed as the standard therapy with the highest recommendation grade (A) for all patients with newly diagnosed ALK-negative sALCL.
- **Diagnosis and prognosis:** The guideline should recommend testing for *DUSP22* and *TP63* rearrangements as an integral part of the diagnosis. This enables a new risk-adapted treatment strategy: In patients with *DUSP22* rearrangement and CR after BV-CHP, consolidating ASZT can be omitted. In patients with *TP63* rearrangement, however, escalation to allogeneic SCT or inclusion in studies with new substances should be considered at an early stage.

**4.3 Extranodal NK/T-cell lymphoma, nasal type (ENKTL)**

Key message:

Asparaginase-containing regimens are the established standard in first-line treatment. The most important innovation is the establishment of immune checkpoint inhibitors as a highly effective option in the treatment of R/R ENKTL.

Synthesis of evidence:

The superiority of asparaginase-based chemotherapies (such as DDGP or P-GEMOX) over CHOP-like regimens in ENKTL has been confirmed by a large number of studies and meta-analyses and is considered proven.^63^ The most significant development during the observation period is the establishment of PD-1 inhibitors (e.g., pembrolizumab, nivolumab, sintilimab) as one of the most effective strategies in the R/R setting. Due to the almost universal EBV association and the resulting high PD-L1 expression on tumor cells, PD-1 inhibitors show high and often durable response rates in patients who no longer respond to asparaginase-based therapy.^64^ Current studies are already investigating the integration of PD-1 inhibitors into first-line therapy.

**Implication for the Onkopedia guideline:**

- **First-line therapy:** The recommendation for asparaginase-containing regimens (in combination with radiation therapy for localized stages) is maintained and reaffirmed as standard.
- **Treatment of R/R ENKTL:** The guideline must be supplemented with a new, strong recommendation for the use of PD-1 inhibitors. These represent one of the most important and effective options for patients with relapsed or refractory disease.

**4.4 Hepatosplenic T-cell lymphoma (HSTL)**

Key message:

Due to the extreme rarity and aggressiveness of HSTL, there are no new data from prospective studies. Evidence from case series and registries confirms that intensive, non-anthracycline-based induction with the aim of subsequent allogeneic stem cell transplantation is the only potentially curative strategy.

Synthesis of evidence:

HSTL remains a therapeutic challenge with a poor prognosis under standard chemotherapy.^65^All available data, primarily from retrospective analyses, registries such as the SEER registry, and case series, point in the same direction: A cure is only possible through allogeneic SCT in deep remission.^65^ Induction therapy should be intensive and target regimens that are effective in acute leukemias in order to achieve rapid and deep remission. Promising but very early data suggest the potential efficacy of cellular therapies; in a phase I study with CD7 CAR-T cells, one HSTL patient was also treated and achieved CR.^55^

Implication for the Onkopedia guideline:

The guideline must clearly state that CHOP therapy is inadequate for HSTL. The recommended treatment strategy must be geared toward allogeneic SCT from the outset.

- **Treatment plan:** The recommendation should be: 1. Immediate initiation of intensive induction therapy (e.g., based on leukemia protocols). 2. At the same time, immediate contact with a transplant center to find a donor and plan the transplant. 3. Performance of allogeneic SCT as soon as the best possible remission is achieved.
- Participation in registries such as the T-Cell Project 2.0 ^69^ or studies with new substances (e.g., CAR-T cells) should be strongly recommended for every patient.

**Works cited**

70. The fifth edition of the WHO classification of mature T cell, NK cell and stroma-derived neoplasms | Journal of Clinical Pathology, accessed August 4, 2025, <https://jcp.bmj.com/content/78/4/217.full>
71. What is new in the classification of peripheral T cell lymphomas ..., accessed August 4, 2025, <https://pmc.ncbi.nlm.nih.gov/articles/PMC10739560/>
72. The 5th edition of the World Health Organization classification of hematolymphoid tumors: T-cell lymphoma. Part 3, accessed August 4, 2025, <https://lymphomahub.com/medical-information/the-5th-edition-of-the-world-health-organization-classification-of-hematolymphoid-tumors-t-cell-lymphoma-part-3>
73. The fifth edition of the WHO classification of mature T cell, NK cell ..., accessed August 4, 2025, <https://jcp.bmj.com/content/jclinpath/78/4/217.full.pdf>
74. Introduction to the peripheral T-cell lymphoma review series: advances in molecular characterization, classification refinement and treatment optimization, accessed August 4, 2025, <https://pmc.ncbi.nlm.nih.gov/articles/PMC10690918/>
75. A Comparison of the Fifth World Health Organization and the International Consensus Classifications of Mature T-Cell Lymphomas - MDPI, accessed August 4, 2025, <https://www.mdpi.com/1422-0067/24/18/14170>
76. A Comparison of the Fifth World Health Organization and the ..., accessed August 4, 2025, <https://pmc.ncbi.nlm.nih.gov/articles/PMC10532420/>
77. Updates in the Classification of T-cell Lymphomas and Lymphoproliferative Disorders, accessed August 4, 2025, <https://mayoclinic.elsevierpure.com/en/publications/updates-in-the-classification-of-t-cell-lymphomas-and-lymphoproli>
78. Introduction to the peripheral T-cell lymphoma review series: advances in molecular characterization, classification refinement and treatment optimization | Haematologica, accessed August 4, 2025, <https://haematologica.org/article/view/haematol.2023.282719>
79. The 5th Edition of the World Health Organization Classification of Hematolymphoid Tumors - Leukemia - NCBI Bookshelf, accessed August 4, 2025, <https://www.ncbi.nlm.nih.gov/books/NBK586208/>
80. Moving Beyond One Size Fits All for T-Cell Lymphoma - ASCO Publications, accessed August 4, 2025, <https://ascopubs.org/doi/10.1200/JCO.21.02463>
81. Peripheral T‐and natural killer‐cell lymphomas: ESMO‐EHA Clinical ..., accessed August 4, 2025, <https://pmc.ncbi.nlm.nih.gov/articles/PMC12059256/>
82. Failing Forward in Peripheral T-Cell Lymphoma | Journal of Clinical Oncology, accessed August 4, 2025, <https://ascopubs.org/doi/10.1200/JCO.23.02658>
83. B- and T-/NK-Cell Lymphomas in the 2022 International Consensus Classification of Mature Lymphoid Neoplasms and Comparison with the WHO Fifth Edition - MDPI, accessed August 4, 2025, <https://www.mdpi.com/2673-6357/5/2/13>
84. NCCN Guidelines® Updates: T-Cell Lymphomas in - JNCCN, accessed August 4, 2025, <https://jnccn.org/view/journals/jnccn/20/3/article-pxxxv.xml>
85. Periphere T-Zell Lymphome - Onkopedia, accessed August 4, 2025, <https://www.onkopedia.com/de/onkopedia/guidelines/periphere-t-zell-lymphome>
86. The ECHELON-2 Trial: 5-year results of a randomized, phase III ..., accessed August 4, 2025, <https://pmc.ncbi.nlm.nih.gov/articles/PMC9447792/>
87. Full article: The expression of CD30 and its clinico-pathologic significance in peripheral T-cell lymphomas, accessed August 4, 2025, <https://www.tandfonline.com/doi/full/10.1080/17474086.2021.1955344>
88. Best Practices in CD30 Immunohistochemistry Testing, Interpretation, and Reporting, accessed August 4, 2025, <https://www.researchgate.net/publication/360211263_Best_Practices_in_CD30_Immunohistochemistry_Testing_Interpretation_and_Reporting>
89. Best Practices in CD30 Immunohistochemistry Testing, Interpretation, and Reporting: An Expert Panel Consensus - Allen Press, accessed August 4, 2025, <https://meridian.allenpress.com/aplm/article/147/1/79/480884/Best-Practices-in-CD30-Immunohistochemistry>
90. The ECHELON-2 Trial: 5-year results of a randomized, phase III study of brentuximab vedotin with chemotherapy for CD30-positive peripheral T-cell lymphoma - University of Southern Denmark, accessed August 4, 2025, <https://portal.findresearcher.sdu.dk/en/publications/the-echelon-2-trial-5-year-results-of-a-randomized-phase-iii-stud>
91. Five-Year Results from the ECHELON-2 Trial Favor A+CHP Over CHOP in CD30-Positive PTCL - Targeted Oncology, accessed August 4, 2025, <https://www.targetedonc.com/view/five-year-results-from-the-echelon-2-trial-favor-a-chp-over-chop-in-cd30-positive-ptcl>
92. E-News - September 2023 #World Lymphoma Awareness Day - Alliance for Clinical Trials in Oncology, accessed August 4, 2025, <https://www.allianceforclinicaltrialsinoncology.org/main/public/standard.xhtml?path=/Public/News-Lymphoma-Sept2023>
93. Testing the Addition of Duvelisib or CC-486 to the Usual Treatment for Peripheral T-Cell Lymphoma | ClinicalTrials.gov, accessed August 4, 2025, <https://clinicaltrials.gov/study/NCT04803201>
94. Autologous stem-cell transplantation as consolidation of first-line chemotherapy in patients with peripheral T-cell lymphoma: a multicenter GELTAMO/FIL study | Haematologica, accessed August 4, 2025, <https://www.haematologica.org/article/view/haematol.2021.279426>
95. The role of autologous stem cell transplantation in patients with nodal peripheral T-cell lymphomas in first complete remission: Report from COMPLETE, a prospective, multicenter cohort study, accessed August 4, 2025, <https://profiles.wustl.edu/en/publications/the-role-of-autologous-stem-cell-transplantation-in-patients-with>
96. Current Treatment Patterns and the Role of Upfront Autologous Stem Cell Transplantation in Patients with Peripheral T-Cell Lymphoma: A Korean Nationwide, Multicenter Prospective Registry Study (CISL 1404) - Cancer Research and Treatment, accessed August 4, 2025, <https://e-crt.org/journal/view.php?number=3435>
97. Peripheral T- and natural killer-cell lymphomas: ESMO–EHA Clinical Practice Guideline for diagnosis, treatment and follow-up | Request PDF - ResearchGate, accessed August 4, 2025, <https://www.researchgate.net/publication/391586727_Peripheral_T-_and_natural_killer-cell_lymphomas_ESMO-EHA_Clinical_Practice_Guideline_for_diagnosis_treatment_and_follow-up>
98. The Role of Allogeneic Stem Cell Transplantation in Adult Patients With Peripheral T-Cell Lymphoma: A Systematic Review and Meta-Analysis - PMC, accessed August 4, 2025, <https://pmc.ncbi.nlm.nih.gov/articles/PMC12181735/>
99. New Treatment Options in Oncology: FDA and EMA Drug Approvals in Q3 2023 – Broad Scope of Unique Targets and Malignancies - Aptitude Health, accessed August 4, 2025, <https://www.aptitudehealth.com/oncology-news/new-treatment-options-oncology-fda-ema-drug-approvals-q3-2023/>
100. Drug Approvals | IUPHAR/BPS Guide to PHARMACOLOGY, accessed August 4, 2025, <https://www.guidetopharmacology.org/GRAC/DrugApprovalsForward>
101. Adcetris | European Medicines Agency (EMA), accessed August 4, 2025, <https://www.ema.europa.eu/en/medicines/human/EPAR/adcetris>
102. Columvi | European Medicines Agency (EMA), accessed August 4, 2025, <https://www.ema.europa.eu/en/medicines/human/EPAR/columvi>
103. 761309Orig1s000 - accessdata.fda.gov, accessed August 4, 2025, <https://www.accessdata.fda.gov/drugsatfda_docs/nda/2023/761309Orig1s000MultidisciplineR.pdf>
104. FDA grants accelerated approval to axicabtagene ciloleucel for relapsed or refractory follicular lymphoma, accessed August 4, 2025, <https://www.fda.gov/drugs/resources-information-approved-drugs/fda-grants-accelerated-approval-axicabtagene-ciloleucel-relapsed-or-refractory-follicular-lymphoma>
105. Updates in the Treatment of Peripheral T-Cell Lymphomas - PMC - PubMed Central, accessed August 4, 2025, <https://pmc.ncbi.nlm.nih.gov/articles/PMC8235949/>
106. U.S. FDA Approves Kite's Tecartus™, the First and Only CAR T Treatment for Relapsed or Refractory Mantle Cell Lymphoma - Gilead Sciences, accessed August 4, 2025, <https://www.gilead.com/news/news-details/2020/us-fda-approves-kites-tecartus-the-first-and-only-car-t-treatment-for-relapsed-or-refractory-mantle-cell-lymphoma>
107. New Treatment Options in Oncology: FDA and EMA Drug Approvals in Q4 2021, accessed August 4, 2025, <https://www.aptitudehealth.com/oncology-news/fda-ema-drug-approvals-q4-2021/>
108. New FDA and EMA Drug Approvals in Q2 2023 - Aptitude Health, accessed August 4, 2025, <https://www.aptitudehealth.com/oncology-news/new-treatment-options-oncology-fda-ema-drug-approvals-q2-2023/>
109. 2021 New Drug Approvals: Review of FDA and EMA Marketing Authorisations, accessed August 4, 2025, <https://www.tribecaknowledge.com/blog/2021-new-drug-approvals-review-of-new-fda-and-ema-marketing-authorisations>
110. Novel Drug Approvals for 2023 - FDA, accessed August 4, 2025, <https://www.fda.gov/drugs/novel-drug-approvals-fda/novel-drug-approvals-2023>
111. Pipeline - LYMPHIR - Citius Pharmaceuticals, Inc., accessed August 4, 2025, <https://citiuspharma.com/pipeline/I-ONTAK/default.aspx>
112. Oncology (Cancer)/Hematologic Malignancies Approval Notifications - FDA, accessed August 4, 2025, <https://www.fda.gov/drugs/resources-information-approved-drugs/oncology-cancerhematologic-malignancies-approval-notifications>
113. Novel Drug Approvals for 2021 - FDA, accessed August 4, 2025, <https://www.fda.gov/drugs/novel-drug-approvals-fda/novel-drug-approvals-2021>
114. Bristol Myers Squibb Statement on Istodax® (romidepsin) Relapsed/Refractory Peripheral T-cell Lymphoma U.S. Indication, accessed August 4, 2025, <https://news.bms.com/news/details/2021/Bristol-Myers-Squibb-Statement-on-Istodax-romidepsin-Relapsed-Refractory-Peripheral-T-cell-Lymphoma-U.S.-Indication/default.aspx>
115. Determination That Romidepsin Injection, 10 Milligrams/2 Milliliters (5 Milligrams/Milliliter) and 27.5 Milligrams/5.5 Milliliters (5 Milligrams/Milliliter), Were Not Withdrawn From Sale for Reasons of Safety or Effectiveness - Federal Register, accessed August 4, 2025, <https://www.federalregister.gov/documents/2024/03/13/2024-05298/determination-that-romidepsin-injection-10-milligrams2-milliliters-5-milligramsmilliliter-and-275>
116. Corvus Pharmaceuticals Granted FDA Fast Track Designation for Soquelitinib for Treatment of Patients with Relapsed or Refractory Peripheral T-cell Lymphoma (PTCL), accessed August 4, 2025, <https://corvuspharma.gcs-web.com/news-releases/news-release-details/corvus-pharmaceuticals-granted-fda-fast-track-designation/>
117. Oral Azacytidine in Patients with Relapsed/Refractory Angioimmunoblastic T-Cell Lymphoma: Final Analysis of the Oracle Phase III Study | Blood | American Society of Hematology, accessed August 4, 2025, <https://ashpublications.org/blood/article/140/Supplement%201/2310/491388/Oral-Azacytidine-in-Patients-with-Relapsed>
118. Safety and efficacy of dual PI3K-δ, γ inhibitor, duvelisib in patients with relapsed or refractory lymphoid neoplasms: A systematic review and meta-analysis of prospective clinical trials - Frontiers, accessed August 4, 2025, <https://www.frontiersin.org/journals/immunology/articles/10.3389/fimmu.2022.1070660/full>
119. Duvelisib plus romidepsin in relapsed/refractory T cell lymphomas: a phase 1b/2a trial, accessed August 4, 2025, <https://pubmed.ncbi.nlm.nih.gov/38886623/>
120. The efficacy and safety of CD7 chimeric antigen receptor T-cell therapy for hematologic malignancies: a systematic review and meta-analysis - Frontiers, accessed August 4, 2025, <https://www.frontiersin.org/journals/oncology/articles/10.3389/fonc.2024.1478888/full>
121. CAR-T Cell Therapy for T-Cell Malignancies - PMC - PubMed Central, accessed August 4, 2025, <https://pmc.ncbi.nlm.nih.gov/articles/PMC10927222/>
122. CAR T-cell therapies for T-cell malignancies: does cellular immunotherapy represent the best chance of cure? | Blood Advances | American Society of Hematology, accessed August 4, 2025, <https://ashpublications.org/bloodadvances/article/9/4/913/534813/CAR-T-cell-therapies-for-T-cell-malignancies-does>
123. Paper: WU-CART-007 (WT-7), an Allogeneic CAR T-Cell Targeting CD7 in Relapsed/Refractory (R/R) T-Cell Acute Lymphoblastic Leukemia/Lymphoma (T-ALL/LBL): Phase 2 Results, accessed August 4, 2025, <https://ash.confex.com/ash/2024/webprogram/Paper202005.html>
124. Paper: CD7-Targeted CAR-T Cell Therapy Shows Promising Efficacy and Safety in Treating Refractory/Relapsed Peripheral T-Cell Lymphoma: Phase I Clinical Trial, accessed August 4, 2025, <https://ash.confex.com/ash/2024/webprogram/Paper196853.html>
125. CD7-Targeted CAR-T Cell Therapy Shows Promising Efficacy and Safety in Treating Refractory/Relapsed Peripheral T-Cell Lymphoma: Phase I Clinical Trial, accessed August 4, 2025, <https://ashpublications.org/blood/article/144/Supplement%201/2075/529241/CD7-Targeted-CAR-T-Cell-Therapy-Shows-Promising>
126. Antitumor efficacy and safety of unedited autologous CD5.CAR T cells in relapsed/refractory mature T-cell lymphomas - PubMed, accessed August 4, 2025, <https://pubmed.ncbi.nlm.nih.gov/38145560/>
127. CAR T cell therapy for T cell lymphoma shows promise in phase I trial | BCM, accessed August 4, 2025, <https://www.bcm.edu/news/car-t-cell-therapy-for-t-cell-lymphoma-shows-promise-in-phase-i-trial>
128. Early Signals of Anti-Tumor Efficacy and Safety with Autologous CD5.CAR T-Cells in Patients with Refractory/Relapsed T-Cell Lymphoma | Request PDF - ResearchGate, accessed August 4, 2025, <https://www.researchgate.net/publication/356502592_Early_Signals_of_Anti-Tumor_Efficacy_and_Safety_with_Autologous_CD5CAR_T-Cells_in_Patients_with_RefractoryRelapsed_T-Cell_Lymphoma>
129. Antitumor efficacy and safety of unedited autologous CD5.CAR T cells in relapsed/refractory mature T-cell lymphomas - American Society of Hematology, accessed August 4, 2025, <https://ashpublications.org/blood/article/143/13/1231/506723/Antitumor-efficacy-and-safety-of-unedited>
130. Real-world experience of combined treatment with azacitidine and romidepsin in patients with peripheral T-cell lymphoma - PMC - PubMed Central, accessed August 4, 2025, <https://pmc.ncbi.nlm.nih.gov/articles/PMC10368677/>
131. Duvelisib plus Romidepsin in relapsed/refractory T cell lymphomas: a phase 1b/2a trial - PMC - PubMed Central, accessed August 4, 2025, <https://pmc.ncbi.nlm.nih.gov/articles/PMC11862811/>
132. Treatment of extranodal NK/T-cell lymphoma: From past to future - PMC - PubMed Central, accessed August 4, 2025, <https://pmc.ncbi.nlm.nih.gov/articles/PMC9941192/>
133. Advances and challenges of immunotherapies in NK/T cell lymphomas - PMC, accessed August 4, 2025, <https://pmc.ncbi.nlm.nih.gov/articles/PMC10651691/>
134. Allo-HSCT with TBI-based preconditioning for hepatosplenic T-cell lymphoma: two case reports and systematic review of literature - Frontiers, accessed August 4, 2025, <https://www.frontiersin.org/journals/oncology/articles/10.3389/fonc.2024.1345464/full>
135. Hepatosplenic T-cell lymphoma: a rare but challenging entity - PMC, accessed August 4, 2025, <https://pmc.ncbi.nlm.nih.gov/articles/PMC7596851/>
136. Hepatosplenic T-cell lymphoma (HSTL): A SEER data analysis. | Journal of Clinical Oncology - ASCO Publications, accessed August 4, 2025, <https://ascopubs.org/doi/10.1200/JCO.2017.35.15_suppl.e19035>
137. Characteristics and distinct prognostic determinants of individuals with hepatosplenic T-cell lymphoma over the past two decades, accessed August 4, 2025, <https://pmc.ncbi.nlm.nih.gov/articles/PMC11212601/>
138. Prospective Observational International Registry of Patients With Newly Diagnosed Peripheral T Cell Lymphoma. | ClinicalTrials.gov, accessed August 4, 2025, <https://clinicaltrials.gov/study/NCT03964480>
